# Natural Products Diversity of Marine Ascidians (Tunicates; Ascidiacea) and Successful Drugs in Clinical Development

**DOI:** 10.1007/s13659-016-0115-5

**Published:** 2017-01-17

**Authors:** Satheesh Kumar Palanisamy, N. M. Rajendran, Angela Marino

**Affiliations:** 1 Department of Chemical, Biological, Pharmaceutical and Environmental Science, University of Messina, 98166 Messina, Italy; 2 Key Laboratory of Engineering Plastics and Beijing National Laboratory for Molecular Sciences, Institute of Chemistry, Chinese Academy of Sciences, Beijing, 100190 China

**Keywords:** Cancer, Cytotoxicity, Diversity, Metabolites, Pharmacology

## Abstract

This present study reviewed the chemical diversity of marine ascidians and their pharmacological applications, challenges and recent developments in marine drug discovery reported during 1994–2014, highlighting the structural activity of compounds produced by these specimens. Till date only 5% of living ascidian species were studied from <3000 species, this study represented from family didemnidae (32%), polyclinidae (22%), styelidae and polycitoridae (11–12%) exhibiting the highest number of promising MNPs. Close to 580 compound structures are here discussed in terms of their occurrence, structural type and reported biological activity. Anti-cancer drugs are the main area of interest in the screening of MNPs from ascidians (64%), followed by anti-malarial (6%) and remaining others. FDA approved ascidian compounds mechanism of action along with other compounds status of clinical trials (phase 1 to phase 3) are discussed here in. This review highlights recent developments in the area of natural products chemistry and biotechnological approaches are emphasized.

## Introduction

The study of marine natural products (MNPs) is becoming ever more sophisticated and an increasingly collaborative effort between marine biologist, chemist and pharmacologist, which involves the discovery of new natural products to enter preclinical studies and clinical tests. Since the 1990s, several MNPs and their applications towards marine biotechnological and therapeutical potential were reported. Large numbers of bioactive compounds were dragged up from marine invertebrates, especially sponges, ascidians, bryozoans and molluscs in which some of them are approved by FDA and currently utilized in clinical trials [1].

Ascidians or sea squirts (Phylum: Chordata, Class: Ascidiacea) are also known as tunicates due to their external covering, found tied to rocks and high-current fields. There are approximately 3000 living species of ascidians were reported [2]. The production of chemical compounds is principally important for soft bodied ascidian species, which use secondary metabolites to deter predatory fishes, to compete for space, to control settlement and growth of microbial fauna and other fouling organisms. Ascidians represent the most highly evolved group of marine organisms commonly investigated for identification of MNPs and provide rich sources of bioactive secondary metabolite with promising potential biomedical applications [3–5]. So far a few novel compounds have been purified and characterized with a view of developing marine drug discovery. However, the most well known didemnins has been isolated from whole body homogenates of Caribbean ascidians belonging to the genus of *Trididenium* sp. [6]. More than 80% of new ascidians compounds contain nitrogen, and nearly 70% of nitrogenous metabolites are alkaloids [7–9]. These compounds often exhibit a range of biological activities such as cytotoxicity, antibiotic, immunosuppressive activities, inhibition of topoisomerases and cyclin kinases [10]. On the other hand, non-nitrogenous metabolites are fewer available in ascidians and also less significant. Hence, identification of the biogenetic origin of ascidian natural products is often challenging [11]. The first bioactive metabolite geranyl hydroquinone was isolated from the ascidian *Aplidium* sp. [12]; only 230 metabolites were isolated from ascidians during 1974–1992 [3].

At that time, a wide-ranging attention has focused on ascidians because of their biologically active metabolites and the chemical diversity of ascidians has become one of the most significant sources of MNPs. It has been demonstrated that marine ecosystems are essential producers of unusual chemical compounds and potent bioactivities [4, 5, 9, 13]. Nonetheless, significant research in the area of marine pharmacology is a very recent origin, and also few products (or their analogues) have already reached the market as therapeutic drugs. Indeed, ascidian-derived natural products have yielded promising drug leads, among which ecteinascidin 743 (Yondelis^®^) and dehydrodidemnin B (Aplidin^®^) are in clinical usage for the treatment of specific cancers [14, 15].

**Fig. 1:**
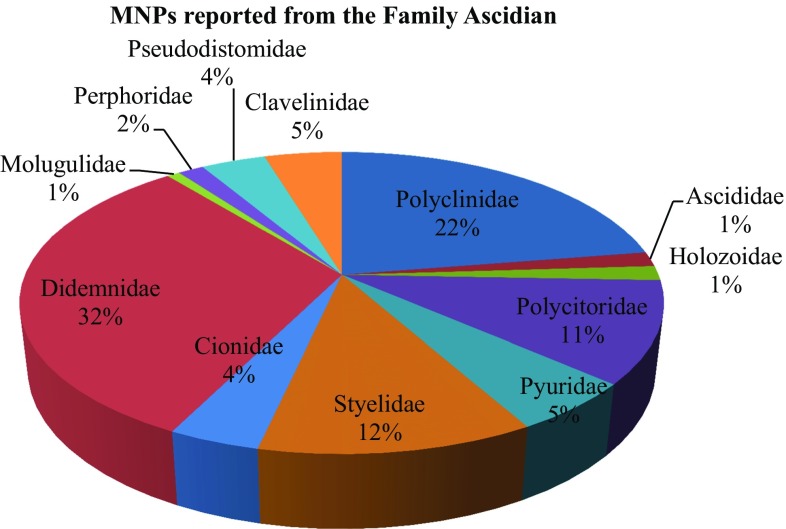
Marine nature products studied from the family ascidian on 1994–2014

**Fig. 2:**
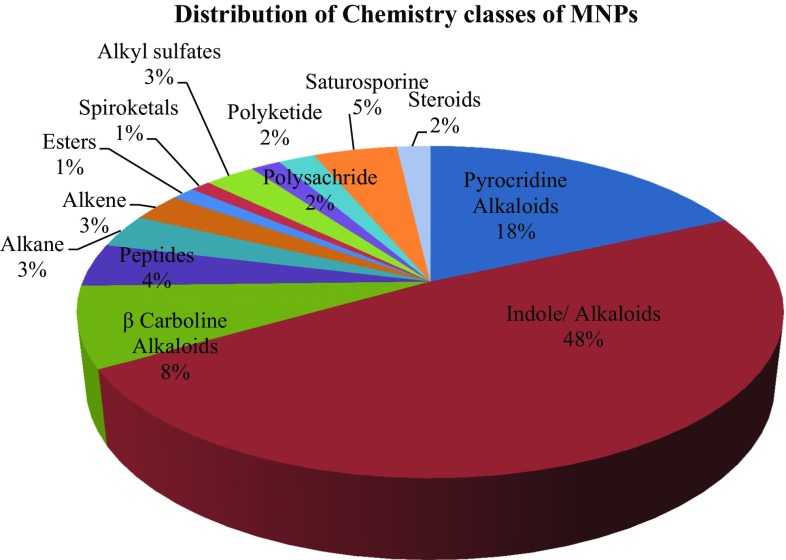
Distribution of chemistry class of MNPs with high biomedical potential application studied from 1994 to 2014

**Fig. 3:**
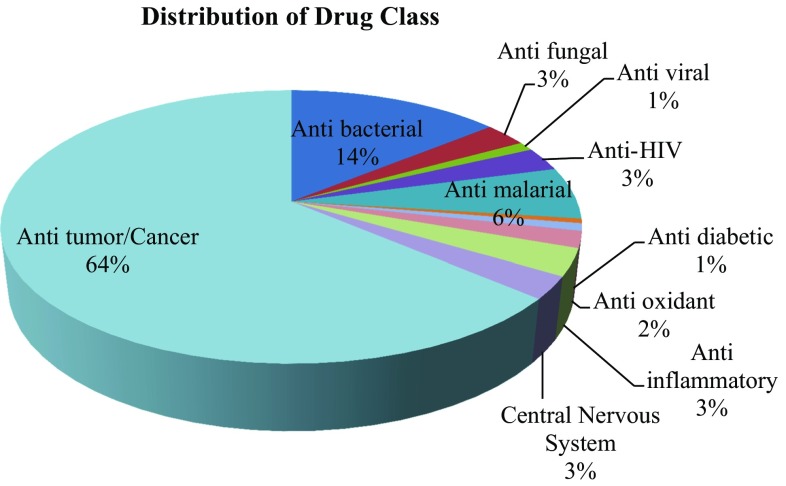
Distribution of drug classes of MNPs with high biomedical potential application studied from 1994 to 2014

The research attempt on MNPs has not targeted all marine invertebrates equally. Ascidians are one of the most intensely studied organisms during the 21st century so that 572 secondary metabolites were reported from 1994 to 2014. This present study represented MNPs studied from family didemnidae (32%), polyclinidae (22%), styelidae and polycitoridae (11–12%) exhibiting the highest number of promising MNPs (Fig. 1). The distribution of chemistry class of ascidian MNPs are given in (Fig. 2). Close to 580 compound structures are here discussed in terms of their occurrence, structural type and reported biological activity. Anti-cancer drugs are the main area of interest in the screening of MNPs from ascidians (64%), followed by anti-malarial (6%) and remaining others (Fig. 3). The National Cancer Institute-United states estimate that approximately 1% of MNPs showing anti-tumor cytotoxicity properties as against only 0.01% amongst their terrestrial counterparts. Accordingly, finding MNPs research must being continued to progress to improve existing therapies and to develop novel cures.

This review focuses on the chemical diversity of marine ascidians. The recent research on MNPs has been surveyed at relatively frequent intervals [4, 5, 9, 13]. Davidson [3] was published the first review on secondary metabolites derived ascidians from 1988 to 1993. Additionally, in contrast to the review of ascidian metabolites, the present study provides complete list of the compounds with biological activities; the primary focus of this article is addressed to structures and properties of promising ascidian metabolites and their biological activities. In this latter regard, anti-bacterial, anti-fungi, anti-diabetic, anti-viral, anti-HIV, anti-inflammatory, anti-proliferative, anti-malarial, anti-oxidant, anti-tumor, anti-cancer compounds, described in marine ascidians during 1994–2014 were reported here in. In this study, we did not aim to review proceedings of conference, scientific reports and patent literature. Wherever possible, we made attempt to focus on their potential biogenesis, chemical structure–activity relationships. Nevertheless, the present study has been mainly focused on their recent developments in the preclinical studies, biotechnology advances and future directions of ascidian secondary metabolites. Meanwhile, the possible cytotoxicity and growth inhibition of MNPs is an identifying and short-listing of a potential drug molecule. This review is restricted to those compounds exhibiting promising in vitro activity which was reported during the above period. We listed out 327 anti-tumor/cancer, 93 anti-microbial, 16 anti-HIV, 16 Central Nervous system depression, 15 anti-inflammatory compounds and other important compounds reported during the years 1994–2014.

## Natural Products from Marine Ascidians

MNPs can also be prepared by chemical synthesis method by both total synthesis and semisynthesis method and it is playing a major role in drug discovery process. In recent years’ notable studies have been carried out in the area of chemical diversity from the marine ascidians. Major alkaloids were reported in ascidians of purple, blue, green, and brown morphs of *Cystodes dellechiajei* collected from Mediterranean Sea [16]. Lopez-Legentil et al. [16] reported two distinct chemotypes in ascidian species: the purple morph of *C. dellechiajei* have the pyridoacridines shermilamine B (**1)** and kuanoniamine D (**2)** in tunic and its deacetylated forms (**3, 4)** in zooids, while the blue and green morphs comprised the C9-unsubstituted pyrridoacridines, ascididemin (**5**) and 11-hydroxyascididemin (**6**) in tunic and zooids as well. However, brown morphs consist low concentration of ascididemin. The advanced studies of the mitochondrial DNA of the distinct colour morphs of *C. dellechiajei* exhibited weak a correlation between the chemotypes, morphotypes (spicules), and genotypes with the clear relationship among the colour of the purple morph and the pyridoacridines. The purple morphs were found under the acidic conditions of tunic in the *Cystodytes* sp. 11 secondary metabolites, among which eight are indole alkaloids were reported from the ascidian *Leptoclinides* sp. [17]. The first group of *Leptoclinides*-derived indole metabolites consists of *N*-(1*H*-indolyl-3-carbonyl)-d-arginine (**7**), *N*-(6-bromo-1*H*-indolyl-3-carbonyl)-l-arginine (**8**), *N*-(6-bromo-1*H*-indolyl-3-carbonyl)-l-histidine (**9**) and *N*-(6-bromo-1*H*-indolyl-3-carbonyl)-l-enduracididine (**10**) compounds. Furthermore, the other metabolites leptoclinidamines A–C (**11**–**13**) were reported from the ascidian *L. durus* [18] and *C* ^2^-a-d-mannosylpyranosyl-l-tryptophan (**14**) was isolated from *L. dubius* [19].

A new hexacyclic pyridoacridine alkaloid, nordehydrocyclodercitin (**15**) was reported from the ascidians, *Aplidium* sp. and *A*. *cratiferum* collected in Great Barrier Reef, Australia [20]. Nordehydrocyclodercitin was structurally related to stellettamine [21] and cyclodercitin [22], which is earlier reported from the sponge metabolites. Compound cycloshermilamine D (**16**) was reported from the ascidian *Cystodytes violatinctus*, it is an analogue of stellettamine with a 6-membered non-aromatic heterocycle in place of the thiazole ring [23]. Two new pyridoacridine alkaloids kuanoniamines E and F (**17, 18**), a new ring-opened pyridoacridine alkaloid, subarine (**19**); and with known ascididemin (**5**) and kuanoniamines A and D (**20**–**22**) were reported from unidentified ascidian samples collected from the Singapore coast [24, 25].

Compound trunkamide A (**23**) was isolated from the *Lissoclinum* sp. and complete total synthesis by Wipf and Uto [26]. The chemical structures of cyclic peptides bistratamides F–I (**24**–**27**) were isolated from *L. bistratum* [27], and further confirmed by total synthesis [28, 29]. Furthermore, the metabolites didmolamides A and B (**28**–**29**) were isolated from *Didemnum molle* and complete total synthesis [30, 31]. Marine alkaloid, eudistomin X (**30**) was isolated from Micronesian ascidian *Eudistoma* sp. and the first total synthesis was achieved from phenylalanine as the chiral source [32]. Pyridoacridine alkaloids arnoamines A (**31**) and B (**32**) were isolated from the brownish purple ascidian *Cystodytes* sp. [33] and total synthesis of compounds (**31, 32**) was reported [34]. The arnoamines compounds were unusually found to incorporate deuterium at C-10 and C-11 of the pyrrole ring when dissolved in CDCl_3_/TFA-d. Piers et al. [35] reported the total synthesis of 17-methylgranulatimide (**33**) compound from photocyclization reaction of didemnimide C (**34**); and also demonstrated synthesis of isodidemnimide A (**35**), neodidemnimide A (**36**), and isogranulatimides A, B, C (**37**–**39).** The compound perophoramidine (**40**) was isolated from Philippine ascidian *Perophora namei* [36] total synthesis of compound (**40**) by halogen-selective tandem intramolecular Heck/carbonylation reaction [37]. Remarkably, perophoramidine is structurally similar to the previously reported communesins A (**41**) and B (**42**) [38–41]. Furthermore, Trieu et al. [42] reported about the total synthesis of Eudistomins Y1-Y7 (**43**–**49**), which are sub class of prevalent and biologically active *β*–carboline alkaloids, many of which have been isolated from the ascidian *Eudistoma* sp. [43] (Structure 1).

**Structure 1:**
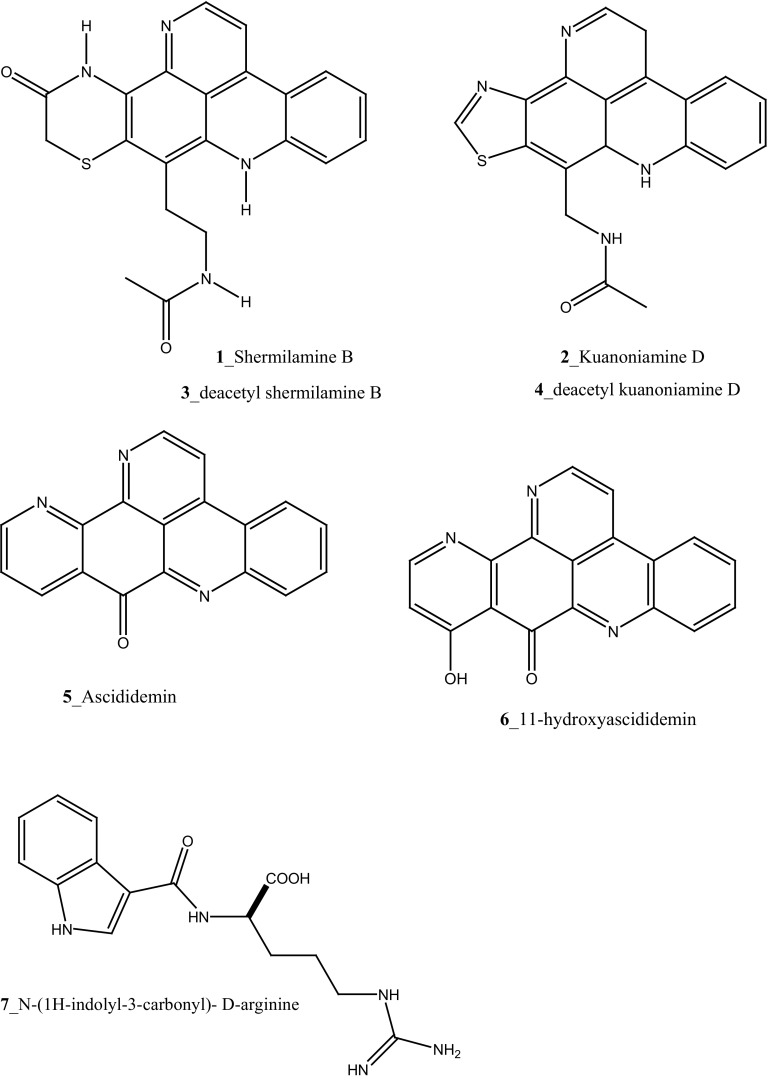

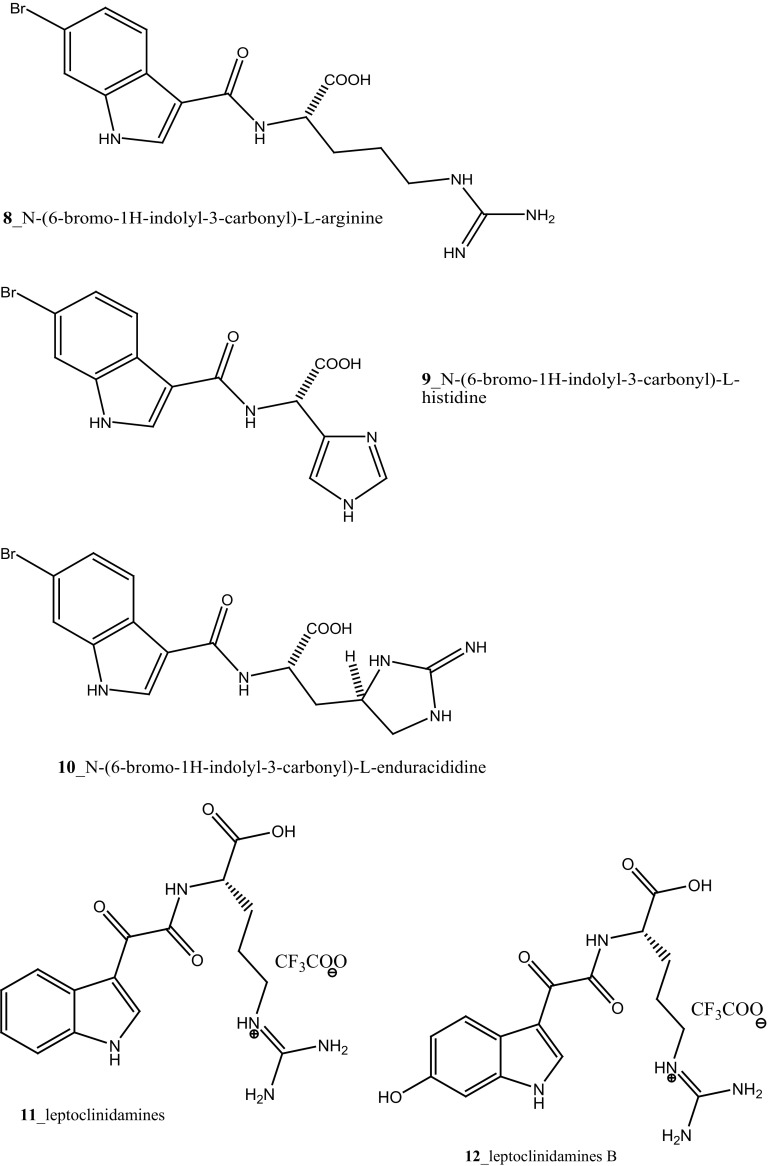

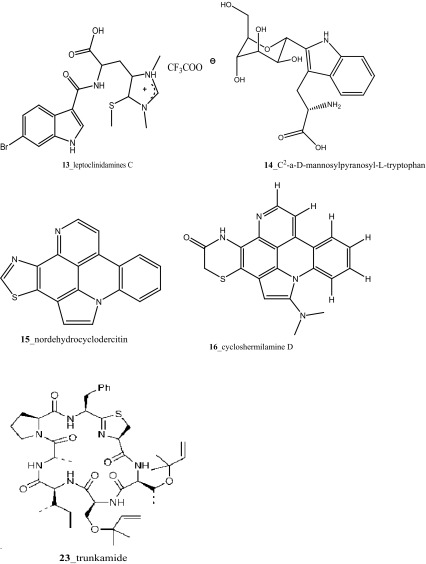

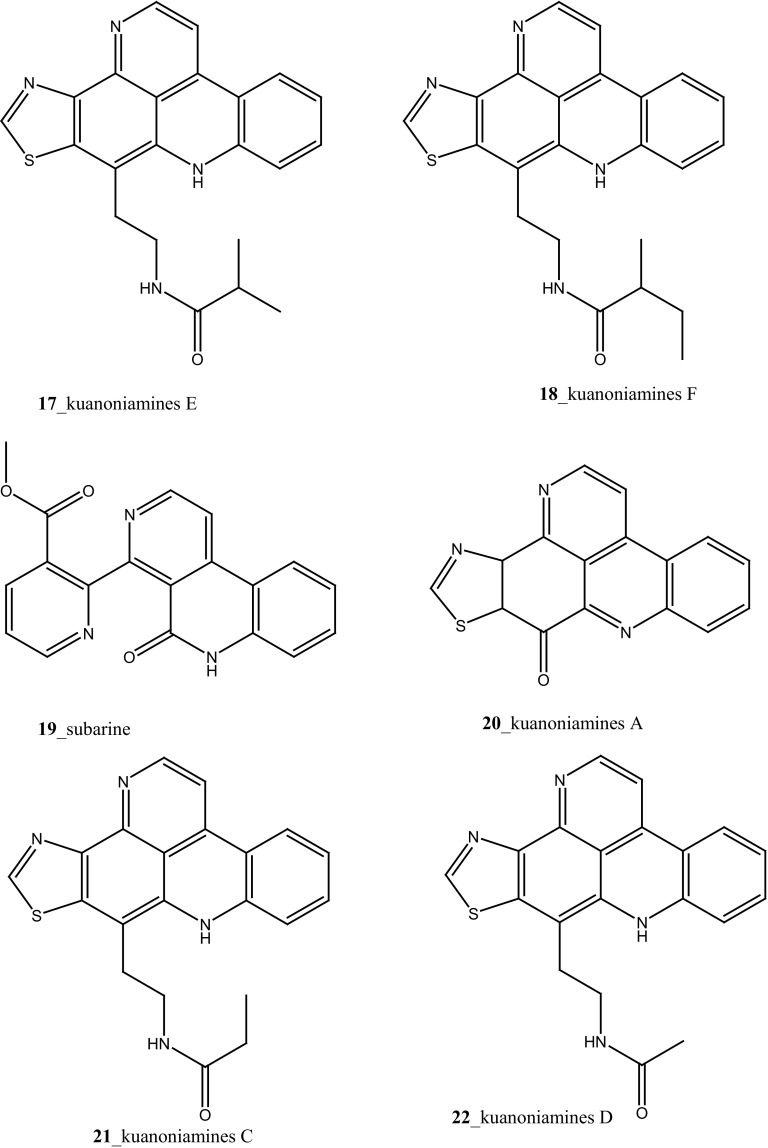

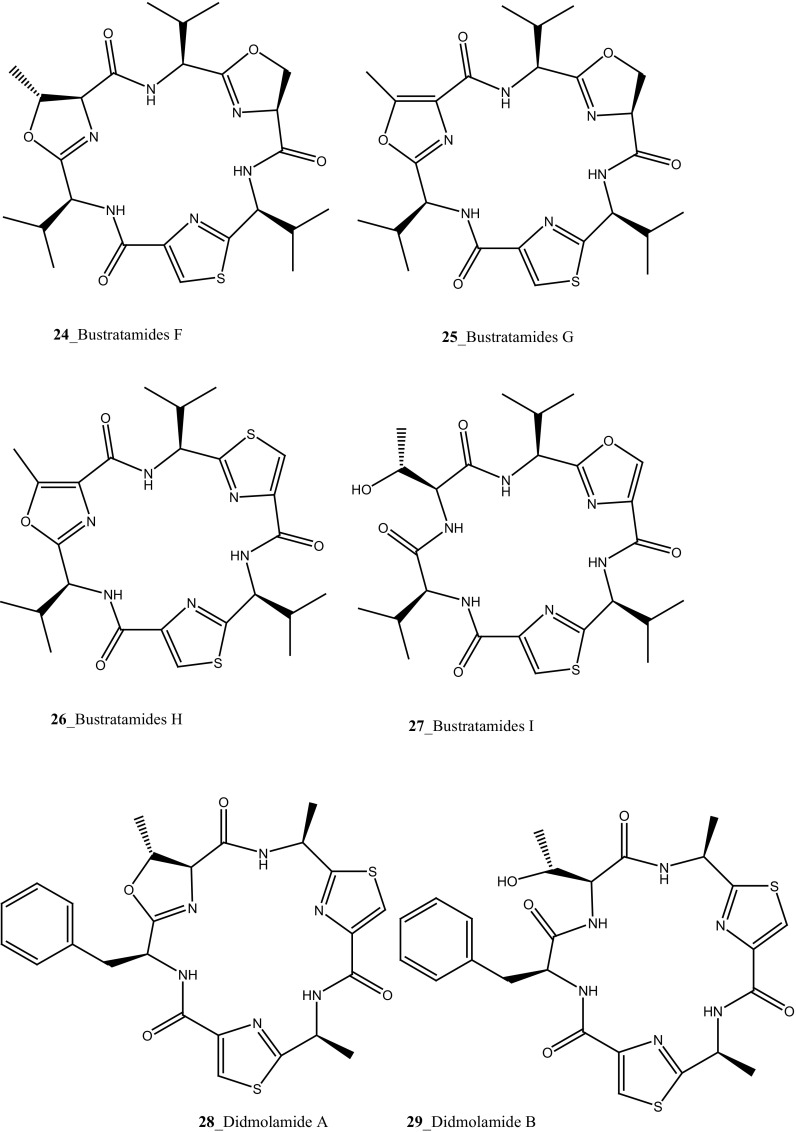

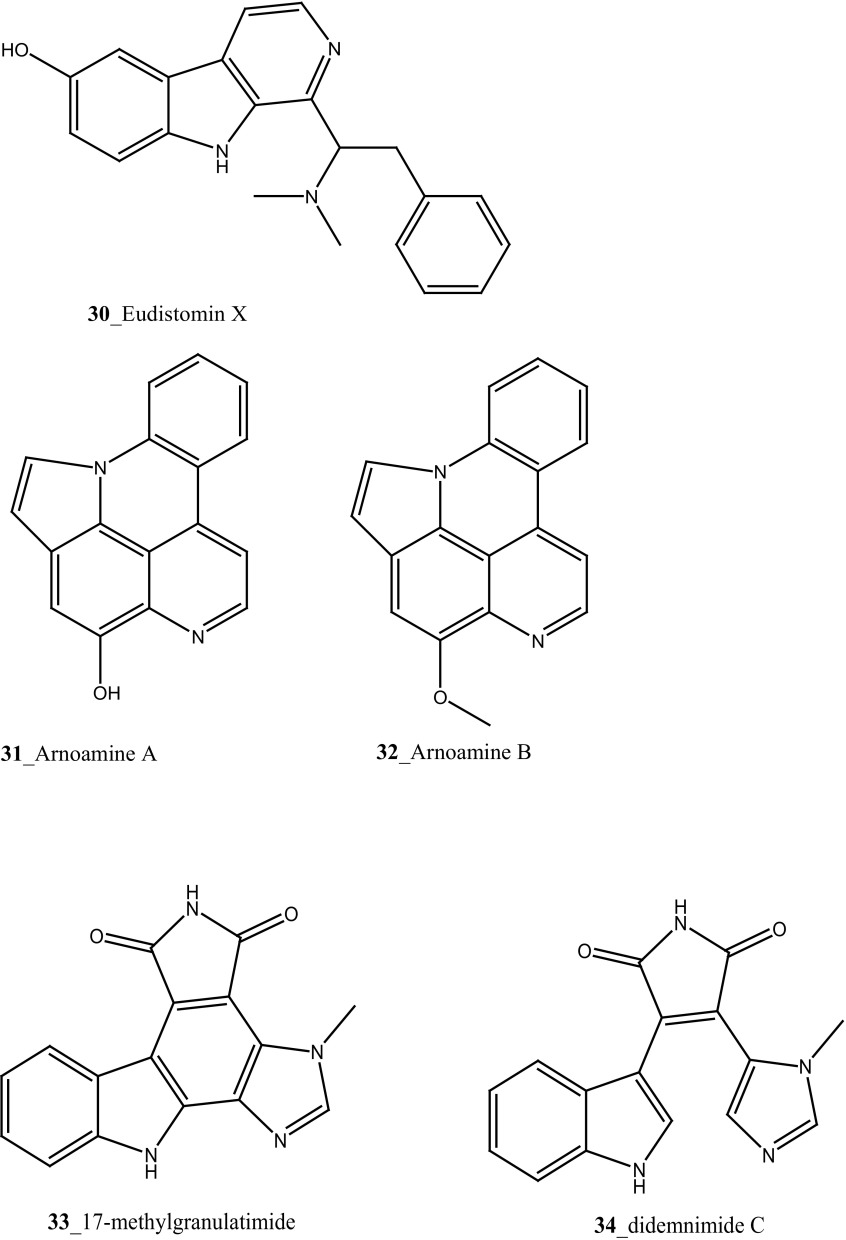

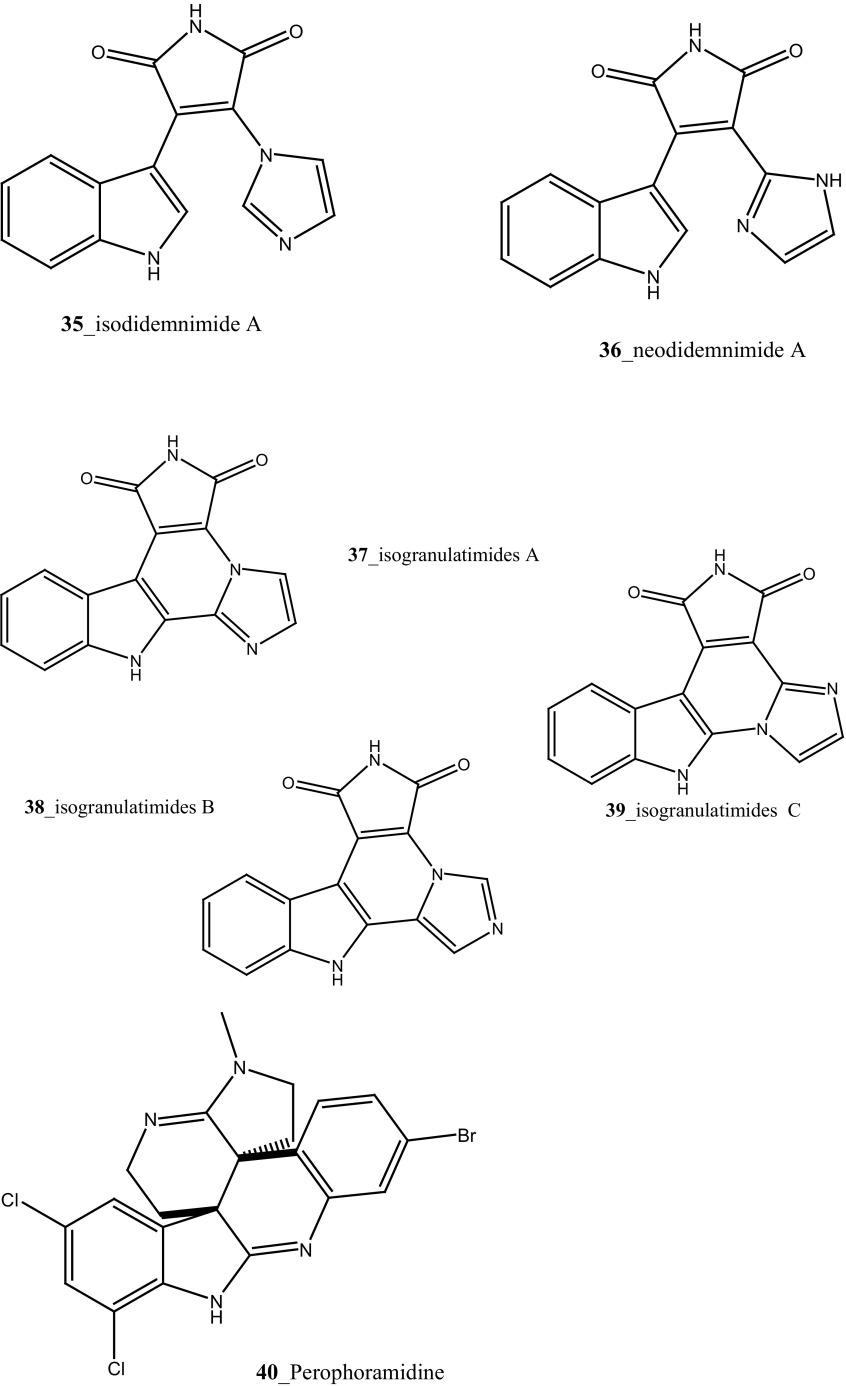

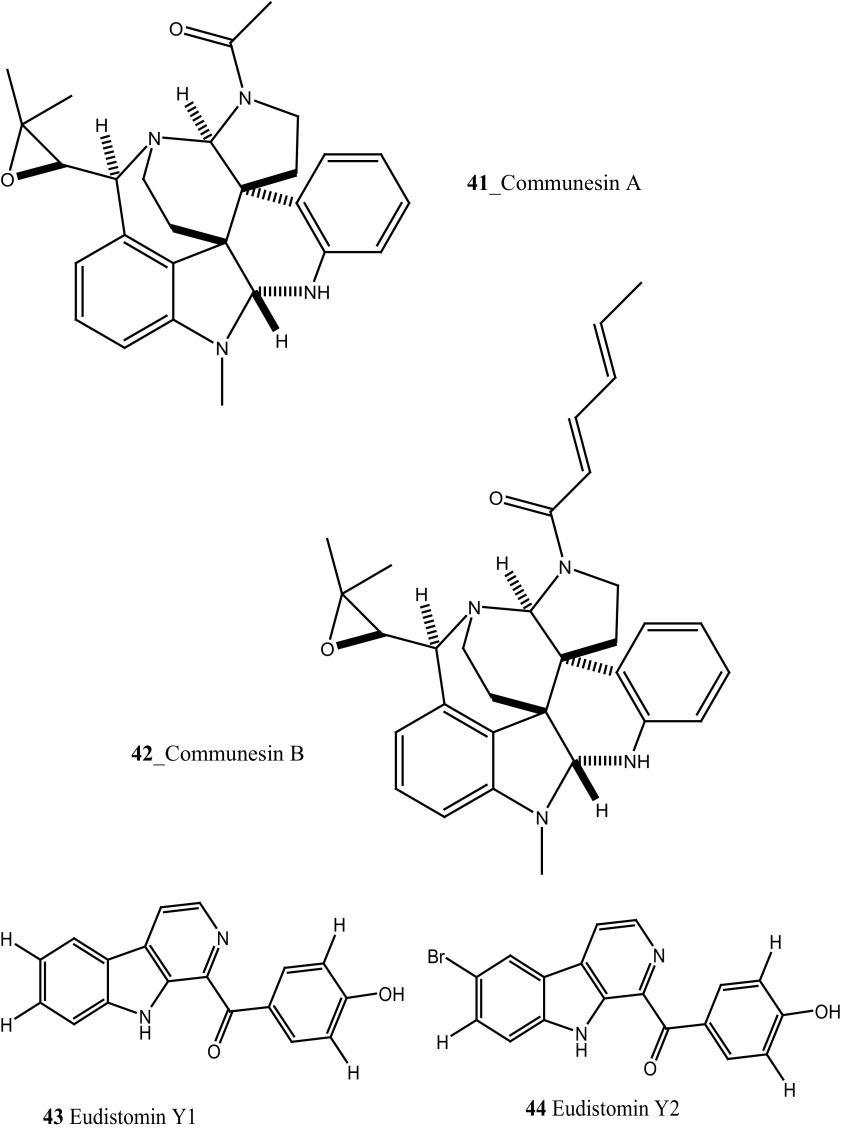

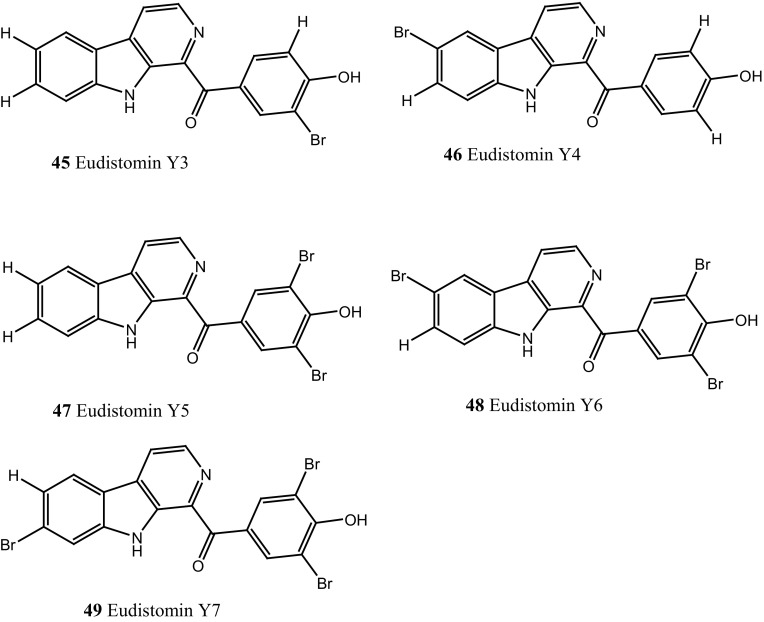
Natural products diversity of marine ascidians (compounds **1**–**49**)

## Pharmacological Activity of Ascidian Compounds

### Anti-microbial Activity

#### Anti-bacterial Activity

Four new sulphated alkanes/alkenes 2,6-dimethylheptyl sulphate, (4*Z*,7*Z*)-4,7-decadienyl sulfate, (4*Z*,7*E*)-4,7decadienyl sulphate, and (3*Z*,6*Z*)-3,6,9-decatrienyl sulphate (**50**–**53**) were isolated from the hepatopancreas of the ascidian *Halocynthia roretzi* [44]. Compounds (**50**–**53**) showed 12 mm zones of growth inhibition against bacterial strain *Vibrio alginoliticus* and besides exhibited activity with fungal strains *Mortierella ramaniana* 10 mm zones at 0.2 mg/disk, respectively. Based on the results, Tsukamoto reported these simple sulfates may possibly play a physiological role in certain ascidian species. Two new anti-microbial metabolites, halocyntin and papillosin, were isolated from hemocytes of the solitary tunicate, *H. papillosa* were collected in Catalan coast, in Mediterranea Sea [45]. Both peptides showed potential zone growth inhibition against *M. luteus* (0.13–0.25 µg/mL) and *E. coli* (0.25–0.50 µg/mL), from this compound papillosin showed the potential value of anti-microbial activity compare to halocyntin.

Amongst Ascidians, the family of Didemnidae have been described as a vital source of diverse MNPs with potent pharmacological properties. The potential important active metabolites such as didemnins, aplidine, and the tamandarins were reported from the family of Dideminidae [46]. Four new *β*-carboline based metabolites; Didemnolines (A–D) **54**–**57** were isolated from the ascidian *Didemnum* sp, collected in Northern Mariana Islands, United States [47]. Didemnolines C (**56**) shows 7 mm zone growth inhibition against *E. coli* and with *Staphyloccocus aureus* (9 mm). In addition, compound (**54)** shows potent cytotoxicity against human epidermoid carcinoma (KB cells) 0.28 µg/mL.

Three new pyridoacridine alkaloids, isodiplamine, cystodytin K and lissoclinidine (**58**–**60**), and known alkaloids diplamine (**61**) and cystodytin J (**62**) were isolated from the ascidian *Lissoclinum notti* collected at Leigh Harbour, New Zealand [48, 49]. Both compounds (**58**–**59**) were active against two marine bacteria *Psychrobactor immobilis* (1.5 µg/mL) and *Planococcus citreus* (1.5 µg/mL). Compounds (**60, 61**) showed zones of growth inhibition against *B. subtilis* (8, 9 mm) and *E. coli* (6, 3 mm) at the highest concentration 120 mg of pyridoacridine alkaloid compound, on to 6 mm paper disc. Furthermore, Pyridoacridine alkaloids inhibited the growth of fungal *T. mentagrophytes* (9, 6 mm), lissoclinidine was completely inactive against *C. albicans* and diplamine showed potent inhibition (12 mm), respectively.

Eudistomins W and X (**63**–**64**) were isolated from the colonial ascidian *Eudistoma* sp., collected in on mangrove roots at Chuuk, Micronesia [50]. Eudistomins W and X showed a moderate growth inhibition against bacterial strains *B. subtilis* (17, 18 mm), *E. coli* (15, 20 mm) and *S. aureus* (11, 12 mm) at concentration 5 and 10 µg per disc, respectively. In addition, Eudistomins W, X inhibited the zone growth against fungi *C. albicans* (13, 18 mm) at similar loading doses.

Marine alkaloids, Lissoclibadins (**65**–**67**), lissoclinotoxins E and F (**68, 69**) were isolated and reported from the ascidian *Lissoclinum* cf*. badium*, collected at coral reef station Manado, Indonesia [51, 52]. Both compounds (**65, 66**) inhibited the growth of the marine bacterium *Ruegeria atlantica* (15.2 mm and 12.2 mm at 20 mg/disc), and compound (**66**) exhibited antifungal activity towards *Mucor hiemalis* (13.8 mm at 50 mg/disk). In addition, Lissoclibadins 1–3 have also showed cytotoxic against HL-60 (IC_50_ = 0.37, 0.21, and 5.5 µM). Furthermore, four new polysulfur aromatic alkaloids, Lissoclibadins (**70**–**73**) were isolated from the same ascidian [53]. Compounds (**70**–**73**) inhibited the colony formation of Chinese hamster V79 cells with EC_50_ values of 0.71, 0.06, 0.06, and 0.17 µM, respectively. Moreover, compounds (**70**–**73**) exhibited poor anti-microbial activity against *E. coli*, *Staphylococcus aureus*, and *Saccharomyces cere*v*isiae*.

Simon-Levert et al. [54] isolated five marine meroterpenes which include two new meroterpenes, methoxyconidiol and didhydroconicol (**74, 75**) and three known derivatives, cordiachromene A (**76**), epiconicol (**77**) and conidone (**78**) from the *Aplidium aff. Densum,* collected in Masirah Island, Oman. These meroterpenes (**74, 75**) have inhibited the zone growth (MIC) against bacterial strains with *E. coli* (>2 µM) and *M. luteus* (>2 µM, 0.51 µM). Moreover, Compound (**74**) was a potential source of MNPs affecting the reproduction processes of sea urchin *Sphaerechinus granularis* and *Paracentrotus lividus*, inhibited the cleavage fertilised eggs. It interrupts M-phase development and completely blocks cytokinesis without any effect on DNA replication, most likely affecting microtubule dynamics [55]. Five new serinolipid derivatives, Shishididemniols A–E (**79**–**83**) were isolated from *Didemnum* sp. collected in Japan [56]. Compounds (**80**–**83**) exhibited a zone of growth inhibition against fish pathogenic bacterium *Vibrio anguillarum* (7.5, 7, 7 mm) at concentration 20 µg/6.5 mm discs. Wang et al. [43] reported seven new *β* carboline alkaloids, Eudistomins (Y1–Y7) from the ascidian *Eudistoma* sp. collected in South Sea, Korea. Among these metabolites, Eudistomins Y6 (**48**) showed a modest anti-bacterial activity against *Staphylococcus epidermis* and *B. subtilis* without cytotoxicity in the MTT assay at 100 µM. Consequently, eudistomins Y6 may serve as prime source for enlargement of new antibiotic drugs targeting any pathogenic strains of Gram-positive bacteria.

Three new pentacyclic alkaloids *N*-deacetylsherimilamine B (**84**) and cystodimine A, B (**85, 85a**) were isolated from Mediterranean ascidian *Cystodytes dellechiajei* collected at Catalan coast [57]. Compounds (**84, 85**) have been shown modest antibacterial growth inhibition against *E. coli* (1.1, 1.2 µg/mL) and *M. luteus* (4.5, 2.4 µg/mL), respectively. Novel bioactive alkaloid, synoxazolidinones A (**86**) and B (**87**) have been reported from the ascidian *Synoicum pulmonaria* collected in Norwegian coast [58]. Synoxazolidinone A (**86**) showed a zone of growth inhibition against bacterial strain *Corynebacterium glutamicum* (6.25 µg/mL). In addition, compound (**86**) has also inhibited growth against fungi *Saccharomyces cerevisiae* (12.5 µg/mL) at similar doses. Furthermore, synoxazolidinone C **(88)** was isolated from the Artic Ascidian *S. pulmonaria* [59]. Spectroscopic analysis proved that this compound differs from the other synoxazolidinones for a unique bicyclic partial structure, holding an additional pyrrolidine ring. Compound synoxazolidinone C **(88)** inhibited growth against gram positive bacteria *Staphylococcus aureus* at 10 µg/mL concentration, similarly to the anti-bacterial potency of synoxazolidinone B [58].

A group of known indole alkaloids, meridianins A–G (**89**–**95**) were isolated and reported from the ascidians *Aplidium meridianum* and *A. falklandicum* collected in Weddell Sea, Antarctica [60] formerly reported in *A. meridianum* collected from the South Atlantic Ocean [61, 62]. The mixture of Meridianins compounds showed a potent activity against marine bacteria (>10 mm), and exhibited a defensive role of pathogenic and fouling bacteria [63]. Furthermore, meridianins demonstrated to be responsible for the deterrent activity, against the fungal and bacterial strain.

Biologically active meroterpene compounds, rossinones A and B (**96, 96a**), were firstly reported from ascidian *Aplidium* sp. collected in Ross Sea, Antarctica [64]. In addition, Pons et al. 2012 reported rossinone B **(96a),** and their analogues 2,3-epoxy-rossinone B (**97**), 3-*epi*-rossinone B (**98**), 5,6-epoxy-rossinone B (**99**) from the same genus *A. fuegiense*, remarkably similar to this study, meroterpenes was reported earlier in sponges and seaweeds [65]. Both compounds (**96, 96a**) showed moderate biological activity of antileukemic, antiviral and anti-inflammatory properties [64]. Núñez-Pons et al. [63] reported feeding bioassay model using two predator organisms; starfish *Odontaster validus* and an amphipod *Cheirimedon femoratus* and suggested amphipod model is suitable for estimation of unpalatable chemical defence against predators in Antarctic communities. Both compounds, rossinones A and B (**96, 96a**), showed anti-inflammatory activity on human peripheral blood neutrophils, inhibited superoxide production with either N-formyl-methionylleucyl-phenylalanine (IC_50_ 1.9 and 2.5 µM), respectively.

Two new pyridoacridine alkaloids, 13-didemethylaminocycloshermilamine D (**100**), and demethyldeoxyamphimedine **(101)** were isolated from the purple ascidian *Cystodytes dellechiajei* collected at Catalonia coast, western Mediterranean Sea [66]. Both compounds **(100, 101)** showed a potent anti-microbial activity against *Listonella anguillarum* MIC (6.5–7 µM) and *Microccocus luteus* 7–9 µM, respectively.

Nine new secondary metabolites tris aromatic furanones cadiolides E–H (**102**–**105**), cadiolide I (**106**) and synoilides A and B (**107**–**108**) have been reported from the ascidian *Synoicum* sp. collected off the coast Chuja-do, Korea [67]. This new compounds possess unprecedented carbon skeletons. Cadiolide I **(106)** exhibited remarkable inhibition against various bacterial strains zone growth inhibitory (MIC) with *Kocuria rhizophila* (0.8 µM), *Salmonella enterica* (0.8 µM), and *Proteus hauseri* (3.1 µM). Moreover, synoilides A and B were completely inactive against any bacterial strain. Six *β* carboline alkaloids eudistomins Y2–Y7 (**44**–**49**) with known metabolite eudistomin Y1 (**48**) and six new derivative eudistomins Y8–Y13 (**109**–**114**) were isolated from the same ascidian [68]. Eudistomin Y10 **(111)** showed a potent inhibition against various bacterial strains such as *Bacillus subtilis* (12.5 µg/mL), *Proteus vulgaris* (12.5 µg/mL). Moreover, eudistomin Y7 **(49)** showed anti-fungal activity on *Aspergillus fumigatus* (50 µg/mL) and *Trichophyton rubrum* (50 µg/mL).

Four new antibacterial rubrolides, 3″-bromorubrolide F, 3′-bromorubrolide E, 3′-bromorubrolide F, and 3,3″-dibromorubrolide E (**115**–**118**) and earlier reported rubrolides E and F (**119**–**120**), were isolated from the ascidian *Synoicum globosum* collected from White Sands Reef at A goa Bay, South Africa [69]. Compound 3′-bromorubrolide F **(117)** showed a potent inhibition against various bacterial strains like *E. faecalis* (2 µMl, *E. coli* (14 µM). In addition, rubrolide F (**120**) exhibited activity with bacterial strains *E. coli* (15 µM). Rubrolides compounds exhibited higher values of IC_50_ in in vitro model and showed lesser value of growth inhibition than their non-methylated analogues. Furthermore, antibacterial butenolites metabolites, rubrolides P, Q (**121, 122**) and cadiolides (C–F) **123, 124, 102, 103** were isolated from the ascidian *Pseudodistoma antinboja* collected (depth of 10–15 m) off the shore of Tong-Yeong, Korea [70]. Rubrolide Q (**122**) was reported earlier as 3′-bromorubrolide F [69]. Cadiolide (C) is the most potent compound in the cadiolide series and showed a significant growth inhibition against Gram-positive bacteria *Kocuria rhizophila* (0.2 µg/mL) and *S. aureus* 0.4 µg/mL. The results of this study proved that the metabolites of the cadiolide family had potent source for developing new class of antibiotics drugs against Gram-positive bacteria compare to presently available drugs in market such as vancomycin and linezolid.

Two new spiroketals, didemnaketals D, E **(125, 126)** were isolated from the ascidian *Didemnum* sp. collected in Mangroves area in Nabq/Sharm El-Sheikh (depth 1–2 m), Red Sea, on the Egypt [71]. Both Compounds (**125, 126**) showed a protein kinase inhibitory activity against various kinases (CDK5, CK1, DyrK1A, and GSK3) at <10 mg/ml. Furthermore, metabolites (**125, 126**) exhibited a moderate antimicrobial activity, against *S. aureus* (11 mm) and *Bacillus subtilis* (11 mm), respectively.

Two novel phenylalanine-peptides styelins A, B were isolated from the hemocytes of solitary tunicate *Styela clava* collected in California coast [72]. Both the peptides had very similar m/z (Styelin A, 3685.8; Styelin B, 3700.6). Styelins A and B showed a significant inhibition against bacterial pathogens of humans (MIC 0.5 µM). Styelins killed marine bacteria, *Psychrobacter immobilis* and *Planococcus citreus*, in media containing 0.4 mM NaCl. Moreover, histidine rich amidated 23-residue peptide clavaspirin (FLRF_IG_SVIHGIGHLVHHIGVAL-NH_2_) was identified by cloning of a peptide cDNA from the pharyngeal tissues of *S. clava* [72].

A modified octapeptide, plicatamide (**127**) and their analogues were isolated from the hemolymph of ascidian *Styela plicata* collected at San Diego Bay [73]. Compound (**127**) represents a significant link between two classes of biomolecules, the tunichromes which shares an oxidatively decarboxylated C-terminus and higher molecular weight DOPA- polypeptides. All the synthetic analogues have showed a significant anti-microbial activity, causing K + efflux in *S. aureus*. In addition, it exhibited haemolytic effect on human red blood cells (RBC) and formed cation-selective channels in model lipid bilayers [74].

Lindsay et al. (1995) have isolated pyrido [2,3,4-*kl*] acridine-based alkaloids ascididemin 2 from the ascidian *Didemnum* sp. Ascididemin 2 (**5a**) inhibited growth of *B. subtilis* (14 mm) and *E. coli* (10 mm) and was completely inactive against the *Pseudomonas aeruginosa*. Furthermore, Ascididemin 2 showed anti-fungal activity against *Cladisporium resinae* (10 mm) and *C. albicans* inhibition (11 mm). Namenamicin (**128**) was isolated from the orange ascidian *Polysyncraton lithostrotum* from Namenalala [75]. Compound (**128**) showed a potential inhibition of *B. subtilis* (0.03 µg/mL), *S. aureus* (0.03 µg/mL), and *E. coli* (0.12 µg/mL), respectively. Additionally, namenamicin also inhibited the zone growth against fungi *Ustilago maydis* (0.004 µg/mL) and *Saccharomyces cerevisiae* (0.06 µg/mL) (Structure 2).

**Structure 2:**
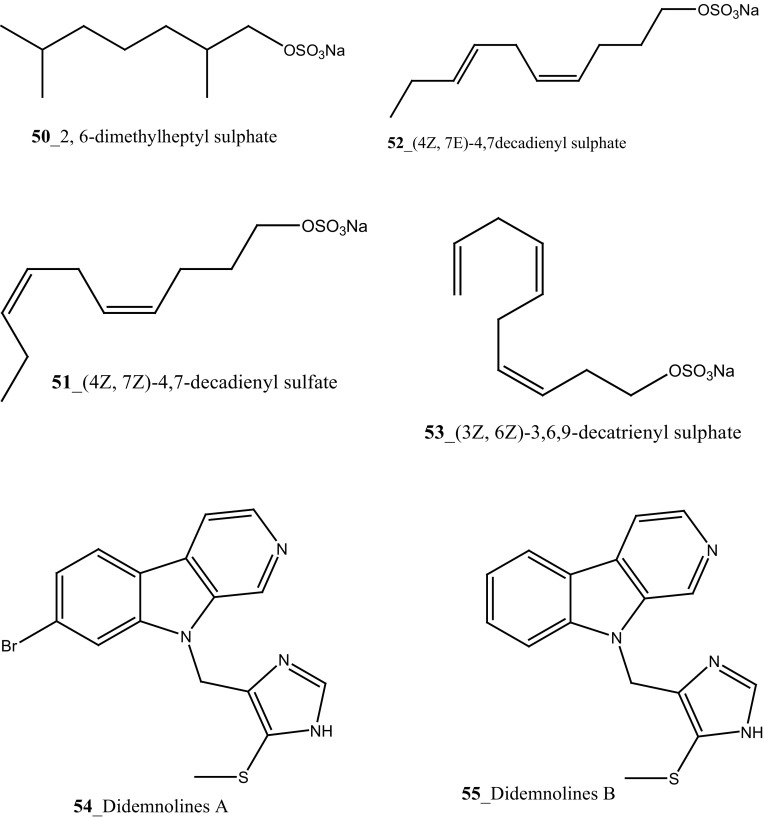

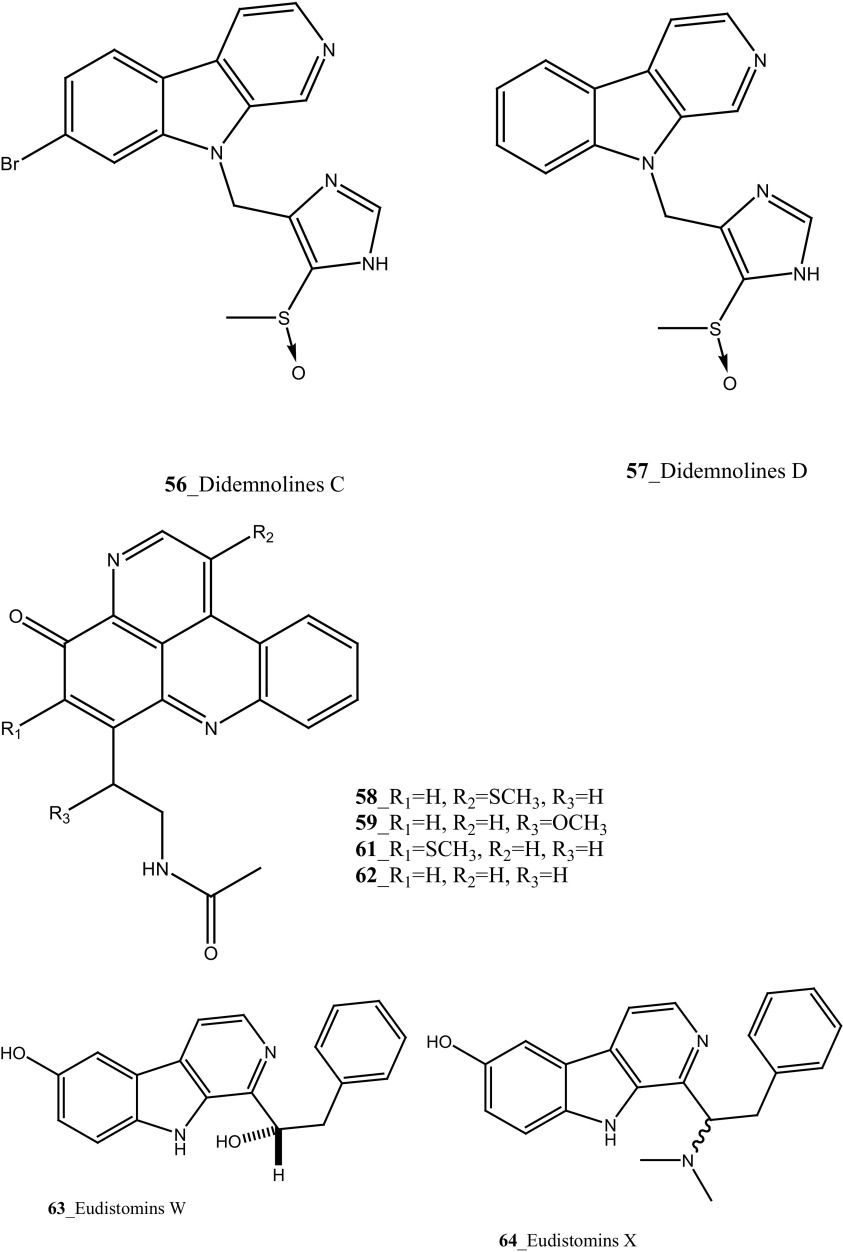

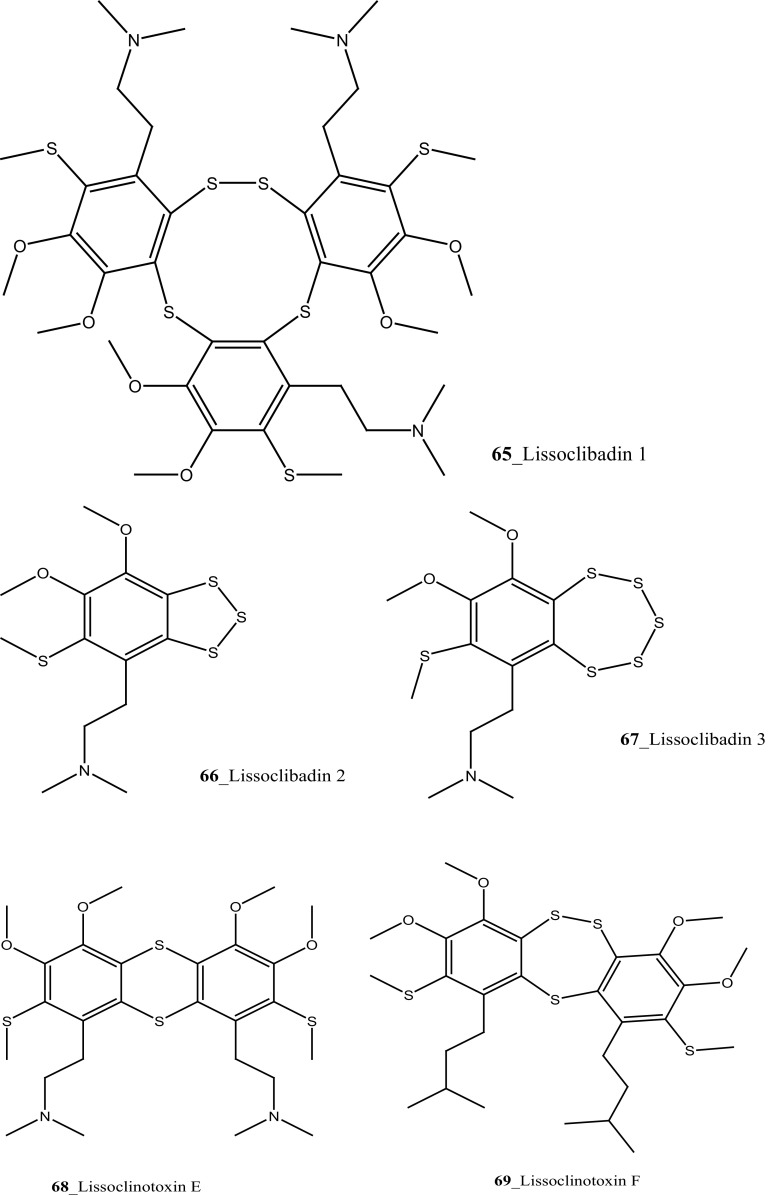

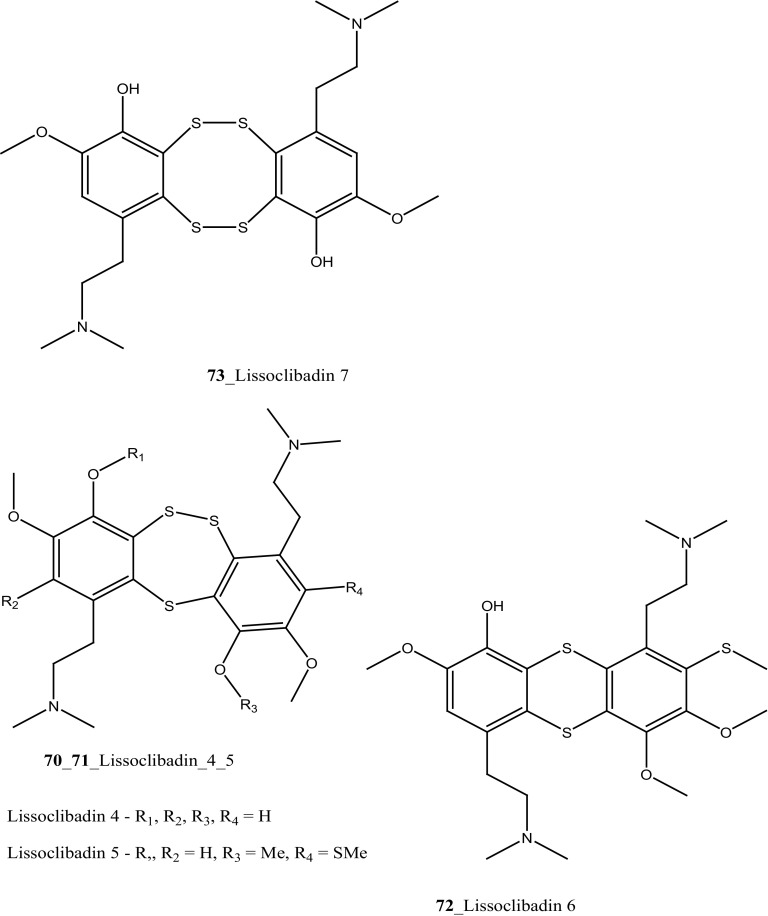

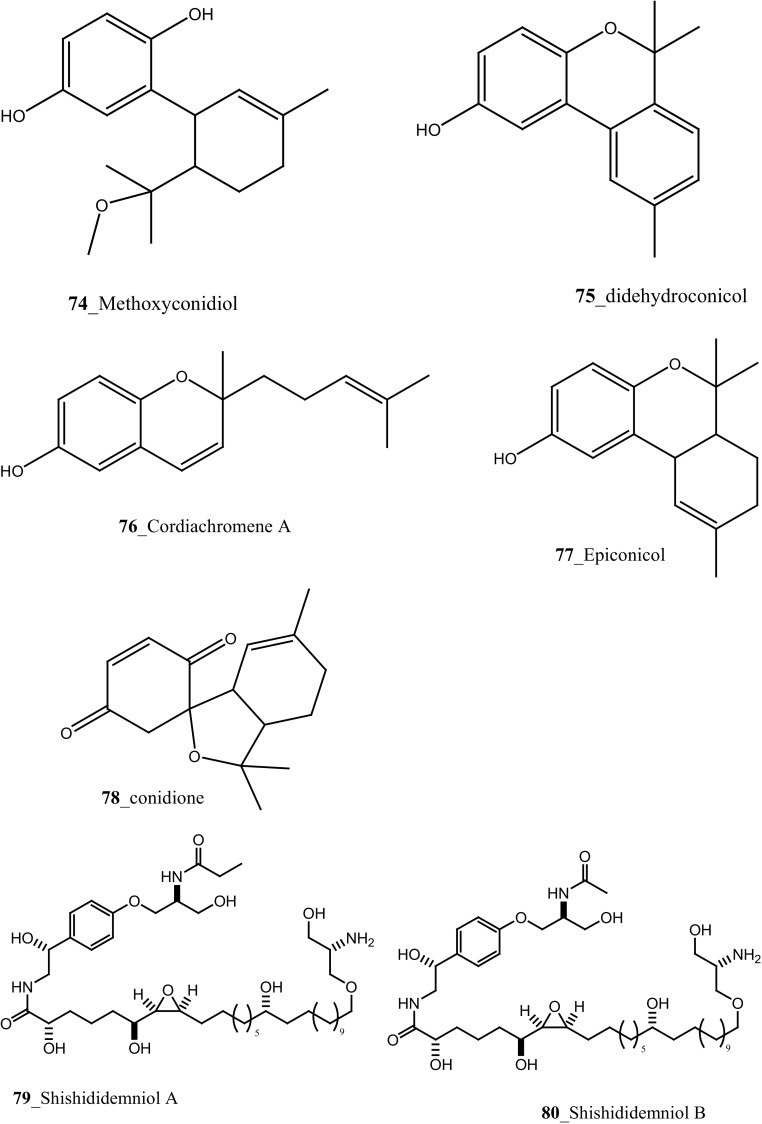

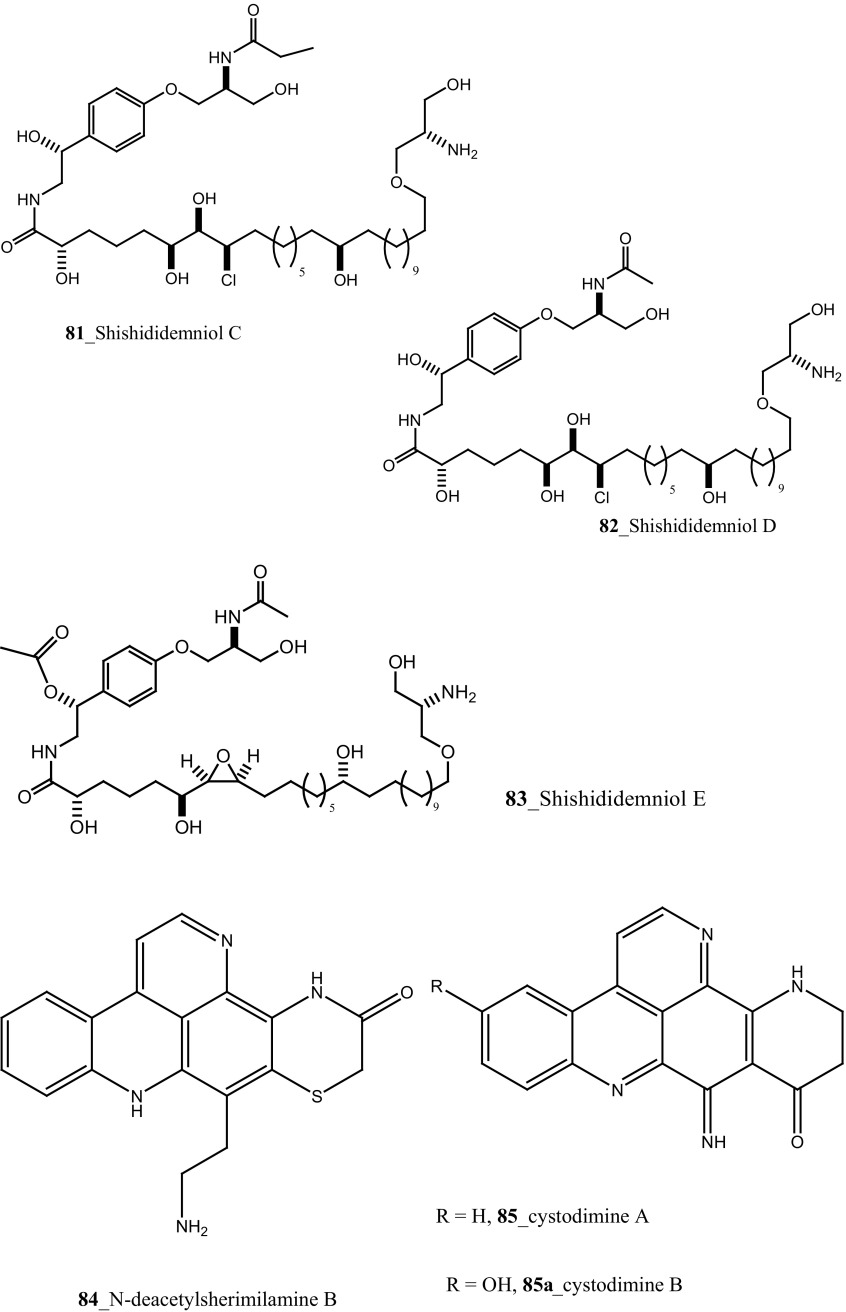

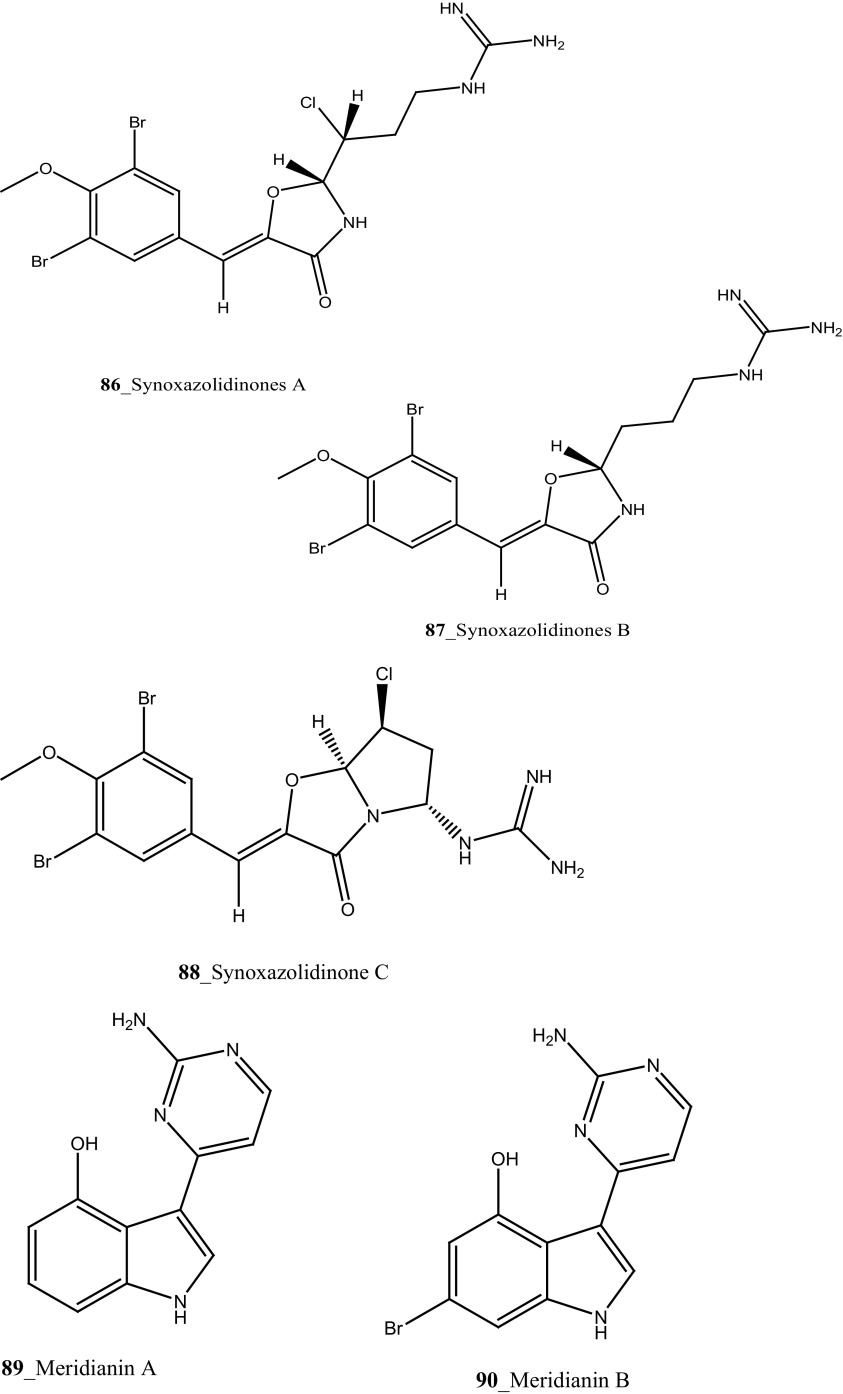

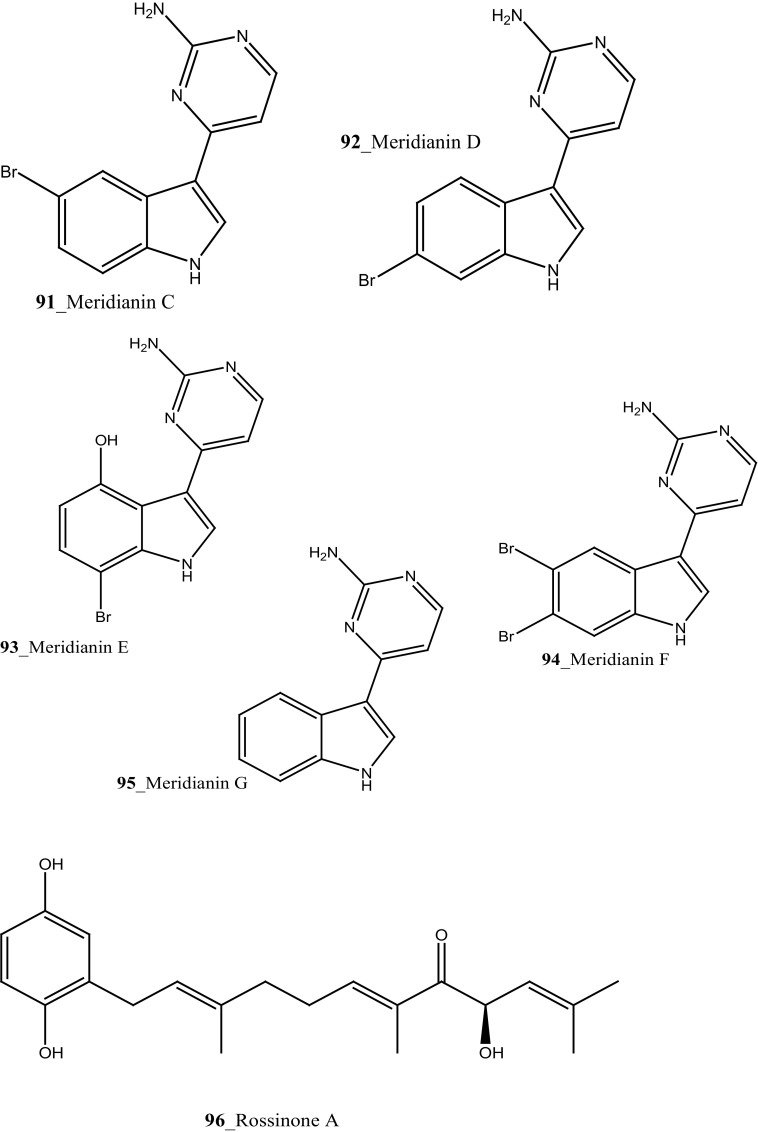

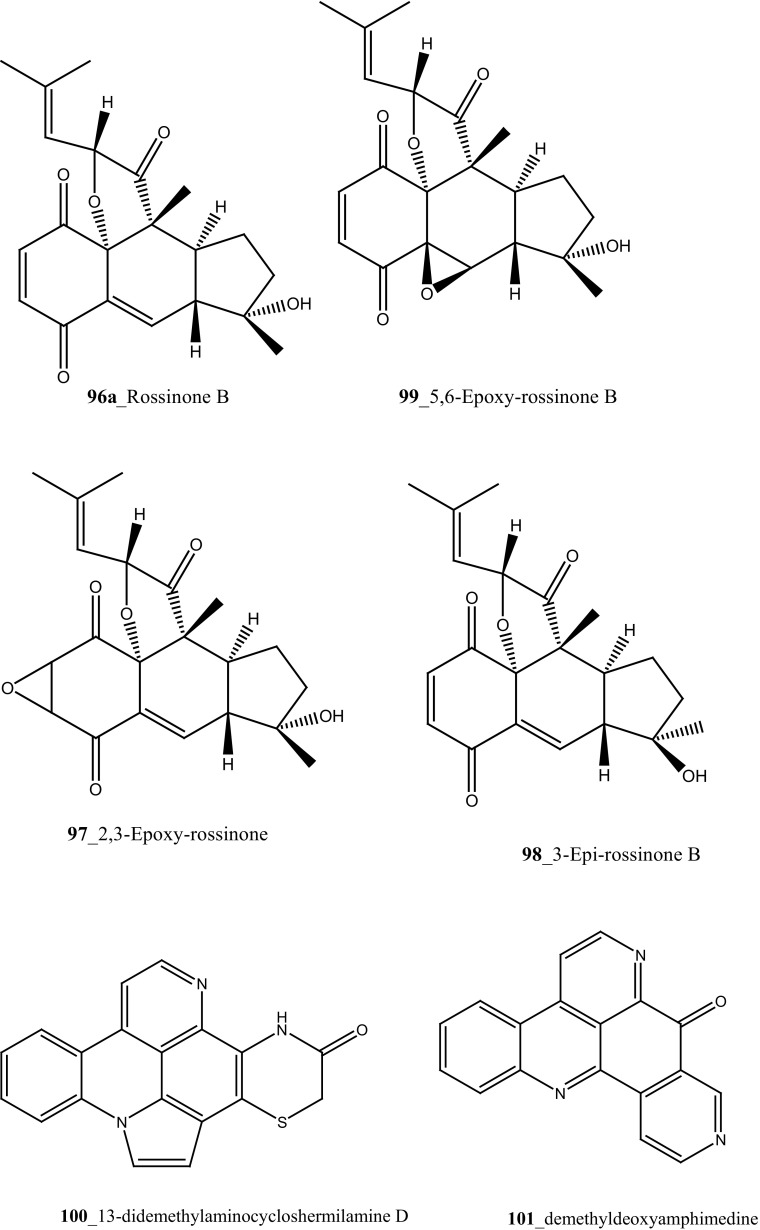

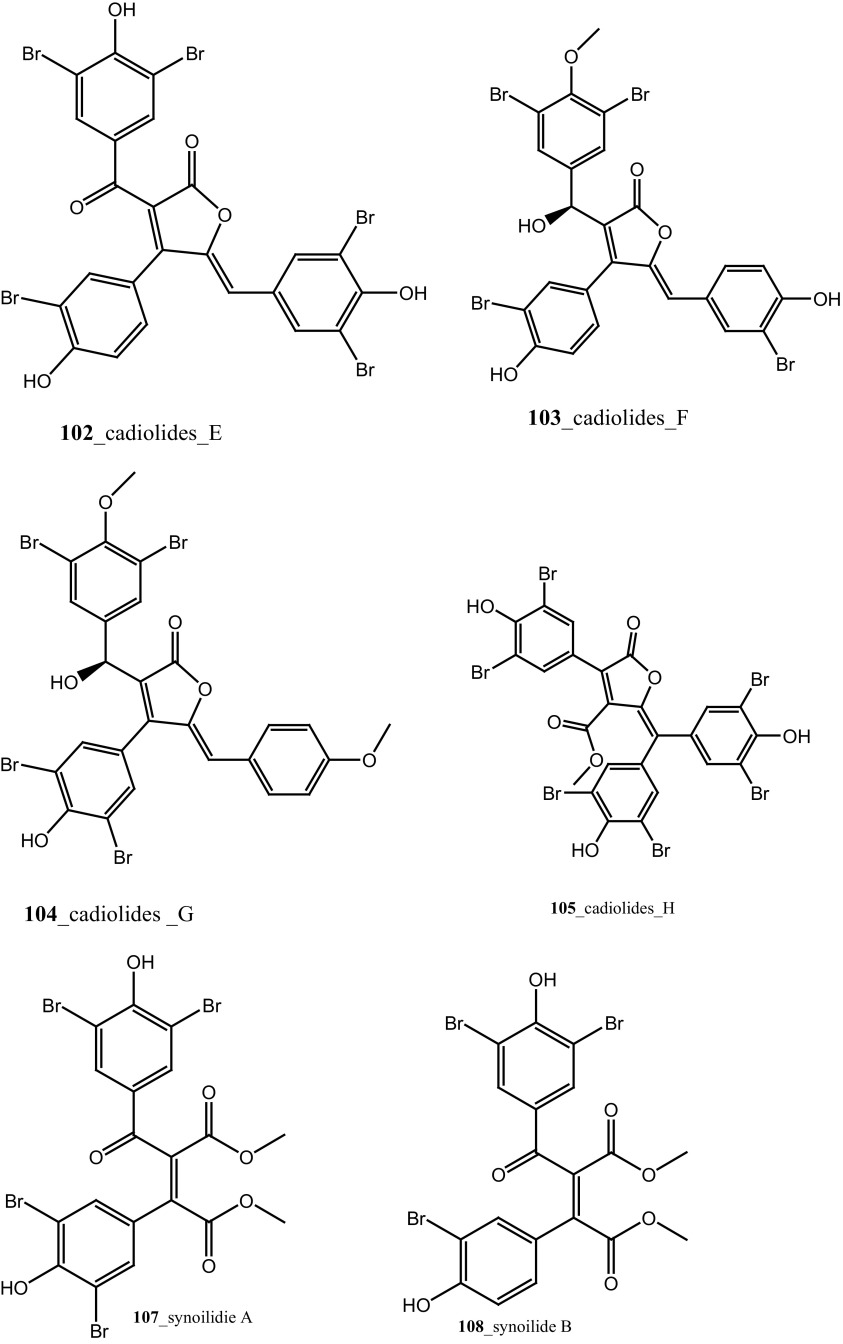

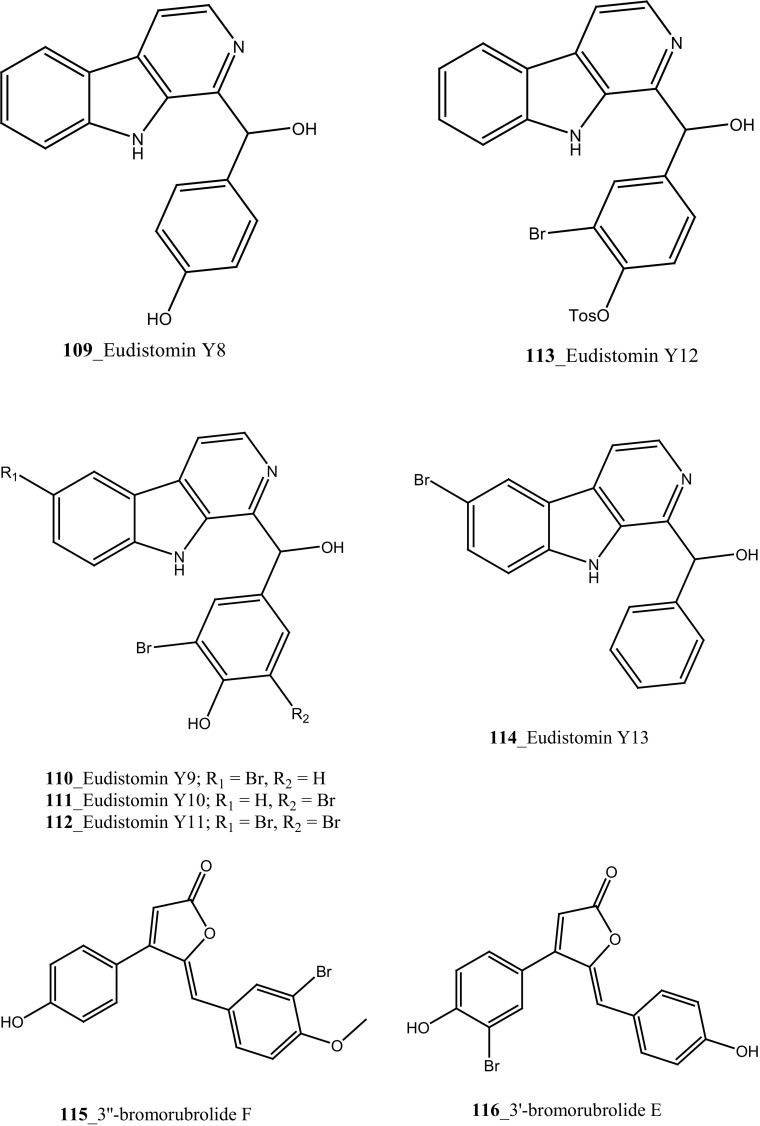

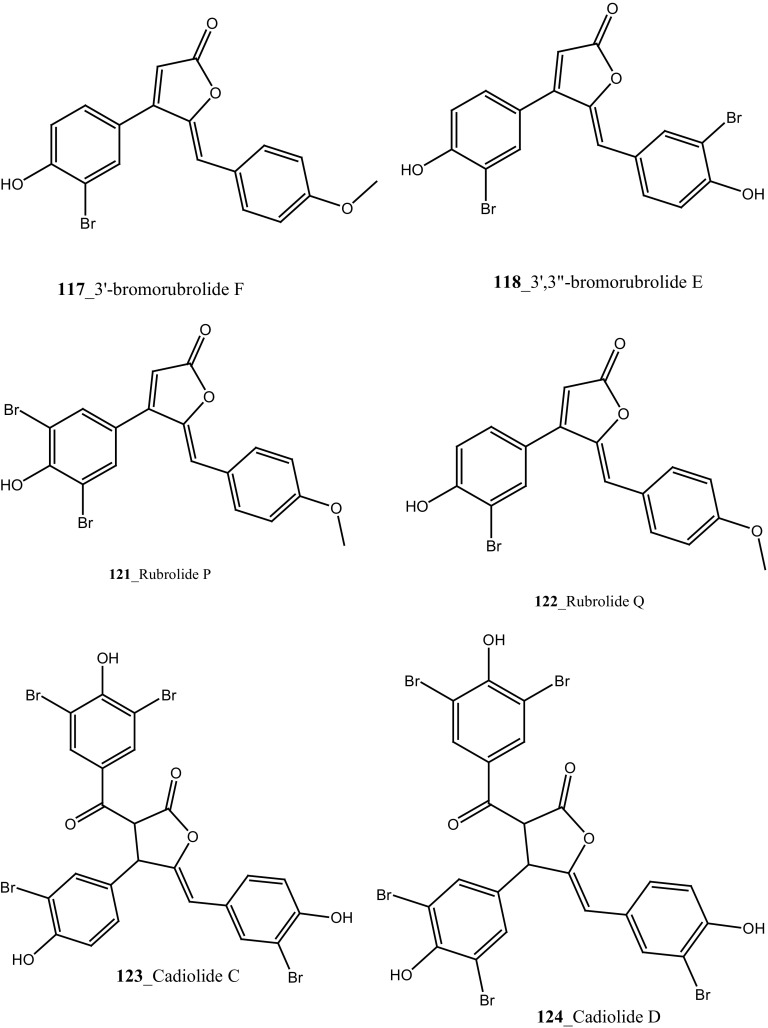

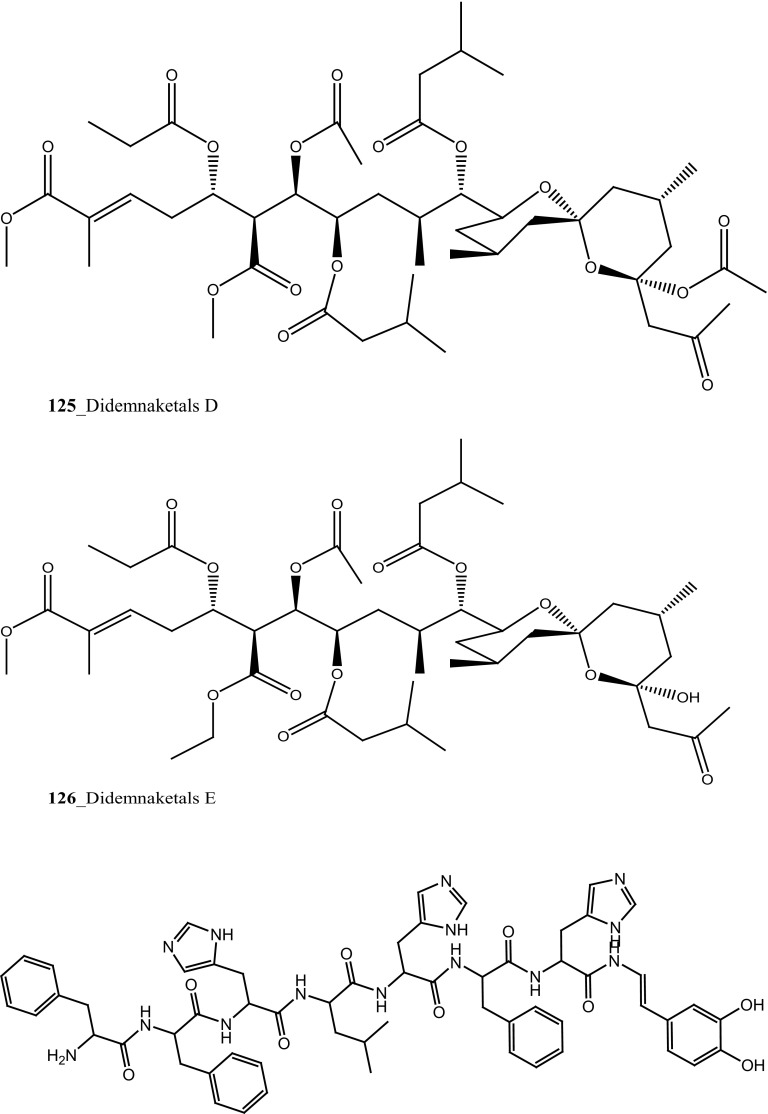

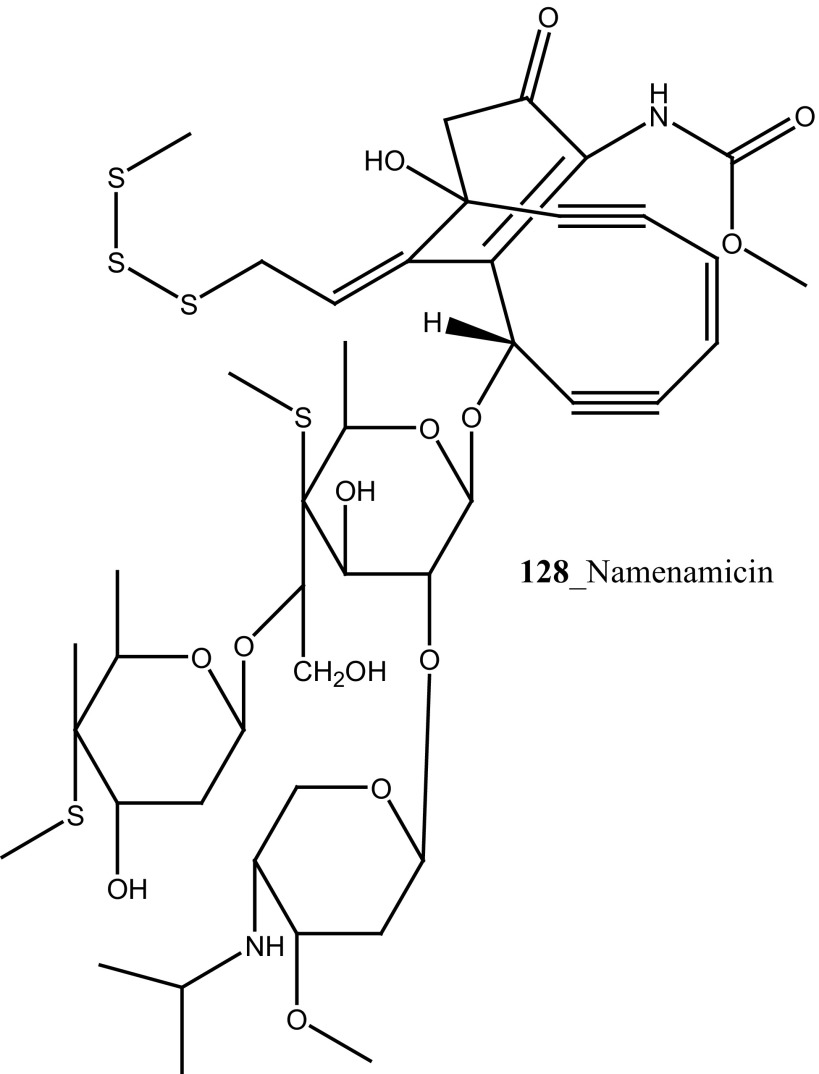
Anti-bacterial potential compounds **50**–**128**

#### Anti-tuberculosis Activity

A series of pyrido [2,3,4-kl] acridin-6-one pyridoacridine alkaloid related to ascididemin were reported from the ascidian *Lissoclinum notti* collected in New Zealand coast [76]. Metabolites, *N*-(2-(6-oxo-6*H*-pyrido[2,3,4-kl] acridin-4-ylamino)ethyl)pyrazine-2-carboxamide (**129**) and 2-(6-oxo-6*H*-pyrido[2,3,4-kl]acridin-4-ylamino)ethyl pyrazine-2-carboxylate (**130**) inhibited the growth MIC of *Mycobacterium tuberculosis* H_37_Rv (2 µM) and also showed cytotoxicity against Vero and P388 cells (>25 µM). The above study confirmed that ascididemin is a promising source for developing new anti-TB drugs in future (Structure 3).

**Structure 3:**
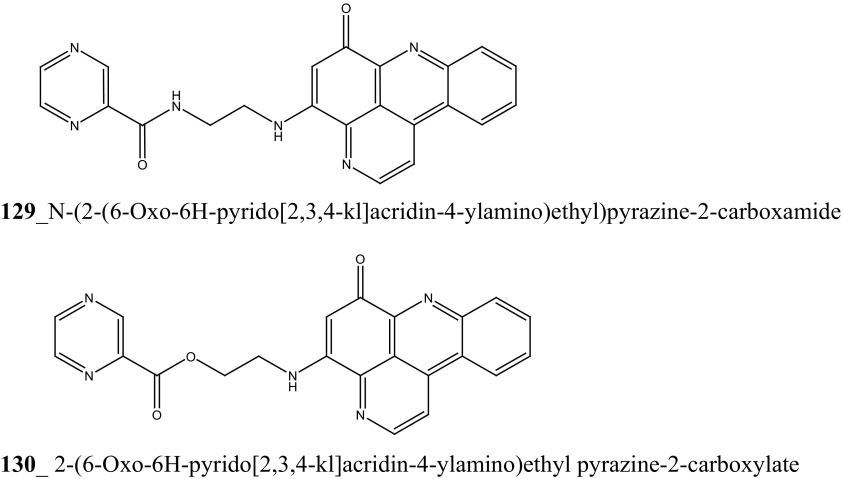
Anti-tuberculosis potentail compound (**129**–**130**)

#### Anti-fungal Activity

Anti-fungal active compound (2*S*,3*R*)-2-Aminododecan-3-ol (**131**) was isolated from the ascidian *Clavelina oblonga* collected in Brazil [77]. Compound **(131)** showed remarkable MICs against *Candida albicans* (0.7 µg/mL) and *C. glabrata* (30 µg/mL). Moreover, 2-amino-alkanols and their unsaturated derivatives have been repeatedly reported in marine ascidians and sponges [78]. Furthermore, new metabolites of cadiolides A, B (**132**–**133**) were isolated from the ascidian *Botryllus* sp. [79]. Additionally, Won et al. [68] reported cadiolides E–H (**102**–**105**) from another ascidian *Synoicum* sp. Both compounds (**102, 105**) showed a potent anti-fungal activity zone growth inhibitory with *C. albicans* (IC_50_ values of 7.62, 10.36 µM) [80]. Searle and Molinski [81] isolated the anti-fungal 2-amino alcohol (**134**) and their derivatives (**134a, b**) from the Australian *Didemnum* sp. Compound (**134)** was related to sphingosine (**135**), a widely distributed amphiphilic amino alcohol. A synthetic peptide halocidin was isolated from *Halocynthia aurantium* and showed remarkable antifungal activity against *C. albicans* (1–4 µg/mL). Hence, halocidin peptide was considered as a potential source for the development of new antibiotic resistant mechanisms [82].

#### Anti-viral Activity

A new furanone metabolite of rubrolides A, N (**136**–**137**), prunolide A (**138**) and known metabolite cadiolide B (**133)** were reported from the ascidian *Synoicum* sp. collected at Visakhapatnam coast, India [83]. Compounds (cadiolide B **133)**, and prunolide A (**138)** showed **a** significant anti-viral activity against potent RNA virus Japanese encephalitis virus (JEV) at concentrations of 1 µg/mL. Furthermore, four new nucleoside derivatives (**139**–**142**) were reported from the ascidian *Herdmania momus* collected at coast of Jeju Island, Korea [84]. Rare nucleosides (**139**–**142**) have been tested the anti-viral activity against various human pathogenic viruses. Though, none of the isomers exhibited significant inhibition against human rhinoviruses (HRV14 EC_50_ > 100 µg/mL, CC_50_ > 100 µg/mL), poliovirus (PV3, EC_50_ > 100 µg/mL, CC_50_ > 100 µg/mL), and coxsackieviruses (CoxB1 or CoxB3, EC_50_ > 100 µg/mL, CC_50_ > 100 µg/mL).

#### Anti-HIV Activity

An unusual sulfated mannose, homopolysaccharide (**143**) also known as kakelokelose, was reported from the mucous secretion of the ascidian *D. molle* collected from Pohnpei, Micronesia and Manoda, Indonesia [85]. Compound (**143**) showed a remarkable anti-HIV activity determined 100% potential to inhibit infection with CEM cells by HIV strain RF at 0.3 µg/mL, whereas no cytotoxicity against CEM cells at concentrations 15 µg/mL. Furthermore, five lamellarins, the 20-sulfates of lamellarins B, C, L, lamellarin G 8-sulfate and lamellarin Z (**144**–**148**) were isolated from *Didemnum chartaceum* from the Great Barrier Reef, Australia. Unusually long relaxation times were observed for certain signals in their ^**1**^H NMR spectra [86]. During further investigation, Reddy et al. [87] reported lamellarin α 20-sulfate (**149**) from an unidentified ascidian, collected in Indian waters. Compound (**149**) showed moderate inhibition of anti-HIV activity against HIV-1 protease (IC_50_ 16 µM).

An anti-retro viral metabolite, cyclodidemniserinol trisulfate (**150**) was reported from the ascidian *Didemnum guttatum* collected at Ngerchaol Island, Palau. Compound (**150**) is very similar to didemniserinolipid A (**151**), reported from an Indonesian *Didemnum* sp. [88], though few significant variations between these two chemical structures, like the presence of an additional ring containing a glycine unit and the presence of sulfate groups, were observed. Moreover, Gonzalez et al. [88] also reported didemniserinolipids B and C (**152**–**153**) from the same ascidian species. Anti–HIV activity of compound (**150**) showed a modest inhibition against HIV-1 protease (IC_50_ 60 µg/mL) and with MCV topoisomerase IC_50_ 72 µg/mL. Compound (**150**) showed no selectivity for integrase inhibition.

During the screening of anti-HIV agents from marine organisms, Donia et al. [89] reported two new cyclic hexapeptide alkaloid including mollamides B (**154**) and C (**155**), with known peptide keenamide A (**156**) from the tunicate *Didemnum molle* collected in Manado Bay, Indonesia. Compound (**154**) showed in vitro anti–HIV activity against HIV-1 human PBM cells (EC_50_ 48.7 µM). Lu et al. [90] isolated two new Thiazoline peptides, mollamides E and F (**157**–**158**) and one new a Tris-Phenethyl urea, molleurea A (**159**) from the same ascidian *D. molle* collected in Papua New Guinea. Compounds (**157**–**159**) were tested for anti-HIV activity in both an HIV integrase inhibition assay and a cytoprotective cell-based assay. Mollamide F (**158**) showed a moderate activity against both bio-assays with IC_50_ values of 39 and 78 µM, and molleurea A (**159**) was effective in the cytoprotective cell-based assay (IC_50_ 60 µM) (Structure 4).

**Structure 4:**
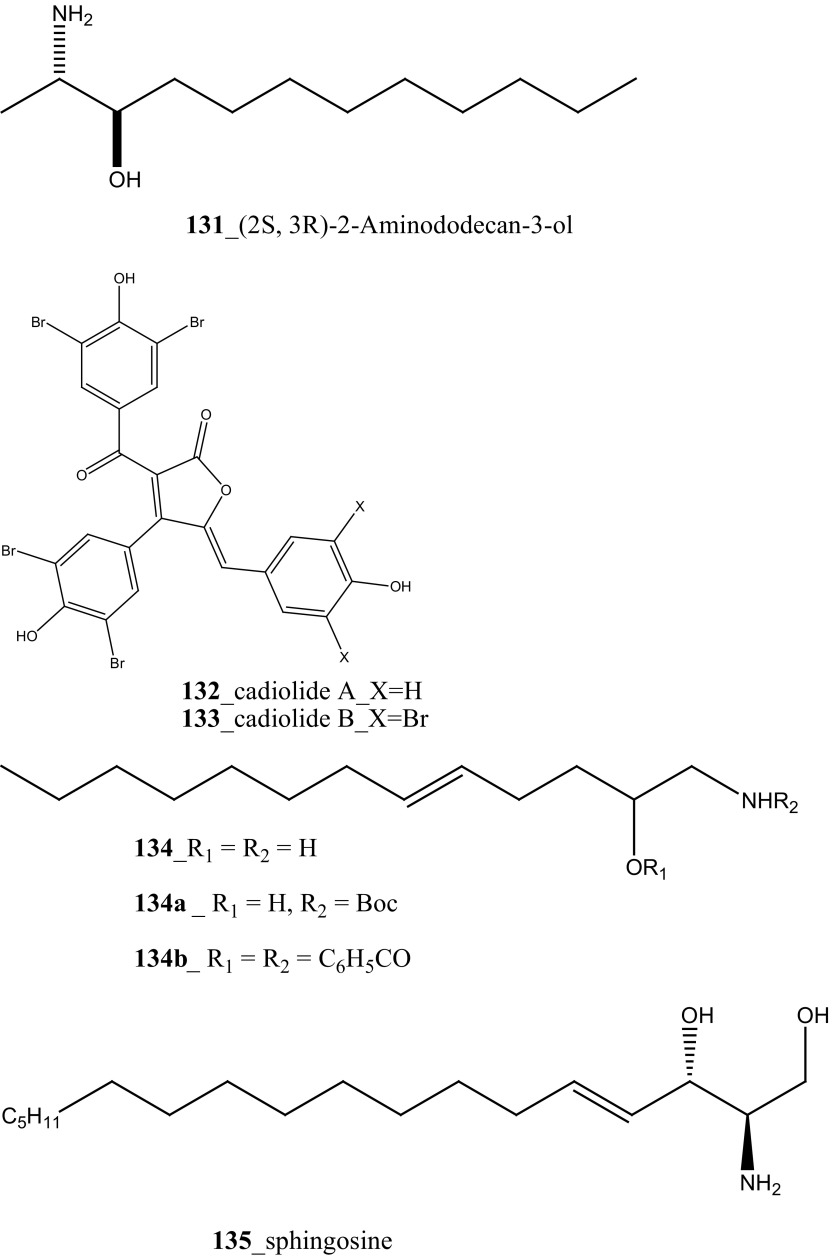

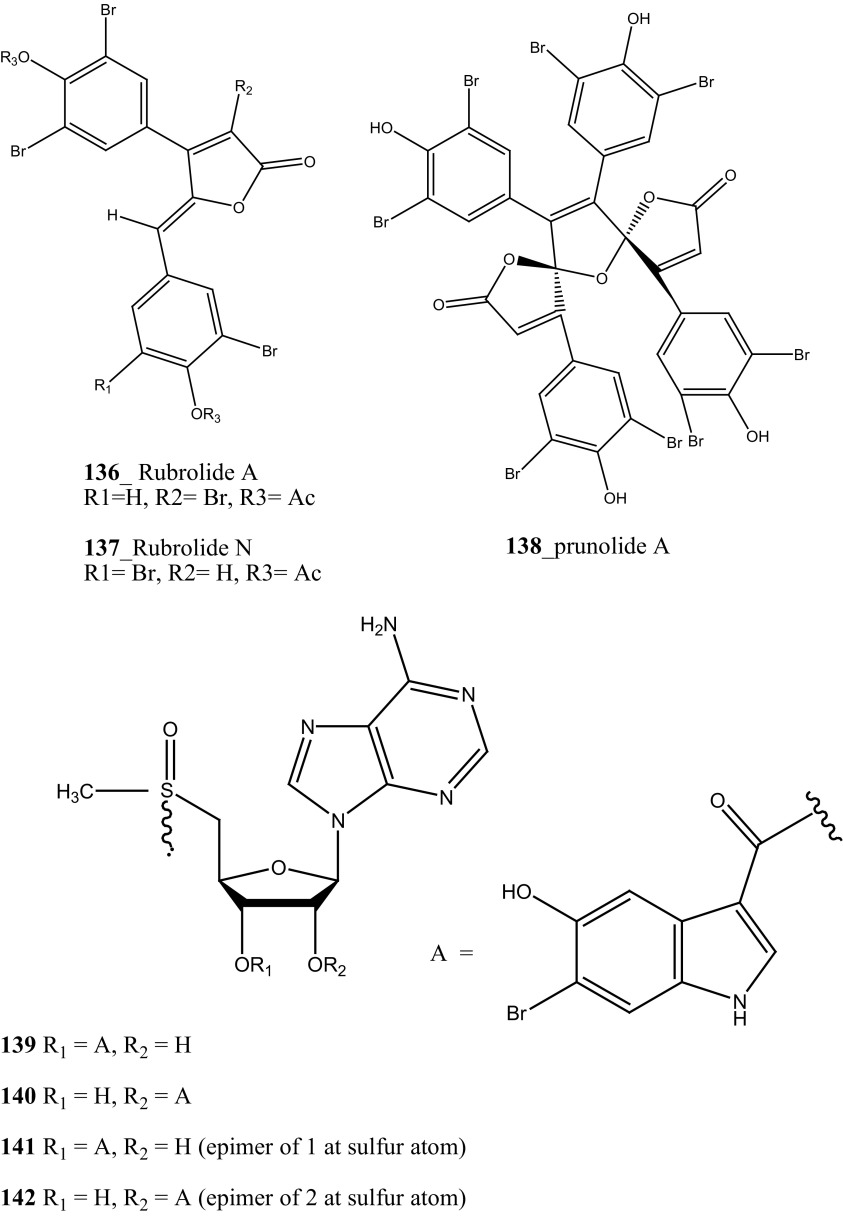

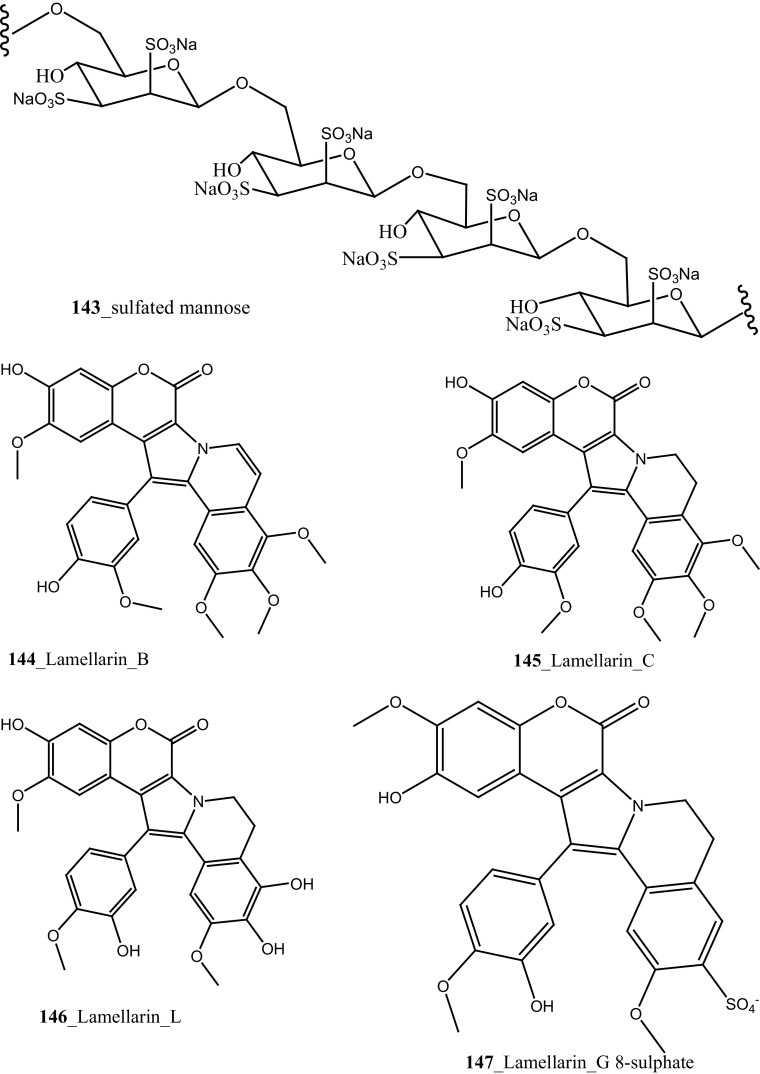

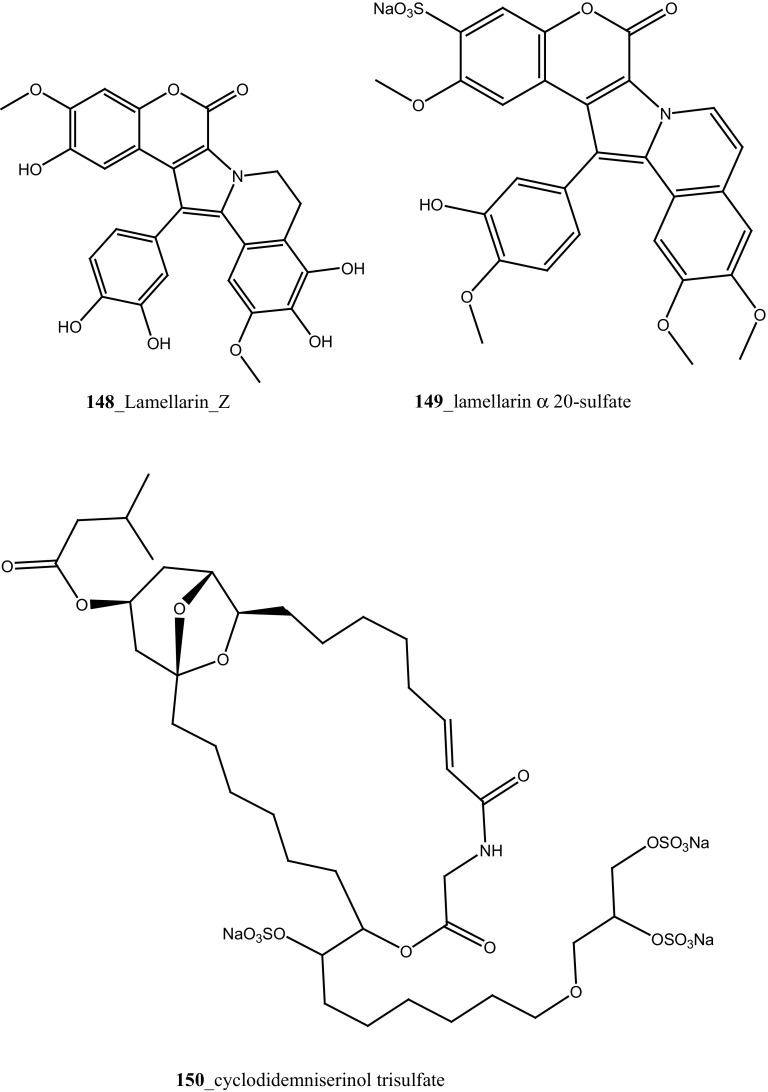

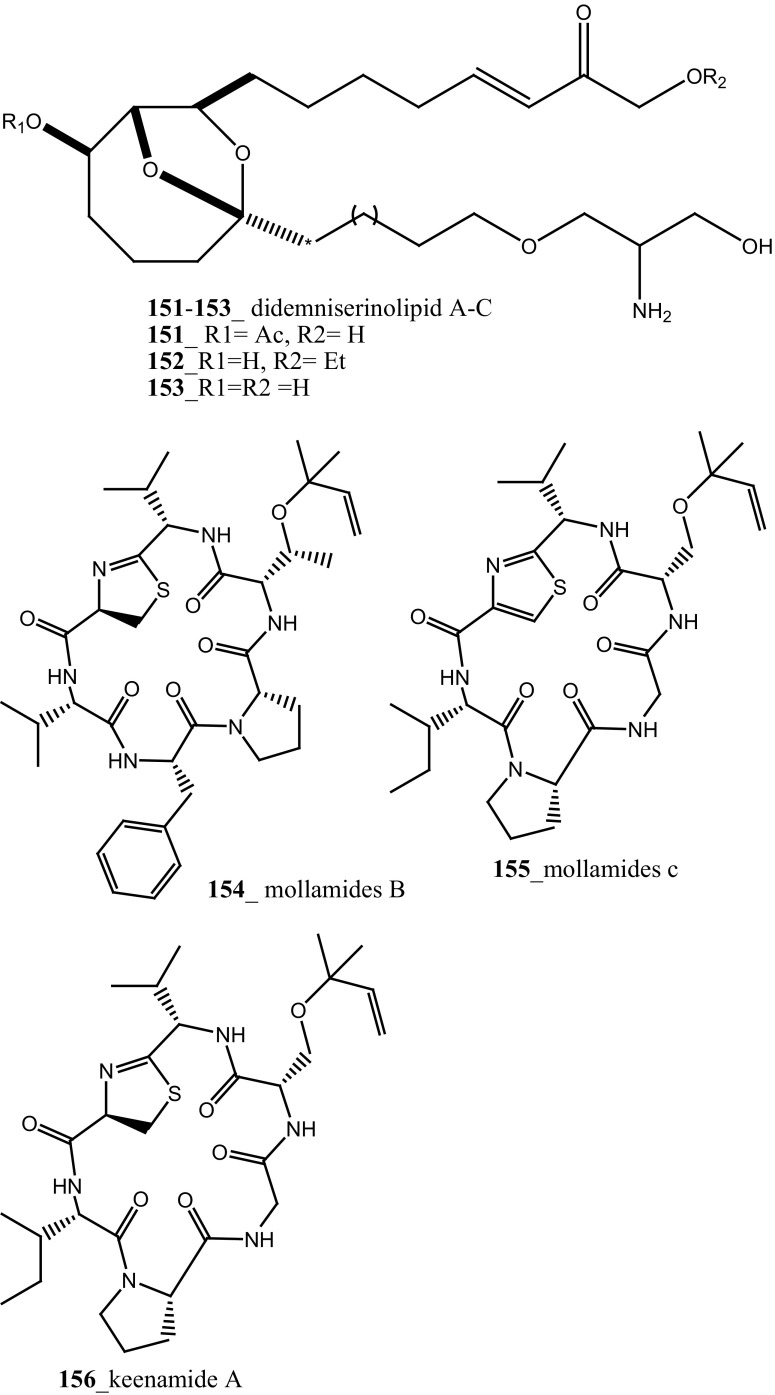

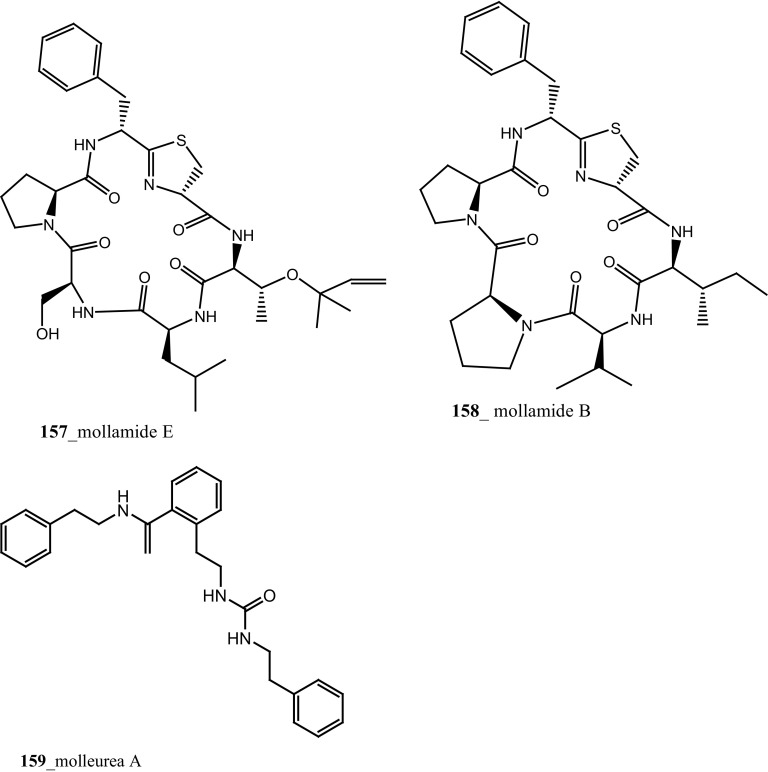
Anti-fungal potential compounds (**131**–**159**)

### Anti-protozoal Activity

#### Anti-malarial Activity

The recent research outcome points out that ascidians may produce secondary metabolites with potential activity against malarial parasites. Mendiola et al. [91] reported anti-malarial activity of three ascidian species, *Microcosmus goanus*, *Ascidia sydneiensis* and *Phallusia nigra* collected at north-west Cuban coast. The crude compounds extracted by water and n-butanol showed 50% inhibition against *P. falciparum* growth at concentrations of (17.5, 20.9, 29.4 µg/mL), respectively. Meridianin is a small family of alkaloids, meridianins F, G (**94, 95**) were reported from the ascidian *Aplidium meridianum* [62]. Compound (**85**) was synthesized by Lebar et al. [92] and further investigation of meridianins A–G (**95**) which was physically synthesized by catalytic Domino amino reaction [93]. Compounds **85, 95,** tested to anti-malarial inhibition against human malaria parasite *P. falciparum,* showed a moderate activity against both strains IC_50_ 50 and 4.4 µM [92, 94]. Furthermore, Donia et al. [89] reported the anti-malarial activity of Mollamide B (**154**), showed modest activity against *P. falciparum* (D6 clone and W2 clone) with (IC_50_ 2.0, 2.1 µg/mL). Compound **154** showed an activity against *Leishmania donovani*, with IC_50_ (18 µg/mL) and IC_90_ 35 μg/mL, respectively. The new brominated indole alkaloid, kingamide A (**160**) have isolated from the ascidian *Leptoclinides kingi* collected off Hook and Hardy Reefs in Australia [95]. No anti-malarial activity was observed against kingamide A at concentration 10 µM.

New anti-malarial meroterpenoids, 2-geranyl-6-methoxy-1,4-hydroquinone-4-sulfate (**161**) and scabellones A–D (**162**–**165**) a family of pseudodimeric meroterpenoids were isolated from the ascidian *Aplidium scabellum* collected off Rabbit Island, New Zealand [96]. Remarkably, compounds **162**–**165** have rare benzo [*c*] chromene-7,10-dione scaffold among the MNPs. Compound (**163**) showed moderate potential activity against *P. falciparum* (K1 chloroquine-resistant strain) with IC_50_ 4.8 µM and also showed weak cytotoxicity with *Leishmania donovani* (25 µM), *T. brucei rhodesiense* (28 µM) and *Trypanosoma cruzi* (49 µM) respectively. The preliminary result of the anti-malarial activity of scabellone B (163) showed no noticeable apoptosis against human neutrophils. It is recommended that this drug like pseudodimeric meroterpenoid (scabellone B) may be utilized for developing new drug class for new treatments of malarial disease. Additionally, two new indole spermidine alkaloids, didemnidines A, B (**166**–**167**) reported from the ascidian *Didemnum* sp. collected from Tiwai Point, New Zealand [97]. Didemnidine B (**167**) showed modest in vitro growth inhibition against *P. falciparum* (IC_50_ 8.4 µM and *T. brucei rhodesiense* (9.9 µM). Anti-malarial tetrahydro-*β*-carbolines, (−)-7-bromohomotrypargine (**168**), and 3 alkylguanidine-substituted *β*-carbolines, opacalines A, B, and C (**169-171**), and their substitutes (+)-1*R*-7-bromotrypargine (**172**) were isolated from the ascidian *Pseudodistoma opacum* collected at Maori Bay, New Zealand [98]. The structure of compound (**168**) is similar to recently reported sponge metabolite (+)-1*R*-7-bromotrypargine (**172**), with compound **168** containing one additional methylene residue in the guanidinylated side chain (Davis et al. 2010). Remarkably, Van Wagoner et al. [99] have been reported structurally related trypargine metabolites 1-carboxytrypargine (**173**) and trypargimine (**173a**) from the ascidian *Eudistoma* sp. Compounds **(169**–**170)** showed a modest antimalarial activity against chloroquine-resistant strain of *P. falciparum* (IC_50_ 2.5, 4.5 µM) [98].

A new anthrone-anthraquinone compound, albopunctatone (**174**), and known compound 1,8-dihydroxy-9,10-anthraquinone (**175**) were isolated from the ascidian *Didemnum albopunctatum* collected in Australian Great Barrier Reef [100]. Compound (**174**) showed a potential anti-plasmodial activity against chloroquine-resistant and malarial parasite, *P. falciparum* (IC_50_ 5.3 and 4.4 µM), and compound (**175**) was inactive at concentration 40 µM, respectively. Anti-protozoal compound, dioxothiazino-quinoline-quinone ascidian metabolite orthidine F (**176**), ascidiathiazone A (**177**) and their analogues (**177 a, b**) have reported from New Zealand ascidian *Aplidium* sp. [101]. Compound (**177**) showed a moderately potent anti-protozoal activity against *P. falciparum* K1 strain (IC_50_ 3.3 µM), *Trypanosoma brucei rhodesiense* (IC_50_ 3.1 µM) and completely inactive against *T. cruzi* and *Leishmania donovani* and have shown poor cytotoxicity against a mammalian cell-line (IC_50_ 170 µM).

A polyaromatic alkaloid, aplidiopsamine A (**178**) reported from *Aplidiopsis confluata*, collected at Tasmania in Australia coast [102]. Compound (**178**) showed a potential anti-malarial activity against chloroquine sensitive (3D7) with (IC_50_ = 1.47 (3D7) and resistant (Dd2) strains of the malarial parasite, *P. falciparum* (IC_50_ = 1.65 µM). In addition, compound (**178**) showed ∼100% inhibition at the highest concentration (120 µM) against normal cell line HEK-293. Anti-malarial compounds of cyclic peptides such as ulithiacyclamides A, B (**179, 180**) and patellamides A, B C, (**181**–**183**), patellamide D (**184**), patellamides E–G (**185**–**187**) and ascidiacyclamide (**188**) were reported [103–107]. Furthermore, Morris et al. [108] reported the metal binding selectivity of compounds (**179, 181, 183**) by circular dichroism spectroscopy. The result of this study represents that Cu^2+^ is a biologically relevant metal for cyclic peptides patellamide C, ulithiacyclamide A, whereas compound (**181**) showed less significant cytotoxicity (Structure 5).

**Structure 5:**
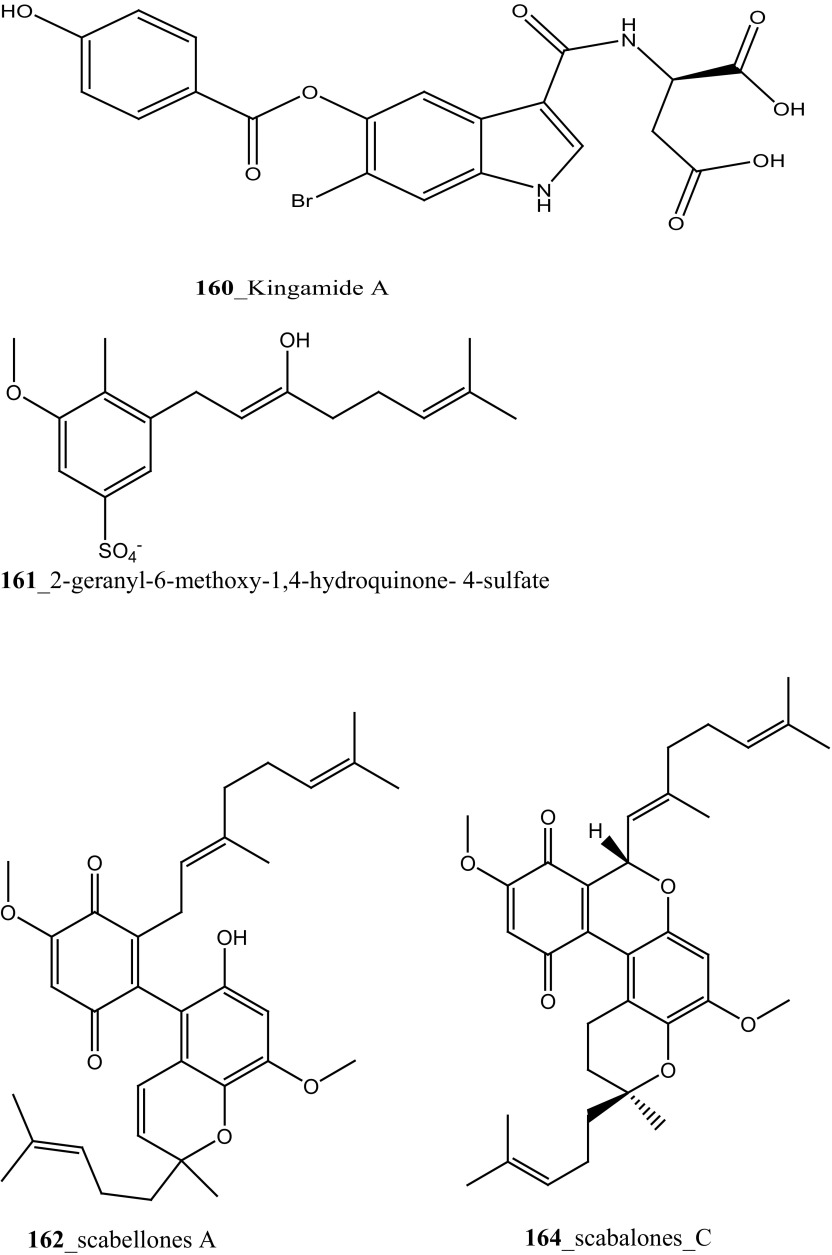

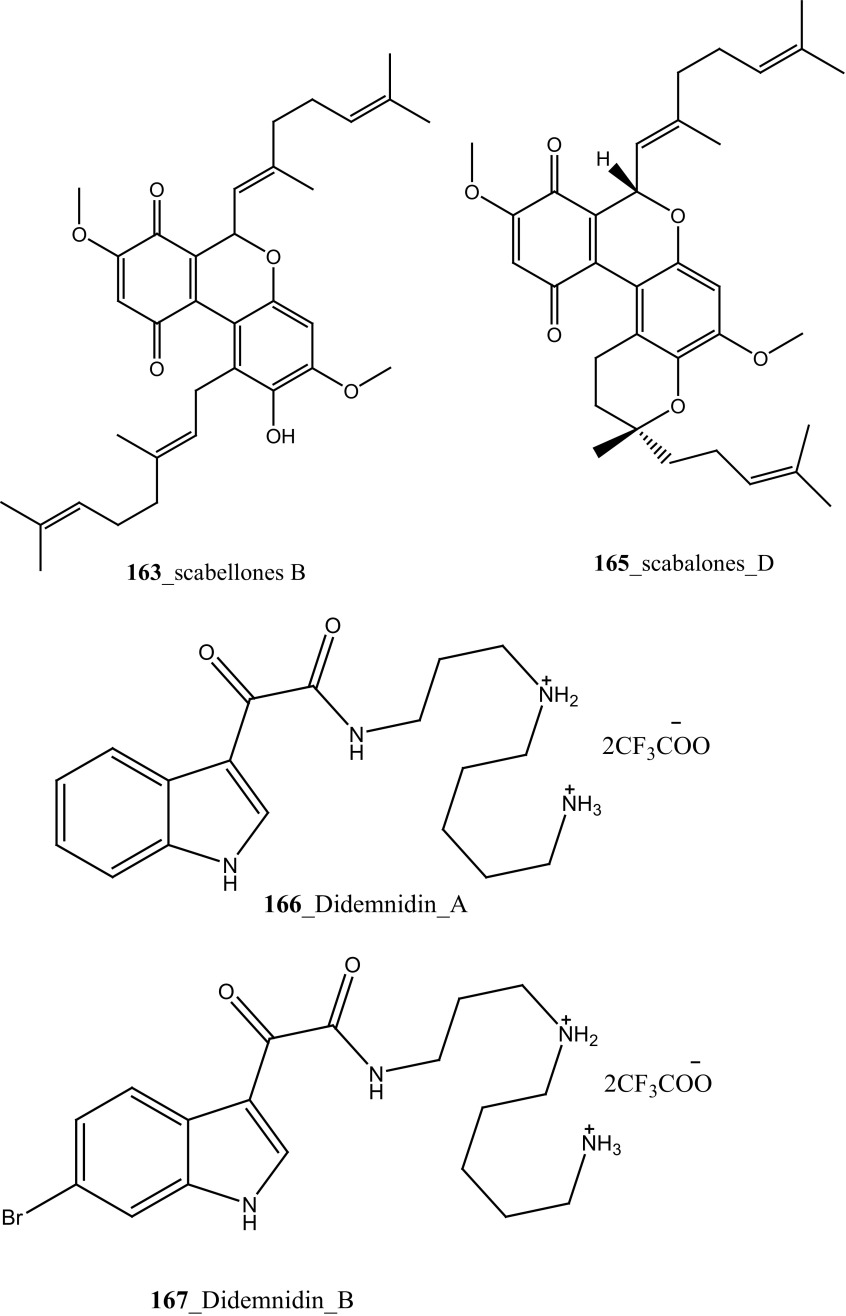

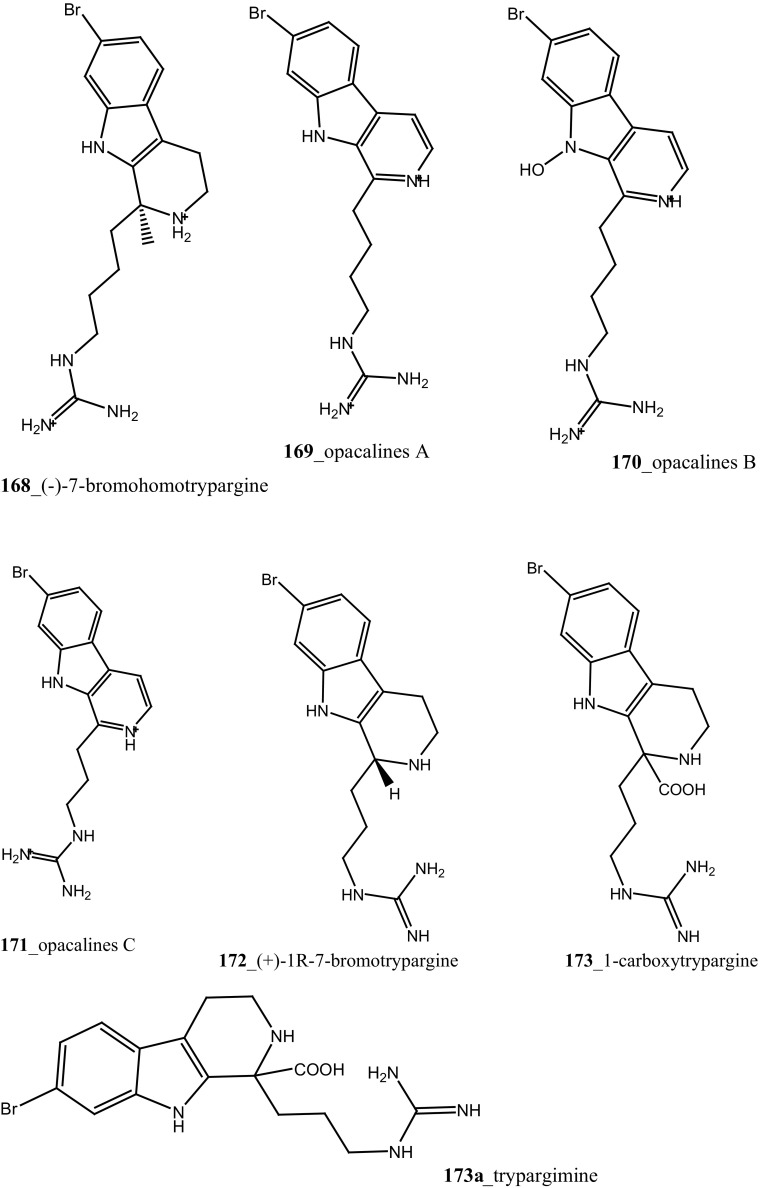

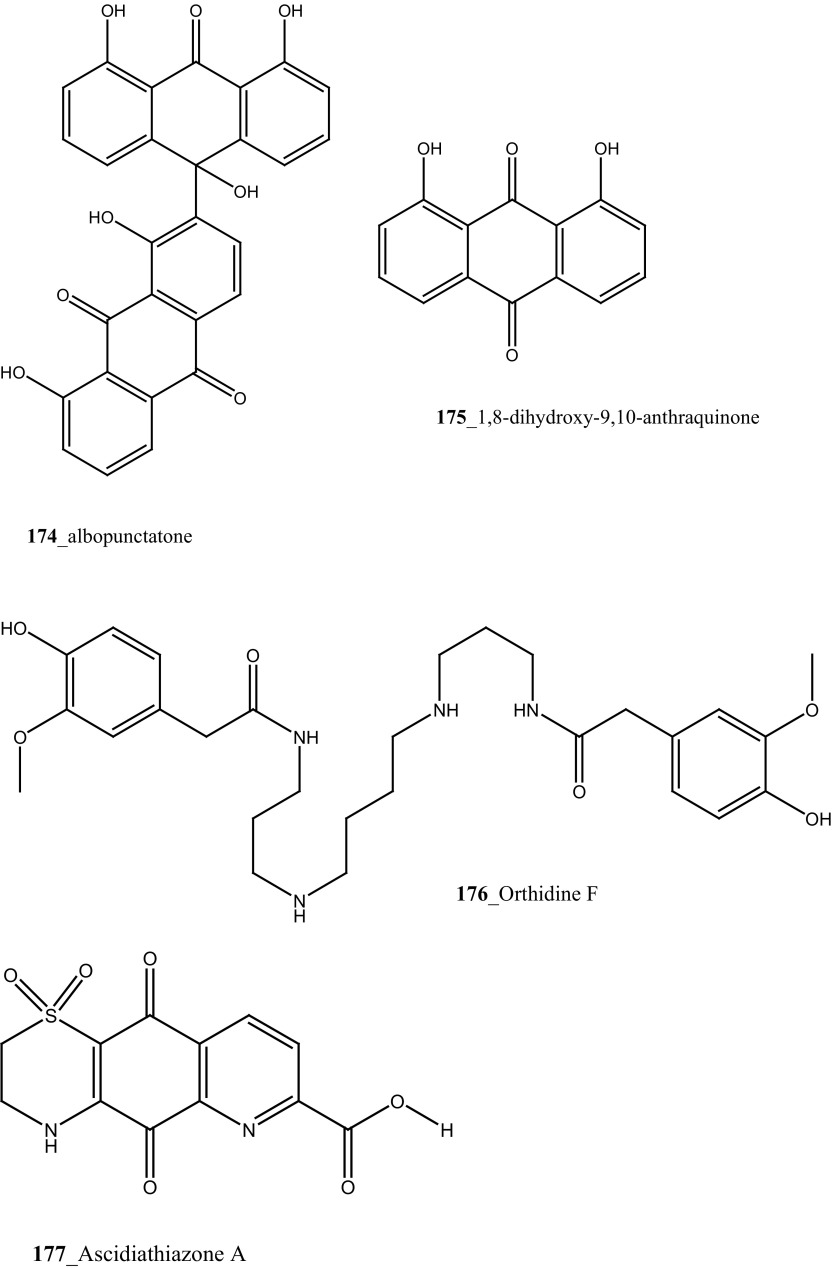

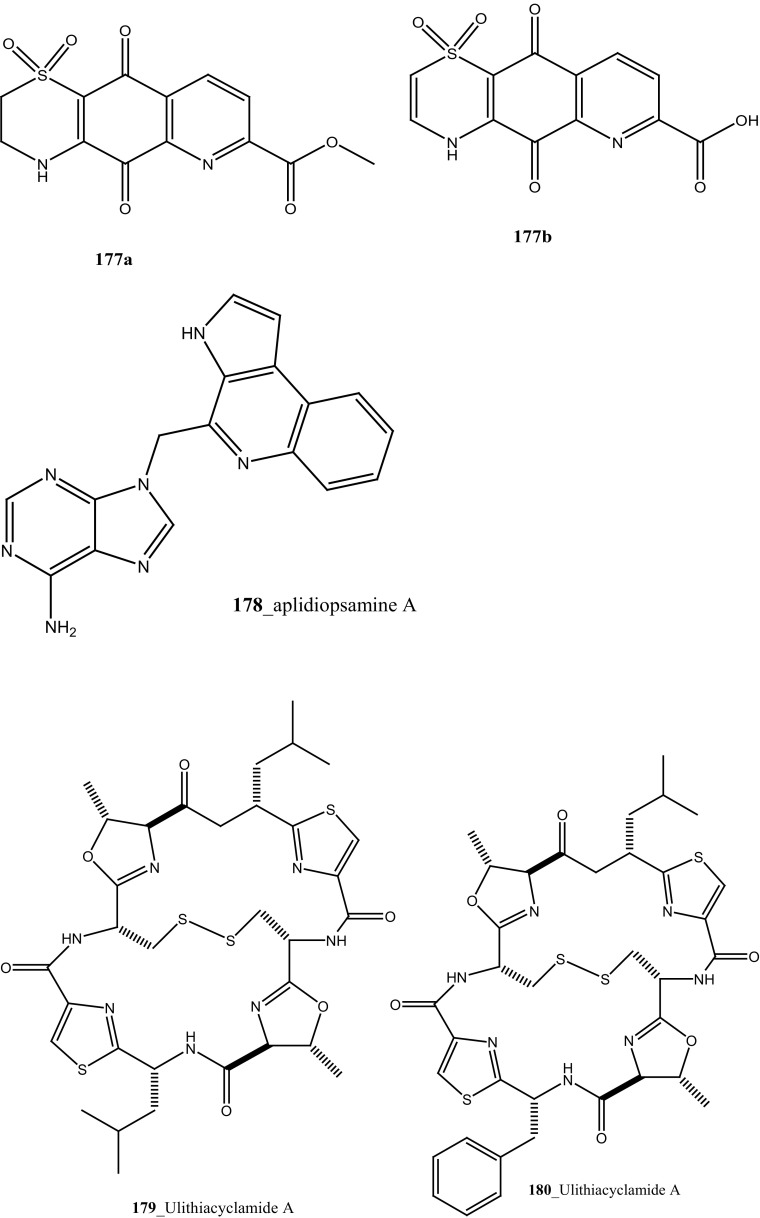
Anti-malarial potential compounds (**84**, **94**, **95**, **160**–**188**)

#### Anti-trypanosomal Activity

New pyridoacridine alkaloids, 12-deoxyascididemin (**189**), and two well-known analogues, ascididemin (**5**) and eilatin (**190**) were isolated from the ascidian *Polysyncraton echinatum* collected in North West of Farquharson Reef, Australian coast [109]. Compounds (**189, 5, 190**) had shown potent anti-trypanosomal activity against *Trypanosoma brucei brucei* (IC_50_ 0.077, 0.032, 1.33 µM). Additionally, compounds (**189, 5)** showed a moderate cytotoxicity against HEK293 cell line (IC_50_ 7.63, 1.48 µM). Simarro and co-workers had reported 70 million populations of 37 sub-saharan African countries were affected deadly infectious disease [110], hence it is urgently required to develop new effective drugs to fight against African sleeping sickness (Structure 6).

**Structure 6:**
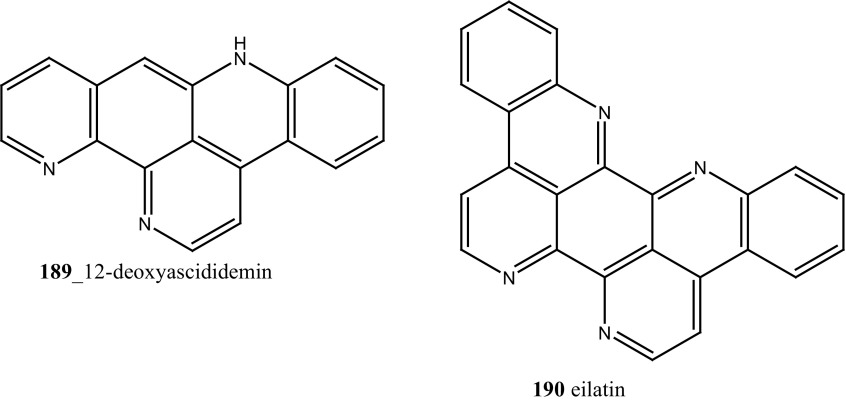
Anti-trypanosomal potential compounds (**5**, **189**, **190**)

### Anti-diabetic Activity and Anti-oxidant Activity

Four different group of MNPs reported from the ascidian species were found to inhibit human aldose reductase. From the red ascidians *Botryllus leachi,* compounds triphenylpyrrolo-oxazinones, lukianols A, B (**191 a, b**) and known alkaloids (**192, 193a, b**) were reported [111, 112]. All these compounds hold the heterocyclic system and no less than two phenolic groups are present in the chemical structures [113]. Compounds, imidazole derivative (**191)** and pyrazine derivatives (**193a, 193b)** contain a common central nitrogenous heterocycle core. Compound lukianol B (**191**) showed a strong growth inhibitory (0.6–0.8 µM) against aldose reductase inhibitor (ARI) compared to other phenolic derivatives. Further, compounds (**192, 193a, b)** have shown moderate inhibitory activity against human aldose reductase (h-ALR2) with IC_50_ 21.4, 41.4, 19.4 µM.

Novel *β*-carboline guanidine alkaloid, tiruchanduramine (**194**) were reported from *Synoicum macroglossum* collected in Tiruchandur coast, India [114]. Compound (**194**) exhibited a moderate α-glucosidase inhibitory activity (IC_50_ 78.2 µg/mL). Compound (194) is the first MNPs containing enduracididinamine, the decarboxylation product of enduracididine, a rare amino acid was obtained by hydrolysis of enduracidin from *Streptomyces fungicidicus* [115]. The absolute stereochemistry of this compound is not yet established. Krishnaiah et al. [116] have reported lamellarins *γ*, α and *ɛ* **(195**–**197)** and known lamellarin alkaloids, lamellarins M, K, K-diacetate, K-triacetate, lamellarins U, I, C-diacetate, and X-triacetates (**198**–**205**) from *Didemnum obscurum* collected at Tiruchandur coast, India. Compounds, lamellarins *γ* and K-triacetate (**195**, **201**) showed a more potent activity (3.28, 2.96 mM) than other biomolecules (Structure 7).

**Structure 7:**
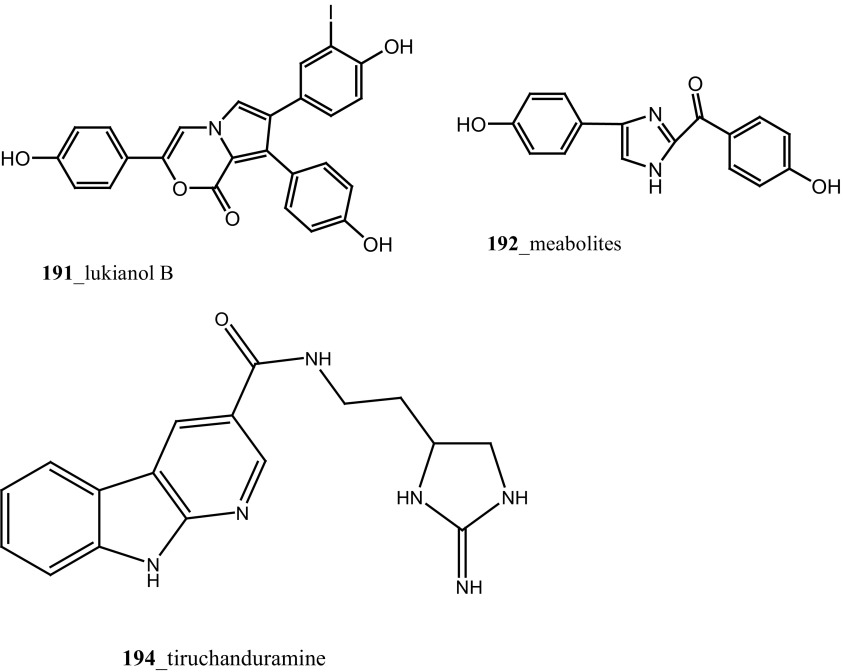

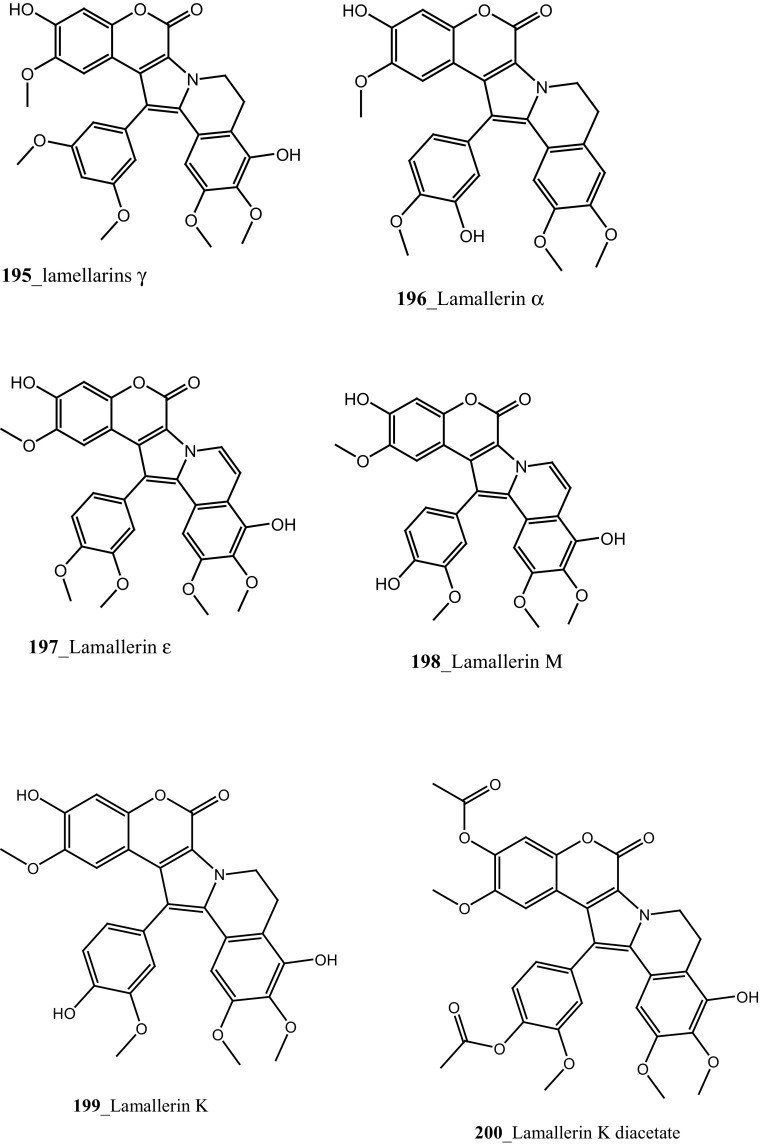

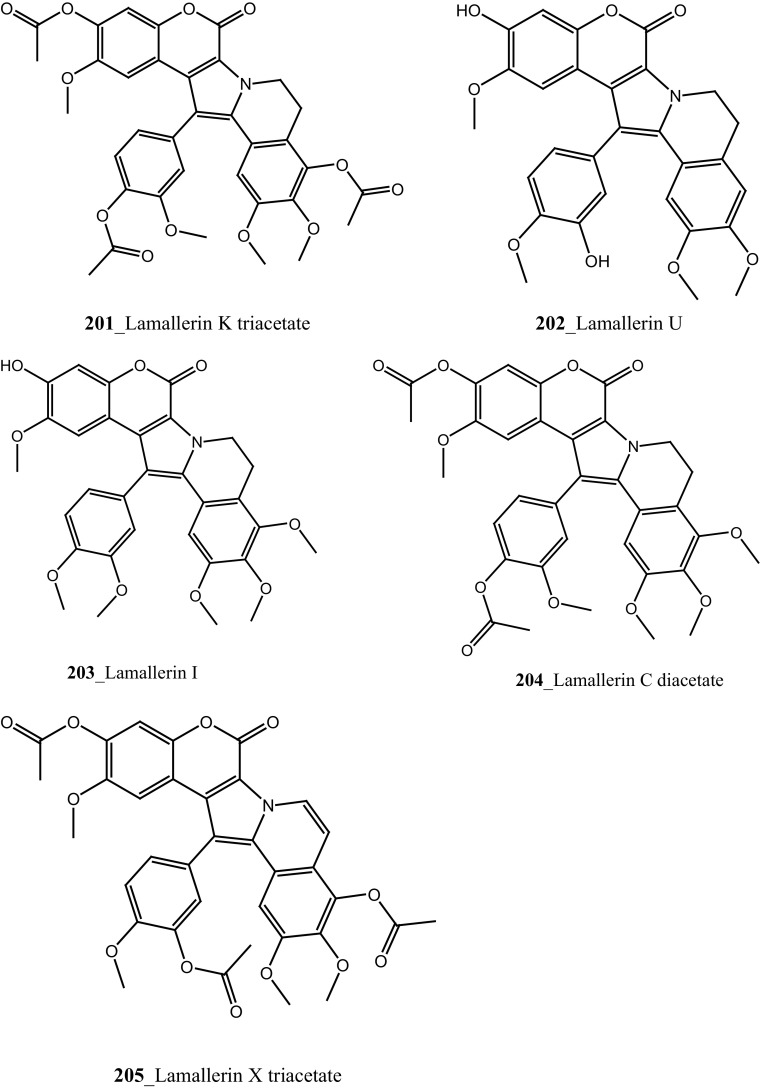
Anti-diabetic and anti-oxidant potential compounds (**191**–**205**)

### Anti-inflammatory Activity

Two new tricyclic thiazine-containing quinolinequinone alkaloids, ascidiathiazones A and B (**177**, **206**) were reported from *Aplidium* sp. collected in New Zealand ascidian coast. Compounds (**177**, **206**) showed a potential inhibition against human neutrophils (IC_50_ 1.55, 0.44 µM), respectively [117]. Furthermore, rubrolide O (**207**) was reported from the *Synoicum* sp. collected in New Zealand coast. Rubrolide O is a new member of rubrolide family exist a mixture of *E/Z*-isomers. Compound (**207**) showed a modest inhibition against human neutrophil free radical release (IC_50_ 35 µM) [118]. Belmiro and co-workers reported anti-inflammatory properties of a dermatan sulfate similar to mammalian heparin from the ascidian *Styela plicata*, collected in Brazil coast [119, 120]. The molecular characterization of this analogue have been tested by in vivo rat colitis model, showing a significantly decreased lymphocyte and macrophage recruitment as well as TNF-*α*, TGF-*β*, and VEGF production in the inflamed rat colon at concentration 8 mg/kg per day.

New anti-inflammatory meroterpenoids, 2-geranyl-6-methoxy-1,4-hydroquinone-4-sulfate (**161**) and scabellone B **(163)** showed a moderate potent anti-inflammatory activity (21, 125 µM), and selective ability to inhibit neutrophil respiratory burst proves that meroterpenoid sulfates may have potential for developing novel marine drugs for treatment of inflammation [96]. Biologically active amino acid derivatives, herdmanines A–D (**208**–**211**) were reported from the ascidian *Herdmania momus*, the unusual D-form of arginine present in herdmanines A–C [121]. Both compounds (**210, 211**) showed adequate suppressive effects on the production of NO (IC_50_ 96 and 9 µM) and these compounds have the potential to inhibit the mRNA expression of iNOS. Additionally, compound **211** exhibited a strong inhibition of mRNA expression of pro-inflammatory cytokines IL-6. Further investigations are needed to search for potential anti-inflammatory MNP from the same ascidian. A series of anti-diabetic amino acid derivatives new congeners herdmanines E–L (**212**–**220**) and (−)-(*R*)-leptoclinidamine B were reported [122, 123]. Indoleglyoxylyl derivatives, herdmanine K showed strong PPAR-*γ* activation in rat liver cells (Ac2F) 1 and 10 µg/mL in a cell-based luciferase reporter assay. Anti-inflammatory activity of methanol crude compounds extracted from the ascidian *Eudistoma virde* was exhibited modest anti-inflammatory activity at various concentration up to 200 mg/kg compare to the positive control Diclofenac [124]. The biological activity is similar to that one of other ascidians *Synoicum* sp. and *Pycnoclavella kottae* [48, 118] (Structure 8).

**Structure 8:**
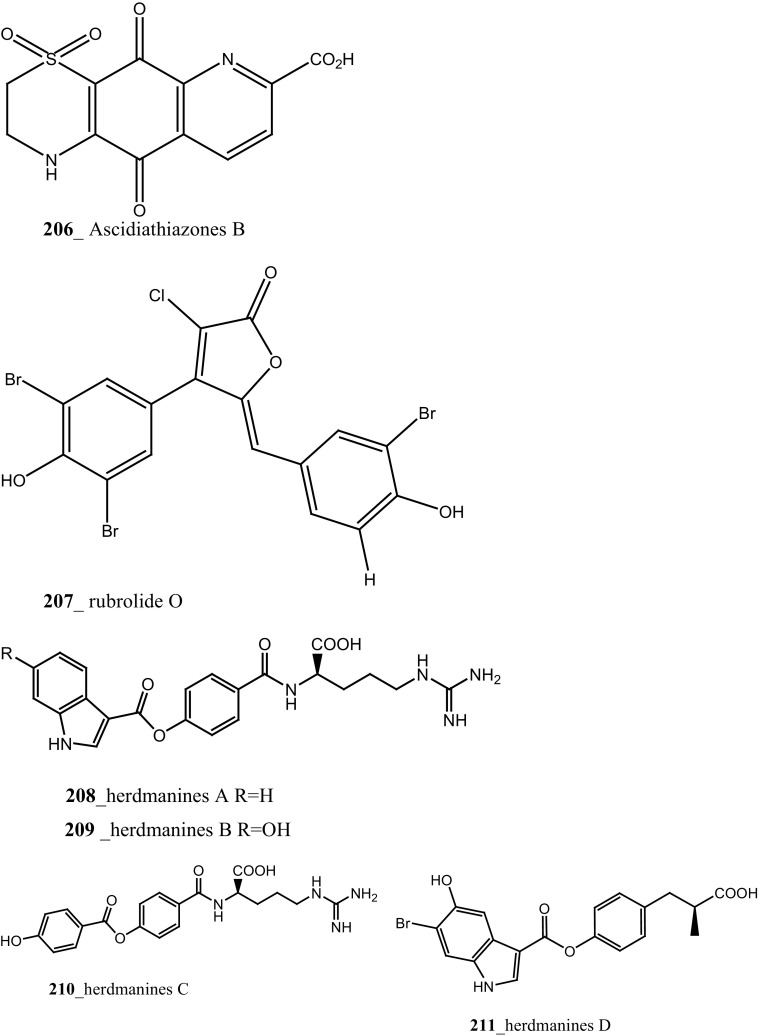

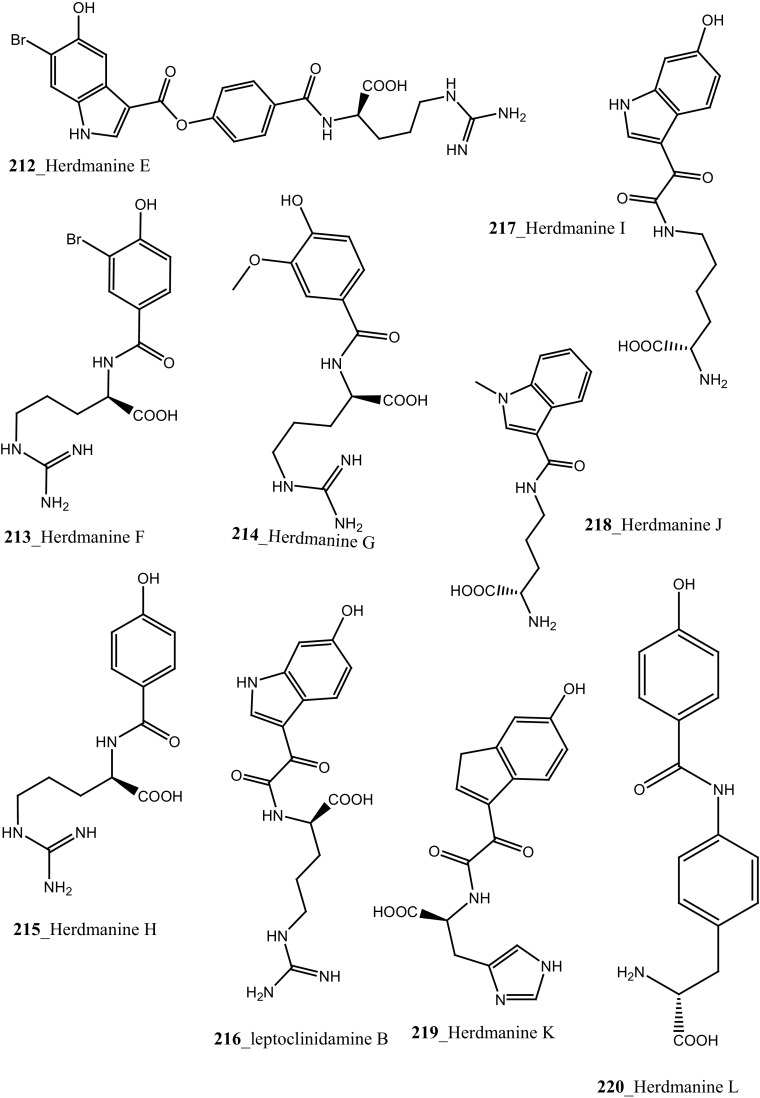
Anti-inflammatory potential compounds (**161**, **163**, **177**, **206**–**220**)

### Effect of Cardio Vascular Blockage and Central Nervous System

Marine alkaloid, lepadiformines A, B, C (**221**–**223**) have been reported from *Clavelina moluccensis* collected in Musha Islands, Djibouti [125]. Compounds (**221**–**223**) showed a potential action in frog atrial myocytes by blocking the background inward rectifying K^+^ current, the biological activity of these each compounds varied basis on their chemical structure. The length of aliphatic chain at position C13 was involved in K_**r**_ channel blockage of *I*K_1_ by lepadiformine A (1.42 µM), lepadiformine B (1.56 µM) in cardiac muscle and lepadiformine C was poorly blocked *I*K_1_.

Structurally similar too related quinolizidine and decahydroquinoline alkaloids, (−)-pictamine and (−)-lepadin B (**224**–**225**) were reported from *Clavelina picta* and *C. lepadiformis*. The pharmacological properties of compounds (**224**–**225**) were tested at neuronal nicotinic acetylcholine receptors (nAChRs) including (α4β2 and α7) expressed in *Xenopus* oocytes. Both compounds (**224**–**225**) were found to act as reversible blockers; acetylcholine-elicited currents through α4β2 and α7 receptors were blocked by (−)-pictamine (IC_50_ 1.5 and 1.3 mM), and by (−)-lepadin B (IC_50_ 0.9 and 0.7 mM), respectively [126, 127]. Compound, eusynstyelamides A–C (**226**–**228**) and known metabolites homarine and trigonelline were reported from *Eusynstyela latericius* collected in Great Barrier Reef, Australia [128]. The spectral data of eusynstyelamide A (**226**) was similar to previously reported compound eusynstyelamide from a Fijian Ascidian *E. misakiensis* [129], remarkably, the compound showed opposed specific rotations. This study proved that the structure of eusynstyelamide was elucidated mistaken by Swersey et al. [129] and the authors have predicted the correct chemical structure of this compound and named as eusynstyelamide A. Both compounds **227, 228** showed neuronal nitric oxide synthase inhibition IC_50_ values of 4.3–5.8 µM.

Bioassay-guided isolation resulted in identification of new tyrosine derivatives, botryllamides K, L (**229, 230**), with 6 known compounds, botryllamides A–C (**231**–**233**), botryllamide G (**234**) and perspicamides A and B (**235**–**236**) from the ascidian *Aplidium altarium* collected in Australia [130, 131]. These tyrosine derivatives were tested for their cytotoxicity against the SF268 (central nervous system), from these botryllamides K and C showed potent cytotoxicity inhibition (IC_50_ 78, 75 µM) at concentration 10 µM/mL. Recently, botryllamide G was reported as a potent inhibitor of the membrane-localized human transporter protein ABCG2 [132] (Structure 9).

**Structure 9:**
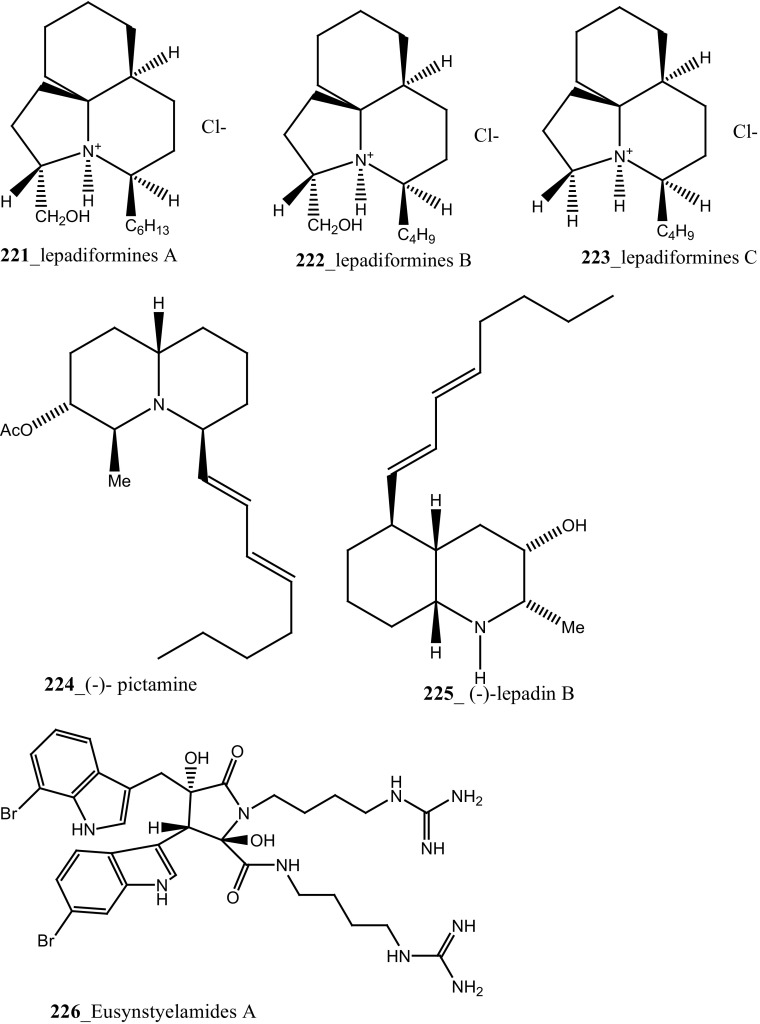

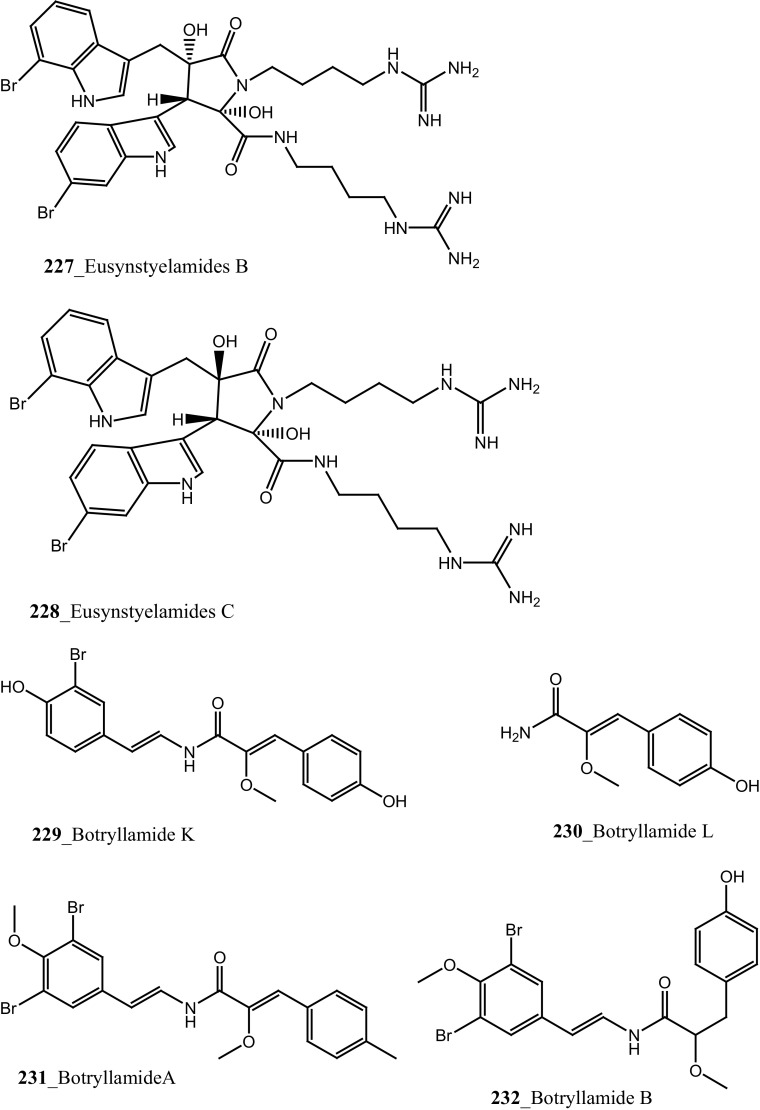

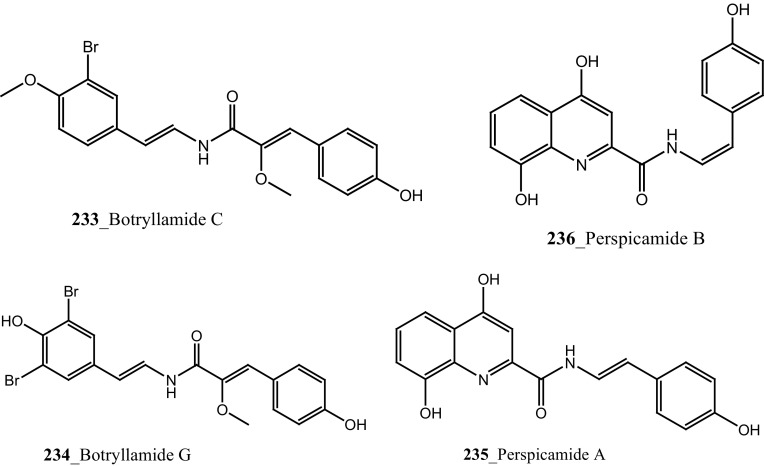
Compounds with potential activity of cardio vascular blockage and central nervous system (**223**–**236**)

Chem-biological evaluation of methanol crude extract from the ascidian *Eudistoma virde* showed a dose-dependent depression of locomotor activity, indicating central nervous system (CNS) depressant activity. The extract at 200 mg/kg concentration showed substantial depression activity of 90.7% which was lower compare to the positive control drug chloropromazine 99% at 4 mg/kg concentration [124]. The similar results of CNS depressant activity in the crude compounds of *Distapila nathensis* at concentration 100 mg/kg was reported earlier [133].

### Anti tumor/Anti-cancer, Anti-proliferative Activity

The drug discovery of anti-cancer activity has been the main area of interest in the field of natural product chemistry. Tunicates are particularly successful to yielding anti-tumor compounds; several MNPs were under various clinical trials in Europe and USA. With the continuous searching of effective and targeted anti-cancer drugs, in this section we explore the unique perspectives, novel biomolecules isolated from marine ascidians with promising pharmacological potential source of anti tumor/anti-cancer and anti-proliferative activity.

Three new linear cytotoxic tripeptides, virenamides A–C (**237**–**239**) have reported from the Didemnid ascidian *Diplosoma virens* collected in Great Barrier Reef, Australia [134]. Virenamide A (**237**) exhibited effective cytotoxicity against various cultured cells with P388 (IC_50_ 2.5 µg/mL), and showed moderate cytotoxicity against A549, HT29, and CV1 cells line (IC_50_ 10 µg/mL). Compounds (**238**–**239**) showed modest cytotoxicity against P388, A549, HT29, and CV1 cells. Additionally, virenamide A (**237**) showed topoisomerase II inhibitory activity (IC_50_ 2.5 µg/mL). Lindsay and co-workers have been isolated pyrido [2, 3, 4-*kl*] acridine-based alkaloid ascididemin 2 (**55**) from the *Didemnum* sp. [135]. Ascididemin 2 showed remarkable significant cytotoxicity against human tumor cells; P-388 mouse leukemia (IC_50_ 0.4 µM), human colon HCT II6 (IC_50_ 0.3 µM), and with human breast MCF7 (IC_50_ 0.3 µM), respectively.

In continuing search of MNPs to find anti-tumor drugs, Koulman et al. [136] was tested the cytotoxicity of colonial ascidian *Didemnum lahillei*, *Aplidium glabrum, Molgula manhattensis* collected in Lake Grevelingen and Oosterschelde estuary, Netherland. The crude extract of *A. glabrum* and *M. manhattensis* showed modest cytotoxicity against colon adenocarcinoma cells (IC_50_ 5, 8 µg/mL). The crude compound of *D. lahillei* showed moderate cytotoxicity against colon adenocarcinoma cell lines (IC_50_ 33 µg/mL) and with small cell lung carcinoma (IC_50_ 49 µg/mL), respectively. Two new dimeric alkaloids, lissoclinotoxins E, F (**68, 69**) were isolated from the Philippine didemnid ascidian collected in Sabtang Reef, Batanes Islands [137]. Both compounds (**68, 69**) showed potent cytotoxicity growth inhibition against MDA-MB-468 human breast carcinoma cell line (IC_50_ 2.3, 1.5 µg/mL), respectively. Lissoclinotoxins E and F shown potent cytotoxicity towards the MDA-MB-468 (PTEN^−/−^) and the MDA-MB-435S (PTEN^+/+^) cell lines. Lissoclinotoxin F showed a threefold higher cytotoxicity against the PTEN deficient cell line.

During the chemical investigation of Brazilian tunicates *Didemnum granulatum,* new alkaloid didemnimide E (**242**), and a new G2 checkpoint inhibitor and known marine alkaloids didemnimides A (**240**) and D (**241)** were reported [138]. Further investigation, authors worked in the same ascidian species was reported isogranulatimide (**37**) with two minor compounds granulatimide (**243**) and 6-bromogranulatimide (**244**) [139]. Both compounds (**243, 37**), showed activity as an inhibitor of the G2 cell cycle checkpoint in vitro and combined with DNA damaging p53 cancer cells (IC_50_ 1–1.8 µM). Anti-cancer effective marine alkaloid, mollamides B, C (**154, 155**) reported from *Didemnum molle.* Mollamide B (**154**) showed modest cytotoxicity against non-small cell lung cancer H460 (IC_50_ 29 µM), CNS cancer cell line SF-268 (IC_50_ 42 µM), and breast cancer cell line MCF7 (IC_50_ 44 µM) at concentration 100 µM [89]. Mollamide C exhibited poor cytotoxicity with murine leukaemia L1210 cells and human breast MCF-7 cells lines.

From the Australian ascidian *A. confluata*, marine alkaloid, aplidiopsamine A (**178**) was reported, compound (**178**) exhibited moderate cytotoxicity against HEK-293 cell line (IC _50_120 µM) at higher doses [102]. From the crude extracts of tunicate *Leptoclinides dubius,* the first *Leptoclinides*-derived indole compounds: *N*-(1*H*-indolyl-3-carbonyl)-d-arginine (**7**), *N*-(6-bromo-1*H*-indolyl-3-carbonyl)-l-arginine (**8**), *N*-(6-bromo-1*H*-indolyl-3-carbonyl)-l-histidine (**9**) and *N*-(6-bromo-1*H*-indolyl-3-carbonyl)-l-enduracididine (**10**) were reported by García et al. [17]. Remarkably, the major group of these compounds are nitrogen-containing compounds with aromatic heterocycles. Compounds (**7**–**9)** showed 80–100% inhibition of cytotoxic against P-388 cells and KB cells at 10 µg/mL.

Furthermore, four new meroterpenoids such as conidione (**245**), conicol (**246**), 2-[(1′*E*)-3′-methoxy-3′,7′-dimethylocta-1′,6′-dienyl]benzene-1,4-diol (**247**) and conitriol (**248)**, and five known compounds and their derivatives (**76**, **249**–**253**) were isolated from the Mediterranean ascidian *Aplidium conicum* collected in Tarifa Island, Spain [140]. In further investigation of the same ascidian species which was collected from Alghero, Italy; two new unique alkane derivatives, conicaquinones A **(254)** and B (**255**) were reported by Aiello et al. [140]. Compounds (**254, 255**) exhibited potential cytotoxicity against rat glioma cells (IC_50_ 2.1, 5 µg/mL). In continuing of search MNP from the same ascidian species, Aiello and co-authors isolated the unusual sulfone heterocycles aplidinones A–C (**256**–**258**) and the thiaplidiaquinones A and B (**259**, **260**) [141, 142]. Among these prenylated benzoquinones **(259, 260) showed** potent cytotoxicity against human leukemia T Jurkat cells (IC_50_ 3 µM). Furthermore, Aiello and co-authors, have recently synthesized and confirmed the structural assignments of compound aplidinone A (**256**) and reported the cytotoxicity and pro-apoptotic of a series of synthetic analogues of aplidinone A against kidney cancer 293T cells (IC_50_ 8.7 µM) and human lung adenocarcinoma A549 (IC_50_ 13 µM). Aplidinone A synthetic analogue potentially inhibits the TNFα-induced NF-KB activation in human leukemia T cell line [143].

During further investigation from the same ascidian *A. conicum* collected in Porto Cesareo, Italy: Menna and co-workers reported two new meroterpenes, the conithiaquinones A (**261**) and B (**262**), and known metabolite chromenols A, B (**263, 264**) and conicaquinone (**254**) [144]. Both compounds (**261, 262)** exhibited significant effects on the growth and viability of cells, compound (**261)** shows moderate cytotoxicity inhibition against human breast cancer cells (IC_50_ 44.5 µM). It is noticed from all the above investigations, Mediterranean ascidian species *A. conicum* have been extensively studied well. A rare collection of this tunicates depends on is that habitat and the abundance of its meroterpenes apparently depend on the geographical place of collection [144].

Two new tryptamine-derived alkaloids, leptoclinidamide (**11**) and (−) leptoclinidamine B (**12**), along with *C* ^2^-*α*-D-Mannosylpyranosyl-l-tryptophan (**14**) were isolated from the tunicate *Leptoclinides dubius* [19, 145]. The complete stereochemistry of compound (**14)** was resolved by circular dichroism. Compounds **11** and **14** showed modest cytotoxicity against two cancer cell lines HCT-15 (colon) and Jurkat (T cell lymphoma) cells at concentration < 30 µM. Addition, aromatic marine alkaloids, ningalins A–G (**265**–**271**) and known pyrrole alkaloids lamellarins G, Z (**147, 148**) were isolated from the tunicate *Didemnum* sp. collected in Northern Rottnest Shelf, Australia [146, 147]. Among these, ningalin G (**271**) showed potent inhibition against CK1δ, CDK5 and GSK3β kinases, and lamellarins showed significant inhibition against CDK5 only.

Staurosporines group of MNPs and their analogues are frequently reported in *Eudistoma* sp. [148]. Staurosporines, 11-hydroxystaurosporine (**272**), and 11-dihydroxystaurosporine (**273**) were reported from *Eudistoma* sp. collected at Micronesian island [149], additionally; the staurosporine aglicone (K252-c) **274** was isolated from same ascidian *Eudistoma* sp. collected at west coast of Africa [150]. In continuing search of anti-cancer drugs, Schupp and co-authors have reported three new indolocarbazole alkaloids, 3-hydroxy-4′-*N*-methylstaurosporine (**275**), 3-hydroxy-4′-*N*-demethylstaurosporine (**276**), 3′-demethoxy-3′-hydroxy- 4′-*N* demethylestaurosporine (**277)** and known metabolites staurosporine (**278**), 4′-N-methylstaurosporine (**279**), 3-OH-staurosporine (**280**), 3-OH-4′-*N*-methylstauro (**281**) and their derivatives from the ascidian *Eudistoma toealensis* [151–153]. Four metabolites were tested as inhibitors of cell proliferation with twelve human leukaemia cell lines. Among these, compound (**280**) showed potential anti-proliferative activities against MONO-MAC-6 cell line (IC_50_ 13 ng/mL). Though, from all these staurosporine derivatives, excluding 3′-demethoxy-3′-hydroxy- 4′-*N* demethylestaurosporine and 4′-*N*-methylstaurosporine was potentially reduced RNA and DNA synthesis. Compare to all the derived staurosporine, 3-hydroxystaurosporine is the most potential and highly effective staurosporine-type inhibitors discovered so far in the drug discovery process. This study demonstrated structure–activity relationships that hydroxylation of staurosporine at position 3 of the indolocarbazole moiety provoked to increase the anti-proliferative activity, while hydroxylation at carbon (C) 11 lead to decrease the biological activity of these derivatives. The authors also proposed that presence and absence of hydrophilic substitutions and the alteration of position in the biomolecules are vital in the anti-proliferative properties of staurosporine derivatives. It is worth to mention here, protein kinase is one of the major targets of staurosporine and have been widely used as molecular tools, the pharmacological potential variation of the staurosporine derivatives in regulating cell growth inhibition may due to differences in their ability to inhibit certain kinases involved in cell growth and tumour promotion [154, 155]. 7-hydroxystaurosporine (UCN-01) is currently in clinical phase trials II at the NCI-USA, UCN-01 strongly inhibits the growth of T cell lymphomas [147].

In continuing search of staurosporine MNPs from the tunicate, Jimenez and co-authors isolated two new staurosporine derivatives, 2-hydroxy-7-oxostaurosporine (**282**) and 3-hydroxy-7-oxostaurosporine (**283**) from the endemic ascidian *Eudistoma vannamei* collected in Taíba Beach, Brazil [156]. The mixture of metabolites (**282, 283)** showed strong cytotoxicity in vitro inhibition against Jurkat leukemia cells (IC_50_ 10.3 nM) and MOLT-4 leukemia (IC_50_ 18.64 nM). The presence of amino acid in staurosporine derivatives and their intermediate polarity, modulating the inhibition of growth in tumour cell lines. Remarkably, the amino acid-derived compounds showed apoptosis induction against HL-60 leukemia cells [157, 158].

Three sulfated alkene and alkanes, normonoterpenoid -(*R*)-2,6-dimethylheptyl sulfate (**284**), 6-methylheptyl sulfate (**285**), and (*E*)-5-octenyl sulfate (**286**) were isolated from the Mediterranean ascidian *Halocynthia papillosa* collected in Corigliano Gulf, Ionian Sea—Italy [159]. Metabolite, normonoterpenoid **(284)** was previously reported in ascidian *Policitor adriaticus* and in the hepatopancreas of *Halocynthia roretzi* [44, 160]. The metabolites **284**–**286** exhibited moderate anti-cancer activity against the WEHI-murine fibrosarcoma cells (20.9, 15 and 12.2 µg/mL) and shown weak activity against rat glioma C6 cell line.

New congeners, botryllamide G (**234**) and botryllamides E, F (**287**–**288)** and revision of the structure of botryllamide H (**289)** were reported from the ascidian *Botryllus tyreus* collected in Papua New Guinea [131, 161]. The metabolites (**234**, **287**–**288**) showed moderate anti-cancer activity against the breast cancer carcinoma (IC_50_ values of 6.9, 27, 41 µM), respectively.

Three new polysulfur alkaloids, lissoclibadins 1–3 (**65, 66, 67),** lissoclibadins E, F **(68, 69)** and two monomeric compounds **(290**–**291**) were reported from the ascidian *L. badium* [51, 52, 162] Lissoclibadin 1 (**65**) has a unique trimeric structure [51, 52] and compound **66** and **67** are dimers of the same unit as compound **65**. Further, six dimeric polisulfides [52, 83, 137, 162] and more than 10 monomeric cyclic polysulfides [48, 163] were isolated from the above ascidians. The polysulfur alkaloids, compounds (**65**–**67)** showed remarkable cytotoxicity against HL-60 cells (IC_50_ values of 0.37, 0.21, 5.5 µM). In addition, compounds (**66, 68)** exhibited promising cytotoxicity against VL 79 cells (IC_50_ values of 0.8, 0.28 µM). Compound (**66**) exhibited significant potential growth inhibition against colon cancer cell lines DLD-1 and HCT116 (2.88, 2.46 µM), and also showed strong activity against breast (MDA-MB-231) 0.2 µM, renal (ACHN) 0.24 µM, and large-cell lung (NCI-H460) cancer cell lines 0.64 µM, respectively. The authors suggesting that the anti-cancer efficacy of compound (**66**) is need to examined using in vivo a nude mouse model bearing a human solid tumor [163].

Marine alkaloids, haouamines A and B **(292, 293)** were isolated from the ascidian *Aplidium glabrum* collected from Tariffa Island, Spain [164]. The metabolite (**292**) shows remarkable cytotoxicity in vitro inhibition against HT-29 Cell line (IC_50_ 0.1 µg/mL), and haouamine B (**293**) showed modest cytotoxicity against the MS-1 cell line (IC_50_ 5 µg/mL). Fedorov and co-authors was isolated, anti-cancer effective metabolite 3-demethylubiquinone Q2 (**294**) and prenylquinones (Quinone 7, 8) **295** from the same ascidian *Aplidium glabrum* [165]. Compounds (**294, 295a**–**d**) showed moderate cytotoxicity against JB6-Cl41 mouse epidermal cells (IC_50_ 11.4, 8.3, 5.1, 4.7, 4.6 µM); which may be attributed to the induction of p53-independent apoptosis.

During the chemical investigation, Tadesse and co-authors was isolated marine alkaloid synoxazolidinone A (**86**) from the ascidian *Synoicum pulmonaria* [58]. Metabolite (**86**) showed modest cytotoxicity against HT-29 colon carcinoma cells (IC_50_ 30.5 µM) and also killed normal lung fibroblast cells (MRC-5) at the same concentration. This suggests that the additional pyrrolidine ring of compound (**85**) modulating cell growth and increasing cell cytotoxicity. The known rubrolide derivatives, prunolide A (**138**) was isolated from the Indian ascidian *Synoicum* sp. [83]. Compound **(138)** exhibited potent cytotoxicity against breast cancer cell lines at a concentration of <1 µM.

Two new dibrominated marine acetylcholinesterase inhibitors pulmonarins A, B (**296, 297**) were isolated from the sub-Arctic ascidian *Synoicum pulmonaria* collected off the Norwegian coast [166]. Compound (**297**) showed potent inhibition constant (*K*_*i*_) of 20 µM. Three new indole alkaloid derivatives, ethyl indolyl-3-glyoxyiate (**298**), ethyl 6-bromoindolyl-3 glyoxylate (**299**), ethyl 6-bromo-5-hydroxyindolyl-3-glyoxylate (**300**), in addition with known compound, hydroxyphenylglyoxylate, 2,6-dimethylheptyl sulfate and (3*Z*)-3-decenyl sulphate were isolated from the ascidian *Syncarpa oviformis* collected in Kuril Islands, Russia [167]. The metabolite (**298-300)** showed moderate cytotoxicity against Ehrlich carcinoma cells (EC_50_ values of 61, 35, 97 µg/mL), respectively. Additionally, compound **300** showed moderate inhibition of non-specific esterase activity in mouse lymphocytes up to 44.2% compared with control cells at concentration 100 µg/mL.

Davis and co-workers isolated a new *β*–carboline alkaloid, eudistomin V (**301**) with known compound eudistomins H and I (**302, 303**) from the Australian ascidian *Pseudodistoma aureum* collected at Heron reef [168]. Furthermore, three *β*–carboline alkaloids, 2-methyleudistomin D (**304**), 2-methyleudistomin J (**305**), and 14-methyleudistomidin C (**306**) along with known metabolites, eudistomins C (**307**), D (**308**) E (**309),** J–L (**310**–**312**) were also isolated from the ascidian *Eudistoma gilboverde* [36]. Compound (**306**) showed the most potent cytotoxic activity with average (IC_50_ < 1.0 µg/mL) against four different human tumor cell lines, LOX (melanoma), 0.41 µg/mL; COLO-205 (colon), 0.42 µg/mL; MOLT-4 (leukemia), 0.57 µg/mL; OVCAR-3 (ovarian), 0.98 µg/mL, respectively.

Novel polycyclic alkaloids, perophoramidine (**313**) was isolated from Philippine ascidian *Perophora namei* [169]. Perophoramidine consist of an unusual carbon skeleton structure and showed modest cytotoxic against colon carcinoma cell line HCT-116 (IC_50_ 60 µM), which includes and induces apoptosis via PARP cleavage within 24 h. Two novel alkaloids, sebastianine A (**314**) and sebastianine B (**315**) reported from the *Cystodytes dellechiajei* collected in Sao Sebastiao Island, Brazil [170]. Sebastianine A and B showed remarkable cytotoxicity against HCT-116 cell lines comprised of p53 (IC_50_ 5.1, 0.92 µg/mL) and p21 (IC_50_ 1.3, 1 µg/mL) knockouts as well as the parental cell line of each. Martinez-Garcia and co-authors [171], studied anti-tumor activity of the same asicidian *C. dellechiajei* crude extracts against various tumor cell lines. Crude extracts displayed most potent cytotoxicity against human lung carcinoma A-549 (IC_50_ < 2.5 µg/mL), HCT-116 (IC_50_ 2.5 µg/mL), pancreatic adenocarcinoma PSN-1 (IC_50_ 5 µg/mL) and breast carcinoma SKBR3 cell lines (IC_50_ 5 µg/mL), respectively.

Four new pyridoacridine alkaloids, shermilamine F (**316**), dehydrokuanoniamine F (**317**), and arnoamines C (**318**) and D (**319**), and known shermilamine C (**320**) and dehydrokuanoniamine B (**321**) were isolated from the *Cystodytes violatinctus* collected in Solomon Islands [23, 172]. A general hypothetical biogenetic pathway suggested that pyridoacridine alkaloids contain a fused pyrrole ring. Compound **319** showed promising anti-cancer activity against HCT-116 (IC_50_ 4.32 µM), SW480 (colon) cancer cell line (IC_50_ 8.48 µM) and A375 (melanoma) cells (IC_50_ 6 µM), respectively.

Four new lamellarin alkaloids, lamellarin-ζ (**322**), lamellarin-η (**323**), lamellarin-ϕ (**324**) and lamellarin-χ (**325**) and 7 known amino acid derived lamellarins, lamellarin-K (**199**), lamellarin-K triacetate (**201**), lamellarin-I (**203**), lamellarin-J, lamellarin-L triacetate, lamellarin-F and lamellarin-T diacetate (**326**–**329**) were isolated from the ascidian *D. obscurum* collected in Tiruchandur coast, Gulf of Mannar, India [173]. Compounds (**322, 325, 328, 329)** were showed potential cytotoxicity against colorectal cancer cells (COLO-205) [173]. From this, both compounds lamellarin-χ and lamellarin-I was exhibited sensitizing effects to doxorubicin in multidrug resistant P388/schabel cells (IC_50_ 0.0002 µM) and displayed very strong activity at a concentration 10 times lower than that of the prototype MDR inhibitor verapamil [174].

Fatturuso and co-workers was reported a new antiproliferative sulphate alkene, Cll alkyl sulfate, (3*Z*)-4,8-dimethylnon-3-en-l-sodium (**330)** from the Mediterranean ascidian *Microcosmus vulgaris.* Compound **330** showed most active anti-proliferative activity against bovine endothelial GM7373 (IC_50_, 48 µg/mL) and WEHI 164 murine fibrosarcoma cells (IC_50_ > 55 µg/mL) [175]. Alkyl sulphate, 3,7,11,15-tetramethyl-hexadecan-l,19-sodium disulfate (**331**), heneicosane-l,21-sodium disulfate (**332**) were reported from the Mediterranean tunicate *Ascidia mentula* showed modest anti-proliferative activity against human melanoma IGR-1 (IC_50_ values of > 110, 100 µg/mL) and with murine monocyte/macrophage J774 (IC_50_ > 180, > 170 µg/mL), respectively [176].

The secondary metabolite indoles, 3,6-dibromoindole (**333**), 6-bromo-3-chloroindole (**334**) and 6-bromo-2-oxindole (**335**) were isolated from the tunicate *Distaplia skoogi* collected in Algoa Bay, South Africa [177]. To date the only one MNPs reported from the genus *Distaplia* is 3,6-dibromoindole (**333**) which was isolated from *D. regina* collected in Malakal Harbour, Palau [178]. Compounds (**333**–**335**) showed moderate cytotoxicity against MDA-MB-231 breast cancer cells (IC_50_ 117.72, 72.53, 74.41 µM), respectively. Recently, a series of glycosylated polyketide macrolides, mandelalides A–D (**336**–**340**), were isolated from the ascidian *Lissoclinum* sp. collected in Algoa Bay, South Africa [179]. Compounds (**336**–**337**) exhibited most potent cytotoxicity against human NCI-H460 lung cancer cells (IC_50_ 12, 44 nM) and mouse Neuro-2A neuroblastoma cells (IC_50_ 29 and 84 nM), respectively.

Rudolph and co-researchers reported, four new acylated pteridine alkaloids, duramidines A–D (**341**–**344**), two new acylated thymidine alkaloids, leptoclinidines A and B (**345, 346**), two new 1-acylglyceryl-3-(*O*-carboxyhydroxymethylcholine) alkaloids, durabetaines A and B (**347, 348**), three new 1,3-dimethyl-5-methylsulfanylimidazole alkaloids, leptoclinidamines D–F (**349-351**), and the known alkaloids leptoclinidamines B and C (**12, 13**) and 6-bromo-1*H*-indolo-3-yl-oxoacetic acid methyl ester (**352**) from the Australian ascidian *Leptoclinides durus* [18, 180]. The duramidines are the first pteridine alkaloids, possessing a 3 carbon side chain esterified at C-1′ with a 4-hydroxy-2′-methoxycinnamic acid, and are either hydroxylated or sulfated at C-2′. The leptoclinidines are the first 3′-indole-3-carboxylic acid ester derivatives of thymidine so far reported in the literature. The durabetaines are the first glyceryl-3-(*O*-carboxy hydroxyl methylcholine) alkaloids have been reported from an animal source and are also the only known derivatives from this class to be acylated with aromatic carboxylic acids. Durabetaines A and B (**347, 348**) are related to lipid esters that have been previously reported from algae [181, 182]. The significant difference between these algal products and the durabetaines is the replacement of the long chain fatty acid attached at C-1 by aromatic esters. Furthermore, the algal derivatives reported to date have all contained a second-long chain fatty acid attached at C-2. All the compounds were indeed completely inactive against prostate (LNCaP) and breast (MDA-MB-231) cancer cell lines and anti-microbial activity against *Pseudomonas aeruginosa* and *Staphylococcus aureus* [180].

Further, six new 2-amino-3-alkanol products of clavaminols A–F (**353**–**358**) and their derivatives (**358a**) were isolated from the Mediterranean ascidian *Clavelina phlegraea* [183]. In addition to continuing search of new series amino alcohols by Aiello and co-researchers, have isolated another six marine sphingoids, clavaminols G–N from the same ascidian species [184]. Clavaminol A (**353**) was showed moderate cytotoxicity against AGC cells (IC_50_ 5 µg/mL) and compound (**354**) showed less cytotoxicity than compound (**353**), these signifying that additional unsaturation was caused cell growth inhibition and clavaminols C and F were completely inactive against AGG cells [183]. Four new labdane alkaloids, haterumaimides A–K (**359**–**369**), together with dichlorolissoclimide (**370)** and chlorolissoclimide (**371**) were isolated from the tunicate *Lissoclinum* sp. collected from the coast of Hateruma Island [185–187]. Further investigation from the same ascidian, four new labdane alkaloids, haterumaimides N–Q (**372**–**375**) were reported by Uddin et al. [47]. Compounds (**373, 374**) showed potent cytotoxicity against P388 cells (IC_50_ 0.23, 0.45 ng/mL), respectively. Compounds **370, 371** exhibited moderate cytotoxicity against P388 cells with IC_50_ 4.1 and 5.5 ng/mL and the other compounds showed poor cytotoxicity compared to control one.

Four new *β*-carboline metabolites, didemnolines A–D (**54**–**57**), were isolated along with known compound eudistomin O (**376**), *β*-carboline (**377**) and 2-(2′,4′-dibromophenoxy)-3,5-dibromophenol (**378)** from the tunicate *Didemnum* sp. collected in Northern Maxima Islands [188]. These new *β*-carboline-based metabolites are varied from the earlier reported compounds in that they are substituted at the N9 position of the *β*-carboline ring rather than the Cl position. While, eudistomidins D, E, F is the only marine alkaloids bearing substitution at N9. Didemnolines A–C showed potent cytotoxicity against epidermoid carcinoma (KB) cells (IC_50_ 6.1, 3, 0.28 µg/mL), respectively.

Fu and co-workers reported the dimeric prenylated quinone longithorone A (**379**) was isolated from the ascidian *Aplidium longithorax.* Further, around sixteen related monomeric and dimeric compounds include the longithorone B-I (**380**–**387**) [189, 190]. Longithorones J and K (**388**–**389**) [191], longithorols A, B (**390**–**391)** [192], Longithorols C–E (**392**–**394**) [193], floresolides A–C (**395**–**397**) [53] were isolated from the same ascidian species. Floresolides possessing a *ε*-lactone bridging at aromatic ring and metacyclophane band are unique members of the class of secondary metabolites known as longithorones and longithorols from the ascidian *A. longithorax*. Longithrone A showed poor cytotoxicity against P-388 murine leukemia cells ED_50_ > 10 µg/mL [189]. Compounds (**395**–**397**) exhibited modest cytotoxicity against epidermoid carcinoma (KB) cells (IC_50_ 1–10 µg/mL) [53].

New pyridoacridine alkaloids, lissoclinidine (**58**), diplamine (**61**) were isolated from the New Zealand ascidian *L. notti* [48]. Compounds (**61, 58**) showed potent cytotoxicity against P388 D1 murine leukaemia cell line (IC_50_ values of 1.9, 4.6 µM), followed by HCT-116 human colon tumour (<1.4, 3 µM), respectively. Both compounds showed cytotoxicity inhibition against Non-malignant African Green Monkey kidney BSC-1 cells (4 + mm) at concentration of 60, 10 µg. New polysulfur alkaloids, Lissoclibadins (**68**–**71**) were isolated from the ascidian *L. badium* [8] compound (**68**–**71**) showed moderate inhibition against colony formation of Chinese hamster V79 cells (EC_50_ values of 0.71, 0.06, 0.06, and 0.17 µM), respectively. In continuing searching of potential MNPs from the same ascidian, Wang and co-workers [194] isolated the polysulfur dopamine-derived alkaloids, lissoclibadins 8–14 (**398-404**). Lissoclibadins 8, 14 (**398, 403**) exhibited potent cytotoxicity against Chinese hamster V79 cells (IC_50_ 0.20, 0.17 µM) and with murine leukemia L1210 cells (IC_50_ 1.33, 1.12 µM), respectively. Also, lissoclibadins 12–13 (**402**–**403**) have showed potential cytotoxicity against murine leukemia L1210 cells (IC_50_ 0.32, 0.53 µM). Compound, 2,6-dimethylheptyl sulfate (**405**) was isolated from the ascidian *Policitor adriaticus* collected in North Adriatic Sea [10]. Compound **405** have showed moderate cytotoxicity (IC_50_ 17.8 µg/mL) with *Artemia salina* bioassay and it indeed completely inactive at concentration 100 µg/mL with the lethality assay against *Gambusia affinis* fish.

A new series of glucosphingolipids, phallusides 1–4 (**406**–**409)** were reported from the Mediterranean ascidian *Phallusia fumigata* collected in Tariffa, Spain [195]. This is the first study reporting that glucosphingolipids from the tunicates. Phallusides 1–3 (**406**–**408)** hold uncommon sphingoid base 2-amino-9-methyi-D-erythro-(*4E,8E,10E*)-octadeea-4.8,10-triene-l,3-diol. The secondary metabolite, (*R*)-2,6-dimethylheptyl sulphate was previously reported from the ascidian *P. adriaticus* [10]. The compounds **406, 407** showed cytotoxicity against human colon carcinoma (HT 29) cells (ED_50_ 10 µg/mL).

The disulfide alkaloids, polycarpamines A–E (**410**–**414**), from the ascidians *Polycarpa aurata* [196]. In continuing search of potent MNP from the ascidian *Polycarpa clavata* collected in Australia [197] isolated dimeric disulfide alkaloid, Polycarpine dihydrochloride (**415**) and with four related compounds (**416**–**419**). Compound **415** showed potent cytotoxic against the human colon tumor cell line HCT-116 (IC_50_ 0.9 µg/mL). Further investigation in from the ascidian *Polycarpa aurata* collected Micronesial, two new alkaloids, polycarpine and *N*,*N*-didesmethylgrossularine-1 (**420**–**421**) were isolated by Abas et al. [198]. Wessels et al. [199] reported three new compounds, *N*-(4-methoxybenzoyl)-*N*′-methylguanidine **(422**), butyl 2-(4-methoxyphenyl)-2-oxoacetate (**423**), and 2-(4-methoxyphenyl)-*N*-methyl-2-oxoacetamide (**424**), and known metabolites methyl 2-(4-methoxyphenyl)-2-oxoacetate (**425**) and 4-methoxybenzoic acid (**426**) were isolated from the same ascidian collected from Great Barrier Reef, Australia. Wang and co-workers [200] reported three new sulfur-containing alkaloids, polycarpaurines A, B, C (**427**–**429**) along with six known compounds (**430**–**435)** were isolated from the ascidian *P. aurata* collected at in North Sulawesi, Indonesia. Compound (**420**) showed most potent inhibition against IMPDH inhibitor (IC_50_ 0.03 µM). Polycarpaurines A, C (**427, 429**) showed potent cytotoxicity against Chinese hamster V79 cells (EC_50_ 6.8, 3.8 µM), respectively. Compounds **428, 434** showed modest inhibition against V79 cells (EC _50_ > 10 µM), and induction of apoptosis in JB6 cells through p53- and caspase 3-dependent pathways. Two new Thiadiazole alkaloids, polycarpathiamines A and B (**436, 437**), were isolated from the ascidian *Polycarpa aurata* [201]. Compound **436** showed potential cytotoxic activity against L5178Y murine lymphoma cells (IC_50_ 0.41 µM).

Anti-leukemic effective compounds, three methyl esters (methyl myristate, methyl palmitate and methyl stearate) **438**–**440**, four steroids (cholesterol, campesterol, stigmasteroland *β*-sitosterol) **441**–**444**, two fatty acids (palmitic acid and stearic acid) **445**–**446**, three glyceryl ethers {(1,2-propanediol, 3-(heptadecyloxy), batyl alcohol and 1,2-propanediol, 3-[(methyloctadecyl)oxy]} **447**–**449** and two nucleosides (thymidine and 2′-deoxyguanosine) **450**–**451** from the ascidian *Didemnum psammatodes* were collected in the intertidal zone of the Flexeiras beach rocks, Brazil [202]. The mixture of three methyl esters showed modest cytotoxicity against promyeloblastic leukemia HL-60 (IC_50_ values of 9.49, 6.91, 14.33 µg/mL), chronic myelogenic leukemia K-562 (IC_50_ values of 8.95, 5.62, 14.26 µg/mL) lymphoblastic leukemia CEM (IC_50_ values of 9.96, 4.33, 22.90 µg/mL) and T-cell leukemia Molt-4 (IC_50_ values of 2.43, 1.64, 3.62 µg/mL), respectively. Furthermore, Plakinidine D (**452**), 3,5-diiodo-4-methoxyphenethylamine (**453**), and ascididemin (**5**) reported from the ascidian *D. rubeum* collected in Indonesia [203]. Compound (**452**) showed promising anti-cancer activity against HCT-116 cell line (IC_50_ 5 µg/mL). Non-nitrogenous compounds, 3 new fatty acid metabolites, didemnilactones A and B and neodidemnilactone (**454**–**456**) were isolated from the tunicate *Didemnum moseleyi* [204]. All the metabolites showed cytotoxicity inhibition with lipoxygenase and weak binding activity to leukotriene B4 receptors. Marine alkaloids, fascaplysin (**457**) and 3-bromofascaplysin **(458)** were isolated from ascidian *Didemnum* sp. collected in Chuuk atoll, Indonesia [33]. The compound (**458)** showed poor cytotoxicity against both murine C 38 and human colon HCT-116 cell lines, and compound **457** was indeed completely inactive against with both tumour cells.

Two new anti-cancer compounds, arnoamines A (**459**) and B (**460**) were isolated from the *Cystodytes* sp. collected in Marshall Islands [34]. Compound **459** showed potent cytotoxicity against the MCF-7 breast cancer (IC_50_ 0.3 µg/mL), A-549 lung cancer (IC_50_ 2 µg/mL), and HT-29 colon cancer (IC_50_ 4 µg/mL), respectively. Also, compound (**460)** exhibited modest inhibition against the MCF-7, A-549 and HT-29 cell lines (IC_50_ 5, 2, 3 µg/mL) [205].

In addition, namenamicin (**128**) and 3 other new metabolites compounds from the enediyne class shishijimicins A–C (**461**–**463**) were isolated from the ascidian *D. proliferum* [75]. Namenamicin (**128**) was previously reported in Fijian tunicate *Polysyncraton lithostrotum* [206], compounds (**128, 461**–**463**) showed potent cytotoxicity against tumor cell lines; fibroblast line 3Y1 (IC_50_ 2, 3.1, 4.8, 13 pg/mL), Helacyton gartleri (HeLa) (IC_50_ 1.8, 3.3, 6.3, 34 pg/mL), and with P-388 mouse leukemia (IC_50_ 0.47, 2, 1.7, 3.3 pg/mL), respectively.

A series of new C_11_ cyclopenteno 1–7 (**464**–**470**), in addition with four known metabolites 9/10 (mixture of didemnenones A/B), 12–13 (**471**–**473**) were isolated from the didemnid ascidian *Lissoclinum* sp. In continuing search of MNP on another ascidian *Diplosoma* sp. were reported didemnenones 1, 2 and 5, (**464, 465, 468**) [207]. Compounds (**464, 467, 469)** showed potent cytotoxicity against tumor carcinoma, HCT116 (IC_50_ 3, 2.3, 1.8 ppm), human epidermal carcinoma A 431 (IC_50_ 6.4, 3.9, 3.1 ppm), and with human lung cancer (IC_50_ 4.8, > 20, 3.5 ppm), respectively.

Alkaloids compound, pibocin A, B (**474, 474a**), were isolated from *Eudistoma* sp. (Family Polycitoridae) collected in Japan [208]. Pibocin B is the first representative alkaloid of with a unique structural feature, an N–O methylindole group, the chemical structure was confirmed as (8*β*)-2-bromo-*N*–*O*-methyl-6,8-dimethylergoline by spectroscopic analysis and chemical correlations [208]. Compound **474** showed adequate cytotoxicity against mouse Ehrlich carcinoma cells (ED_50_ 25 µg/mL). Marine hydroperoxysterols, 7*β*-hydroperoxycholesterol (**475**) and its stereoisomer 7α-hydroperoxycholesterol (**476**), were isolated from the lipophilic extracts of ascidian *Eudistoma* sp. collected in southern Taiwan coast [209]. Compound **475** showed modest anti-cancer activity against human hepatoma cells Hep3B (IC_50_ 15.6 µg/mL) and human lung adenocarcinoma A549 (IC_50_ 17.4 µg/mL). Also, compound **476** showed modest cytotoxicity against human breast carcinoma MCF7 and MDA-MB-231 (IC_50_ 11.2, 11.2 µg/mL).

During continue searching of steroids from ascidians five new ecdysteroids, hyousterones A–D (**477**–**480**), abeohyousterone (**481**) and known ecdysteroid diaulusterol B (**482**) were the first reported ecdysteroids from the Antarctic ascidian *Synoicum adareanum* [210]. Compounds **478** and **480** are rare ecdysteroids in bearing the 14*β*-hydroxyl group, and abeohyousterone incorporates the 13 (14 → 8) abeo steroid skeletons, reflecting a rearrangement of the steroid C/D ring system [210]. Steroids (**477, 478, 481)** showed potent cytotoxicity against tumor cell HCT-116 cell line (IC_50_ values of 10.7, 3.7, 3 µM).

Choi and co-workers reported one isoprenoid, tuberatolide A (**483**), meroterpenoids tuberatolide B (**484)** and 2′-*epi*-tuberatolide B (**485**), known meroterpenoids yezoquinolide, (*R*)-sargachromenol, and (*S*)-sargachromenol (**486**–**488**) from the tunicate *Botryllus tuberatus* collected from near Tong-Yong, South Sea of Korea [211]. Compound **486** was previously reported from the marine brown algae, *Sargassum sagamianum serratifolium* and *S.* var. *yezoense* [212, 213]. Compounds **483**–**485** exhibited moderate cytotoxicity inhibition with hFXR transactivation (IC_50_ 3.9, 1.5, 2.5 µM). Compounds **484**–**485** exhibited poor cytotoxicity against CV-1 cell line (IC_50_ values of 31, 30 µM). Davies-Coleman et al. [214] reported 2 new 3,6-epidioxy-7,10-tetrahydrofurano C_26_ unsaturated fatty acids, stolonic acids A, B (**489**–**490**), from the ascidian *Stolonica* sp. collected at Maldive Islands. Compounds (**489**–**490**) showed most potent cytotoxicity against human melanoma and ovarian tumor cell lines (IC_50_ 0.05–0.1 µg/mL).

Anti-immunomodulatory activity of ethanolic extract of ascidian *Phallusia nigra* collected in Tuticorin harbour, India exhibited modest cytotoxicity against sarcoma 180 (S-180) cells at concentration 0.60 mg/mL [215]. Marine alkaloid, coproverdine (**491**) reported from the unidentified New Zealand ascidian species [216]. The similar related metabolites carbazomycins G and H was reported from microbes *Streptoverticillium ehimensein* [217]. Compound (**491**) showed remarkable cytotoxicity inhibition against several murine and human tumor cell lines: P388, A549 ((IC_50_ 1.6, 0.3) and with HT29, MEL28, DU145 (IC_50_ 0.3 µM), respectively.

New purine 1,3,7-trimethylisoguanine (**492**) was reported from the ascidian *Pseudodistoma cereum* collected in North New Zealand [218]. Previous investigations from the same ascidians led to the discovery of cytotoxic amines [219], aminols [220] and alkaloids [168]. Piperidine alkaloids, pseudodistomins A, B (**493**–**494**) were isolated from the Okinawan ascidian *Pseudodirtma kanoko* [220]. This study revised the structure of pseudodistomin A, was previously assigned with a 3′E, 5′Z-diene in the side-chain; it is now displayed to possess a 6′*E*,8′*Z*-diene. Further, Kobayashi et al. [221] was isolated and synthesized pseudodistomin C (**495**) from the same tunicate. In continue search of MNP from Pennsylvania ascidian *Pseudodistoma megalarva*, compound pseudodistomins D–F (**496**–**498**), pseudodistomins B and C (**494**–**495**) were reported [222]. Compound **492** showed poor cytotoxicity against tumor cell lines P-388 murine leukemia and SF-268 human CNS at concentration 100 µM. Compound, 1,3,7-Trimethylisoguanine was showed poor inhibition with cell cycle regulating enzyme cdc2/cyclin B kinase at concentration10 µM. Pseudodistomins B–F (**494**–**498**) have showed modest activity in a cell-based assay for DNA damage induction and noticed improved inhibition was due to an alternative mechanism. Pseudodistomins was completely inactive in glucose vs. galactose strain test, its indicating that pseudodistomins are killing yeast by any of alternative mechanism than by production of DNA damage [222].

Compound, 2-(3′-bromo-4′-hydroxyphenol) ethanamine (3′-bromotyramine (**499**) and known sponge metabolite 1,3-dimethylisoguanine (**500**) were isolated from *Cnemidocarpa bicornuta* collected in New Zealand [223, 224]. Compound (**499**) exhibited moderate cytotoxicity against the tumor cell line P-388 murine leukemia cell (IC_50_ 46 µM).

Yin and co-workers reported botryllamides K, L (**229, 230**) from the ascidian *Aplidium altarium* [131]. Both compounds (**229, 230**) showed poor cytotoxicity against MCF-7 breast cancer (IC_50_ 74, 91 µM) and H460-lung cancer cell line (IC_50_ 91, 89 µM) In continuing search of MNP from the same ascidian genus, four new brominated Quinolinecarboxylic Acids, caelestines A–D (**501**–**504**), reported from *Aplidium caelestis* collected in North Stradbroke Island, Australia [225]. All the compounds (**501**–**504)** showed poor cytotoxicity against MCF-7 breast adenocarcinoma cancer cells (IC_50_ 39, 49, 40, 38 µM), MM96L melanoma cell line (IC_50_ 62, 69, 54, 52 µM), and NFF (neonatal foreskin fibroblasts) (IC_50_ 57, 66, 58, 68 µM), respectively.

A new marine alkaloid, zorrimidazolone (**505**) and known alkaloids 3-indolylglyoxylic acid (**506**), methyl ester (**507**), 4-hydroxy-3-methoxyphenylglyoxylic acid methyl ester (**508**) were isolated from the Mediterranean ascidian *Polyandrocaroa zorritensis* [226]. Compounds **505**–**508** showed poor cytotoxicity against C6 rat glioma cell line (IC_50_ 155 µM), and all the compounds completely inactive against H9c2 (rat cardiac myoblast), and cervical cancer-HeLa cell line.

Furthermore, Menna and co-workers reported a series of alkyl sulfates (**509**–**513)** and 3 new metabolites **509**–**511** from the Mediterranean ascidians *Aplidium elegans* and *Ciona edwardsii* collected in Bay of Naples, Italy [227]. Compounds **509**–**510** showed moderate cytotoxicity against the BALB/c murine macrophages J774A.1 (IC_50_ > 100 µM) and C6 (rat glioma) cell lines (IC_50_ 45.12 µM), respectively. New tyrosine-iodinated derivative iodocionin (**514**) was reported from the same ascidian species *Ciona edwardsii* [228]. Compound (**514**) and brominated analogue (**515**) were previously reported in Caribbean sponge *Verongula gigantean* [229]. Compound **514** showed modest cytotoxicity against cancer cells, mouse lymphoma L5178Y (IC_50_ 7.75 µg/mL), and in active with PC12 (rat pheochromocytoma). Also, the comparison of efficacy of these derivatives (**514**–**515**) clearly stated the structure–activity relationship for the nature of the halogen atom present on the aromatic ring [229].

Fujita et al. [230] isolated sodium 1-(12-hydroxy) octadecanyl sulfate (**516**) from the unidentified ascidian species belongs to family Polyclinidae. Metabolite (**516**) is structurally related to the forbesins previously reported from the starfish *Asterias forbesi* [231]. Compound (**516**) showed modest inhibition against MMP2 (IC_50_ 90 µg/mL).

Six new bromoindole derivatives, aplicyanins A–F (**517**–**522**), were isolated from the ascidian *Aplidium cyaneum* collected in Antarctica [232]. The metabolites **518**–**522** showed significant cytotoxic and anti-mitotic activities. Compare to all the metabolites, compounds **518, 520** exhibited potent cytotoxicity against the tumour cell line A-549 (IC_50_ 0.66, 0.63 µM), HT-29 colorectal carcinoma (IC_50_ 0.39, 0.33 µM), and MDA-MB 231 (IC_50_ 0.42, 0.41 µM), respectively. Compounds **518, 520, 522** showed moderate anti-mitotic activity (IC_50_ 1.19, 1.09, 0.18–0.036 µM), whereas compounds **518, 519** was completely inactive at maximum concentration and compound **521** showed poor cytotoxicity. The above results demonstrating that key role for the presence of the acetyl group at N-16 in the efficacy of this group of compounds.

Kehraus and co-workers reported 5 new amino acid derivatives (**523–527**) from the *Atriolum robustum* collected in Great Barrier Reef [233]. Compound (**523**) contains a unique 3-(4-hydroxy-phenyl)-2-methoxyacrylic acid moiety, so far only reported in ascidian *Botryllus* sp. [130]. Compound (**526)** showed moderate inhibition with cAMP accumulation in Chinese hamster ovary (CHO) cell membranes and recombinantly exhibiting the human A_3_adenosine receptor, this clearly indicates that the adenosine derivative is partially acting against A3ARs. In radioligand binding analysis, 5′-deoxy-5′-methylthioadenosine-2′, 3′-diester 4 exhibited strong affinity with A_1_ and A_3_ adenosine receptors (K_i_ > 10 µM) and with A_2A_ and A_2B_ adenosine receptors (K_i_ 17 µM).

Four new sulphated derivatives, 1-heptadecanyl sulfate, 1-octadecanyl sulfate, sodium *(2S)*-2,6,10,14-tetramethylpentadeca-1,18-diyl sulfate, and 1-hexyl sulfate (**528**–**531**) were isolated from *Sidnyum turbinatum* collected in Italy [234]. Compound (**530**–**531**) showed modest anti-proliferative activity against murine fibrosarcoma WEHI 164 cells (IC_50_ 230, 150 µg/mL).

A series of meroterpenes derivatives reported from the ascidian *A. densum* by [54, 55] and demonstrated chemical synthesize [235]. Compounds, cordiachromene A (**76**), epiconicol (**77**) showed modest anti-proliferative activity against CCRF-CEM human leukemic lymphoblastics cells (IC_50_ 30 µM, 60 µM) [54]. Similar results of cytotoxicity of epiconicol reported [236] (IC_50_ 10 µg/mL) against the tumour cell lines (P388, A549, HT29, CV1). In addition, primary screening of methoxyconidiol had shown an adequate anti-proliferative activity against bacterial strains. The results of this study suggesting that the mechanism of the action of methoxyconidiol could be arbitrate by interruption of microtubule dynamics [55]. This led authors to synthesize methoxyconidiol together with epiconicol and didehydroconicol [235]. Synthesized meroterpenes, chromane (**532)**, chromene (**533**) and hydroquinone (**534)** have showed modest inhibition of egg division. Methoxyconidiol showed significant results on *P. lividus* eggs (IC_50_ 0.80 µM) and *S. granularis* (4.3 µM) on eggs and completely inactive with human serum-stimulated cells. Compounds **(77, 533)** showed moderate inhibition of *P. lividus* eggs division (IC_50_ 9.80, 11.30 µM) and completely inactive with *S. granularis* eggs [235]. Authors’ suggesting that anti-proliferative activity on sea urchin eggs was due to methoxyconidiol and not due to one of its degradation products. The results of this study confirmed previous research finding on sea urchin eggs. Additionally, epiconicol (**77**) was showed most potent activity with human carcinoma cells and had shown poor cytotoxicity against serum stimulated carcinoma cells. Authors confirmed from the study, the position alteration of compound is reducing anti-bacterial and anti-proliferative activity of meroterpenes on sea urchin eggs and on normal, immortalized and cancer cell lines, displaying the vital role of the phenolic group at the C-4 position for increase the cytotoxicity of meroterpenes derivatives.

Furthermore, lipopeptide 39-oxobistramide K 988 (**535**) with known bistramides A, D (**536, 537**) were isolated from *Trididemnum cyclops* collected in Madagascar [237]. Compound (**535**) showed potent cytotoxicity against A2780 (IC_50_ 0.34 µM). Pyrrole alkaloids, Lamellarin N (**538**) with nine sulphate derivatives, lamellarins T, U, V and Y (**539**–**542**) and lamellarins T–X (**543**–**547**) were isolated from the unidentified ascidian collected in Arabian Sea, India [238]. Lamellarin N (**538**) showed cytotoxicity against melanoma cell lines SK-MEL-5 (LC_50_ 1.87 µM) and with UACC-62 (LC_50_ 9.88 µM), respectively.

Two new cyclic heptapeptides, mayotamides A and B (**548, 549**) and known cyclic hexapeptides, comoramides A and B (**550**–**551**) were reported from the ascidian *Didemnum molle* collected in Madagascar [239]. All four compounds (**548**–**551**) had shown moderate cytotoxicity against the tumor cells (A549, HT29 and MEL-28) with IC_50_ 5–10 µg/mL. New sulfonated serinol derivatives, siladenoserinols A–L (**552**–**563**) were isolated from a tunicate of the family Didemnidae. All the compounds showed moderate inhibition against p53-Hdm2 inhibitor IC_50_ 2.0–55 µM. Compounds (**552, 553**) showed remarkable most potent inhibition against p53-Hdm2 inhibitor (IC_50_ 2.0 µM) [240]. Targeting Mdm2/Hdm2 is a promising way to reactivate p53, prompting apoptosis in transformed human cells. Reactivation of p53 via this approach is also considered a potential way to cancer-prevention, however this needs further study (Structure 10).

**Structure 10:**
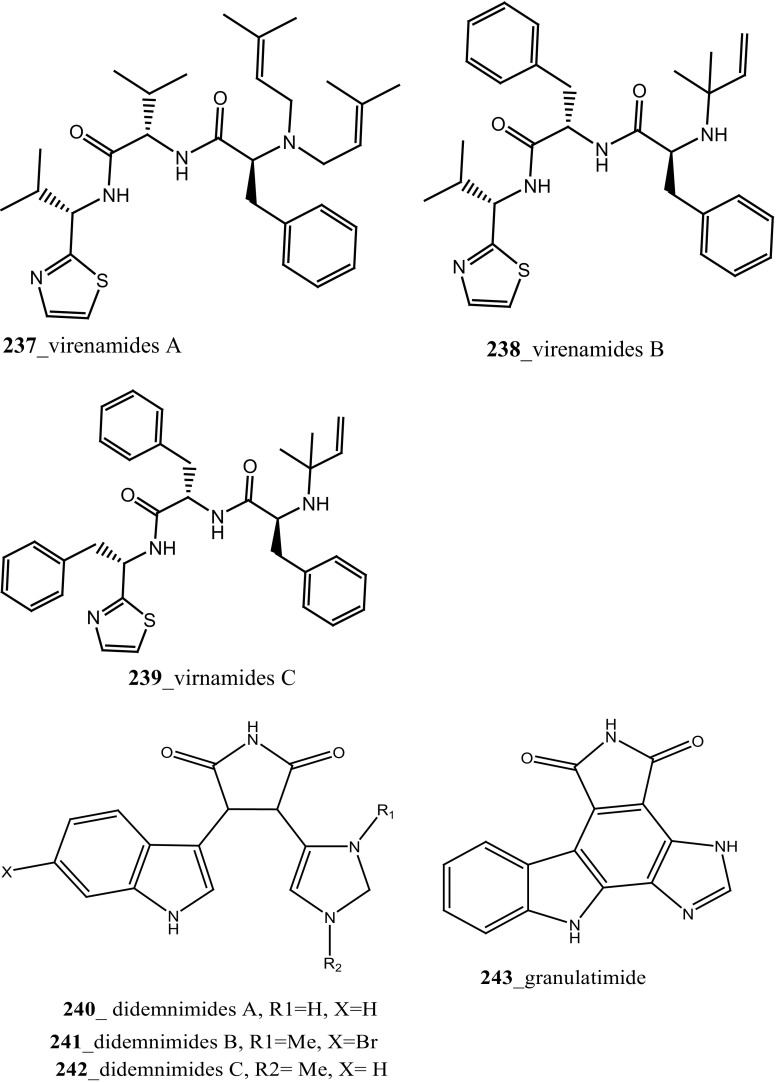

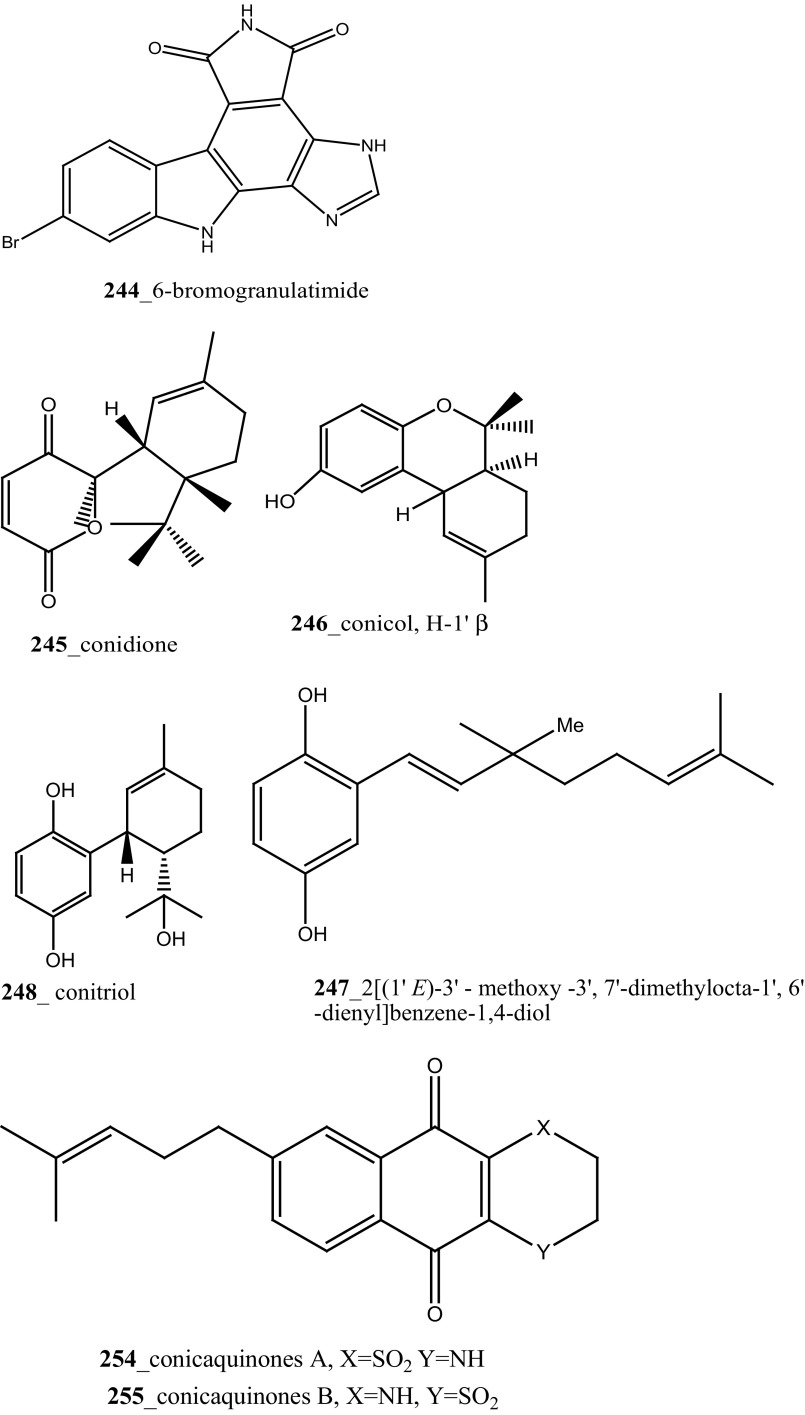

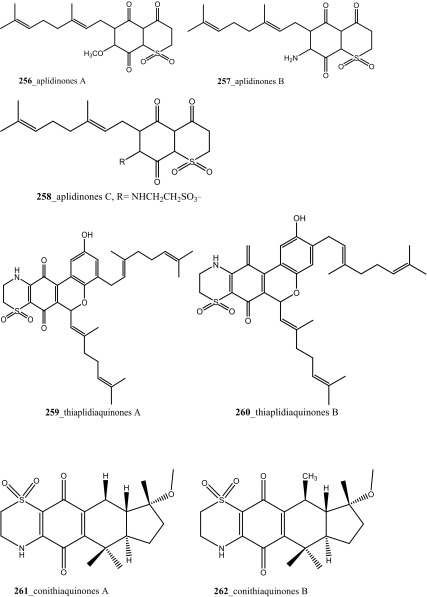

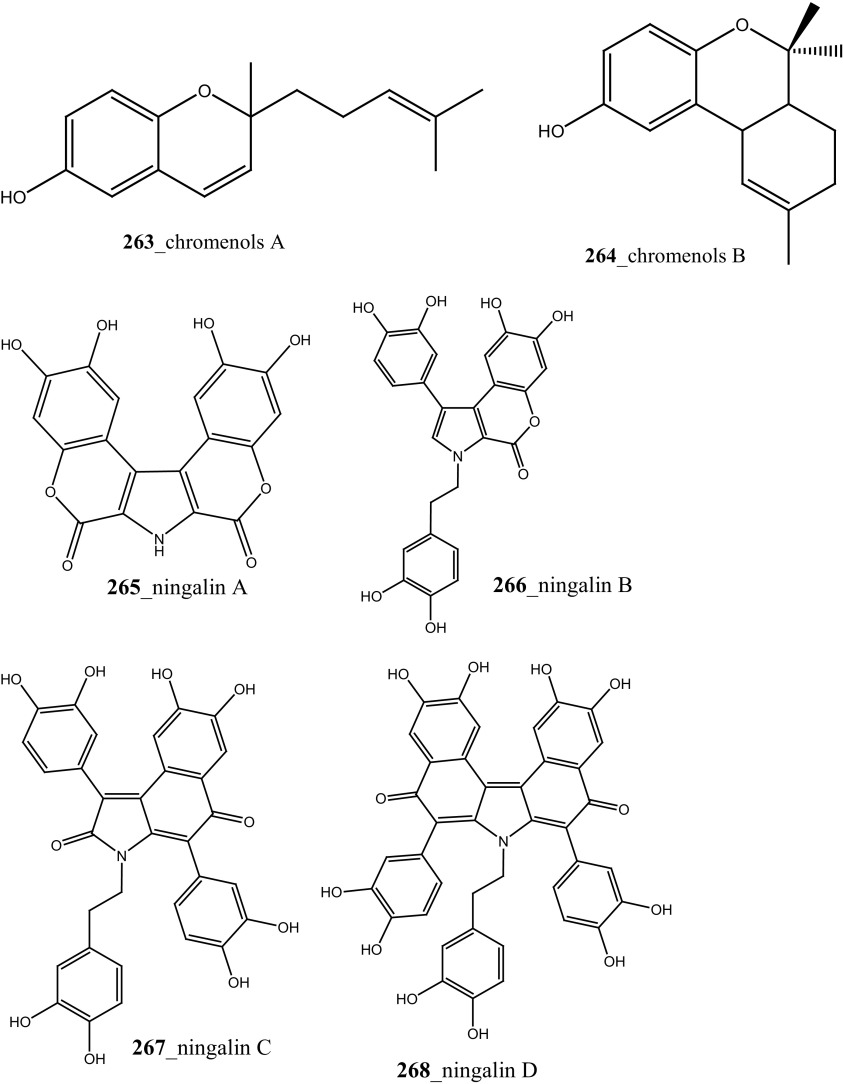

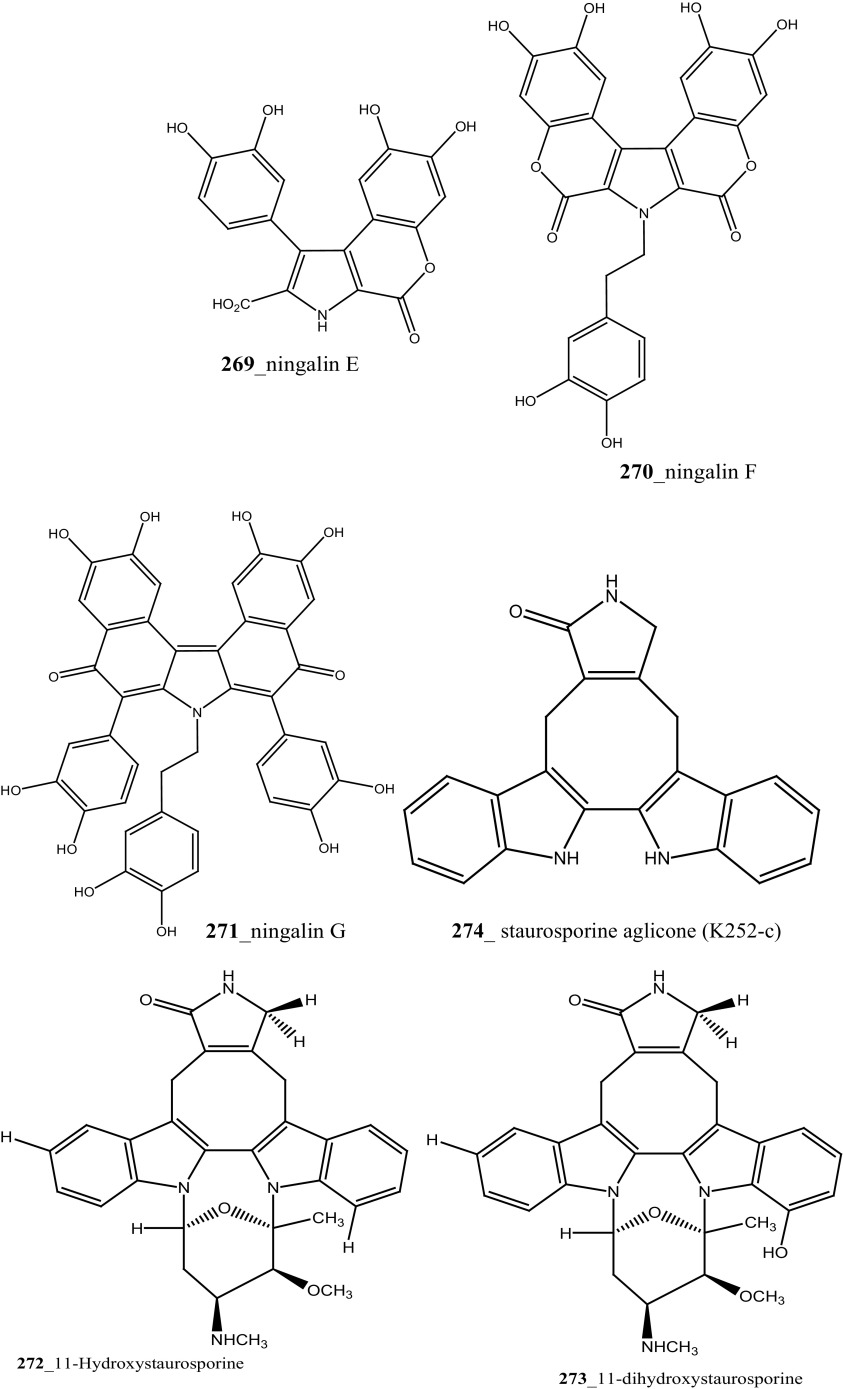

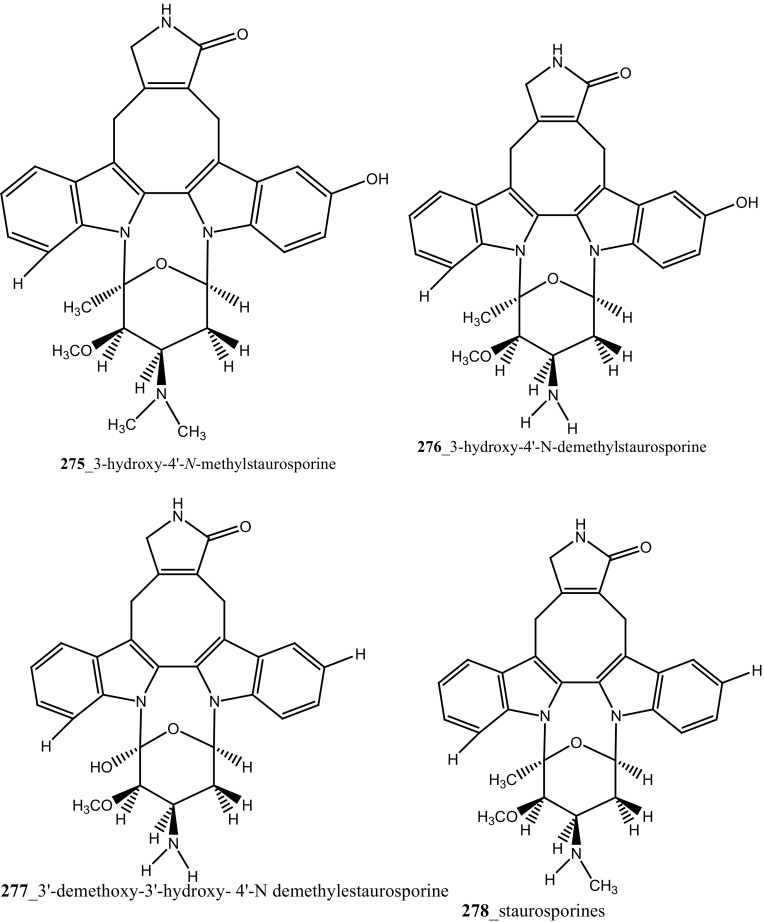

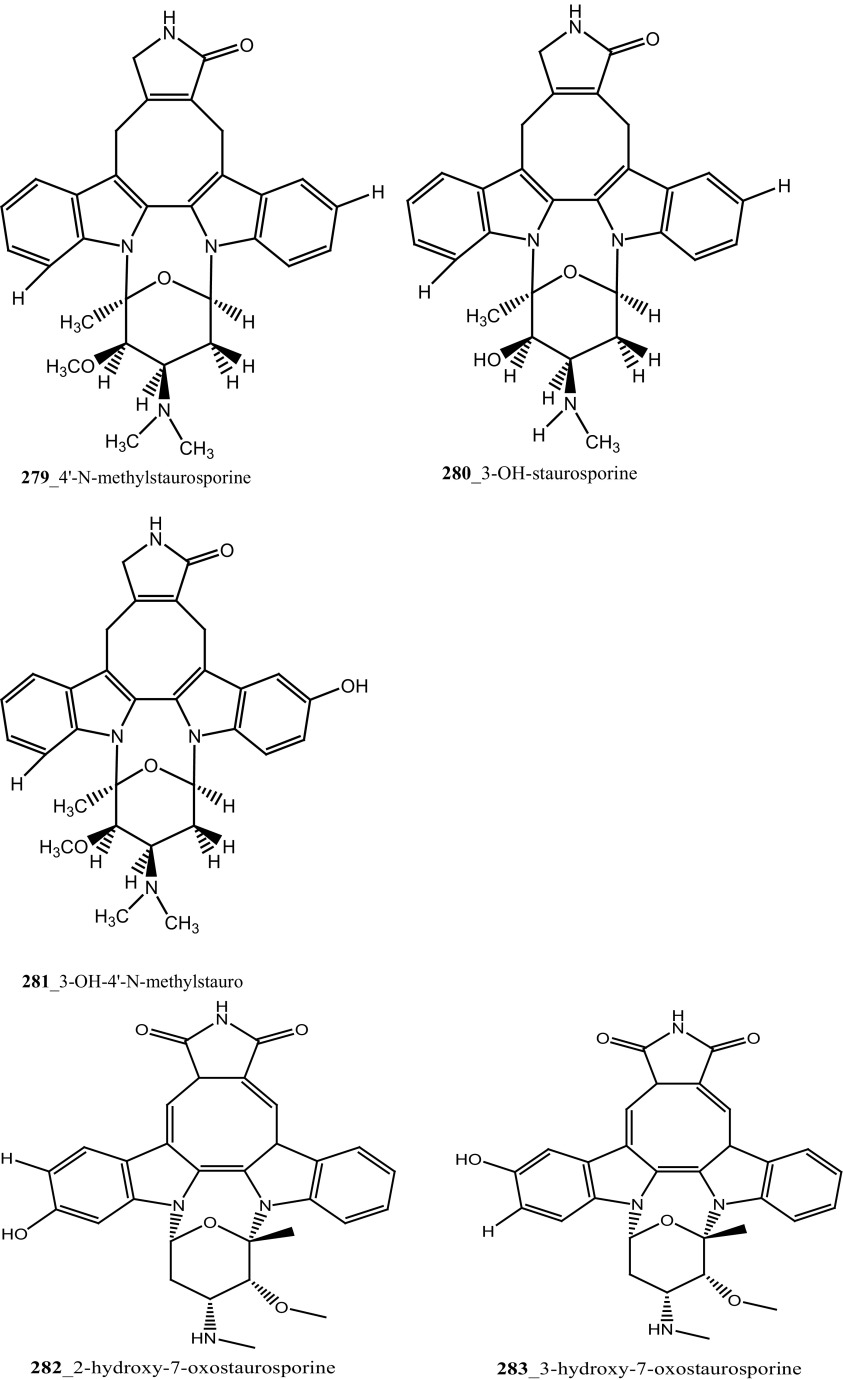

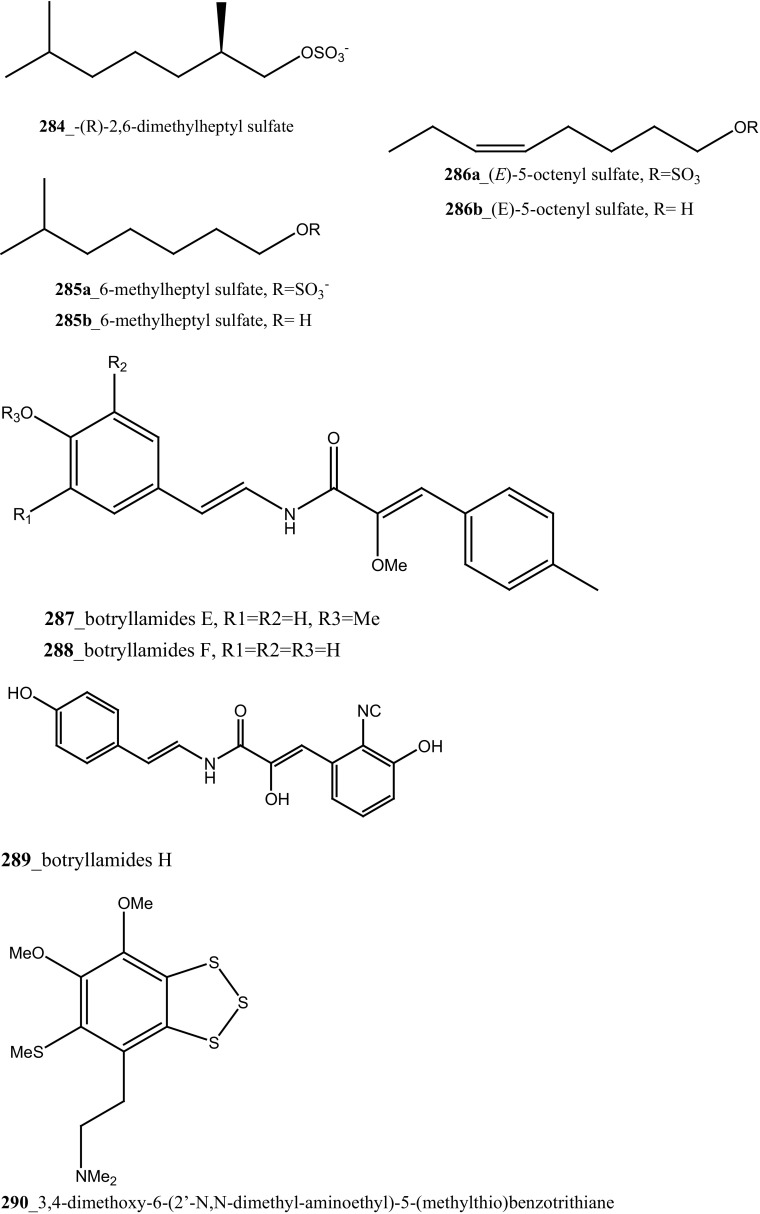

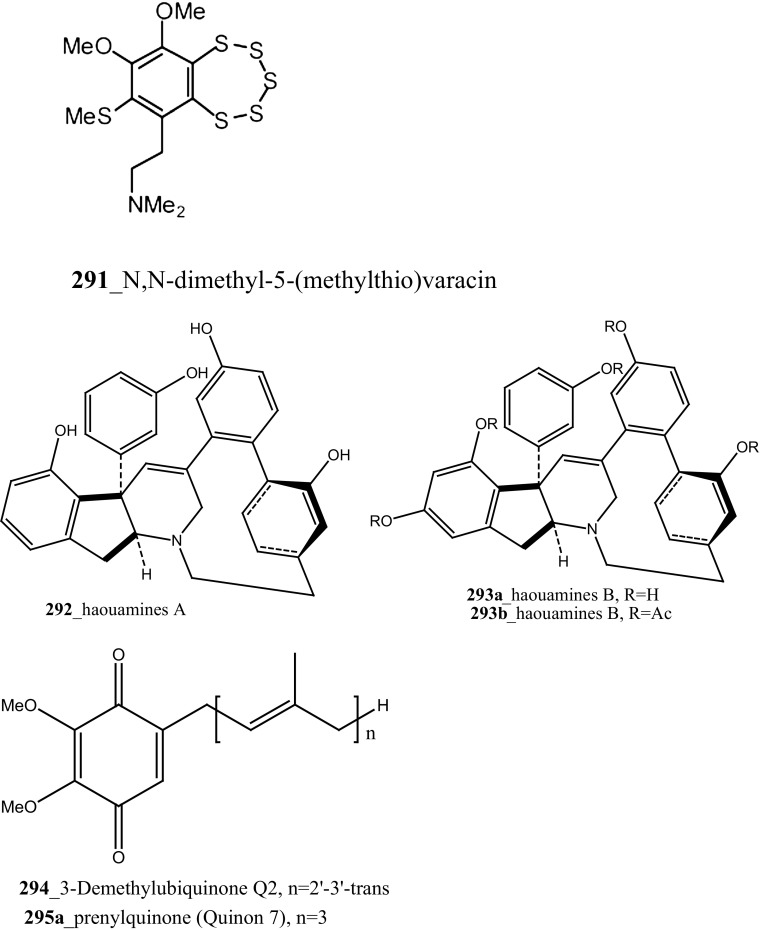

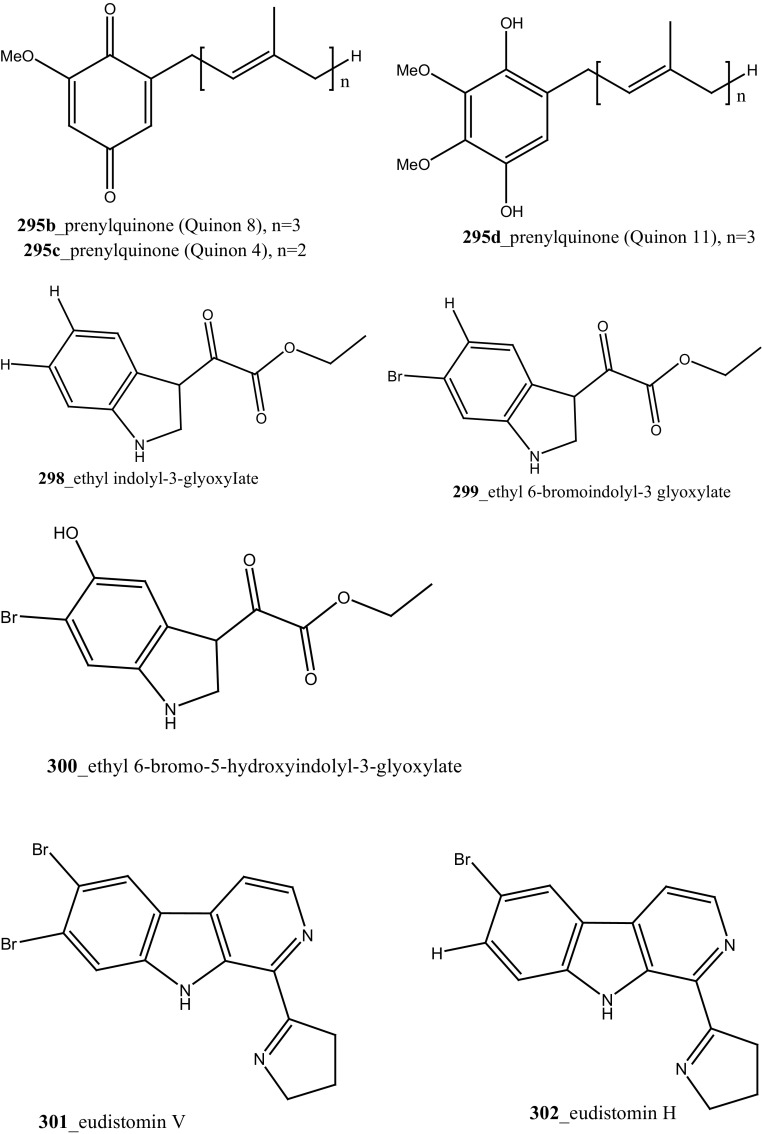

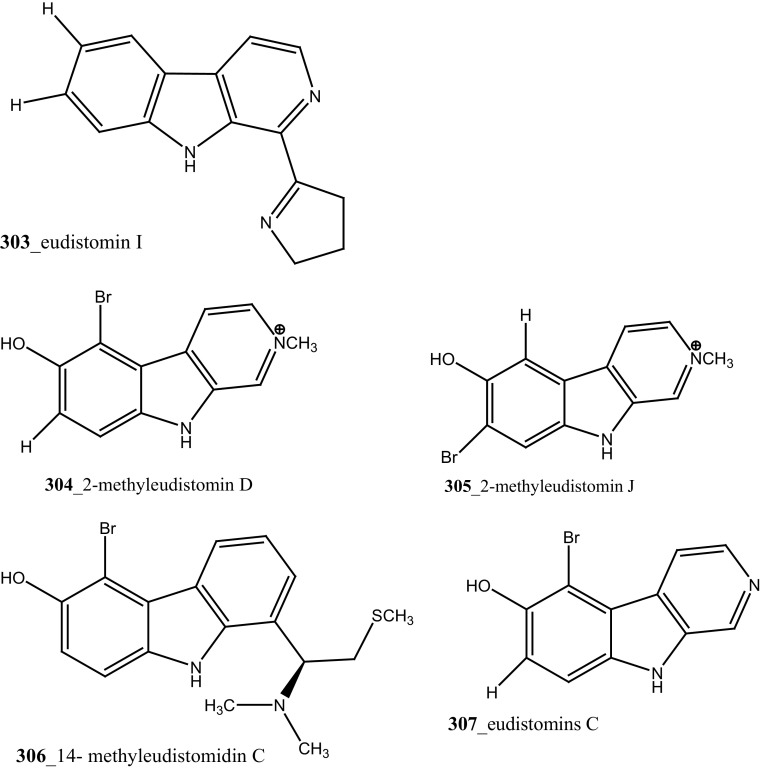

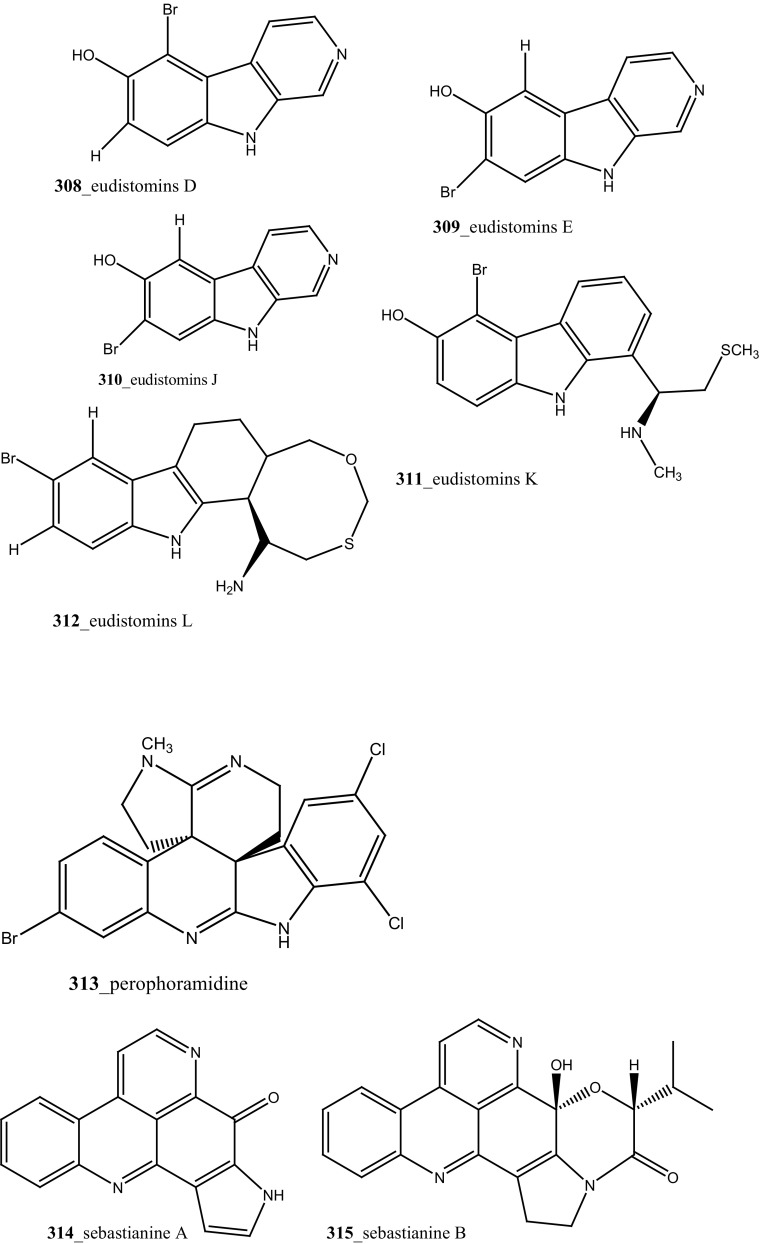

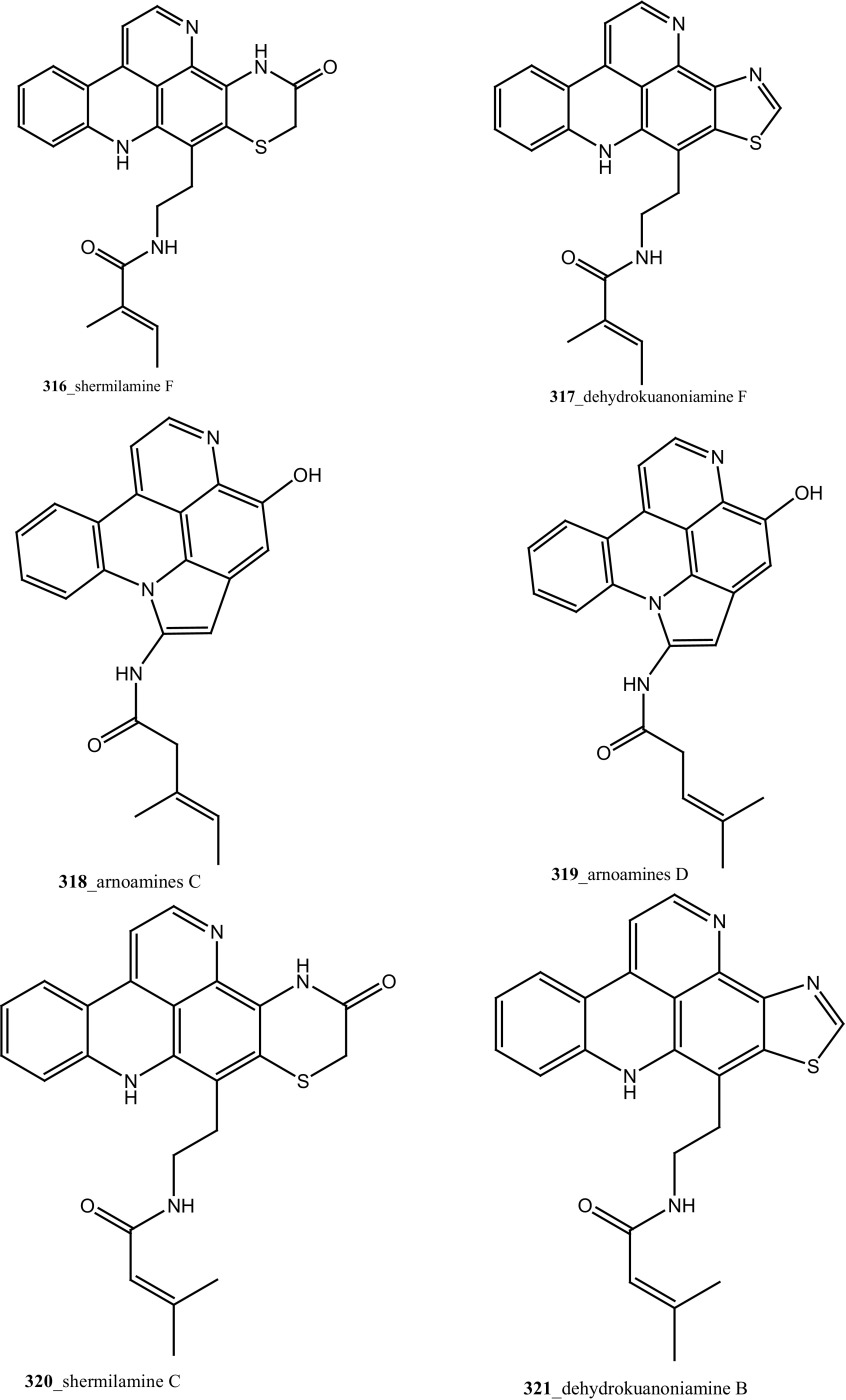

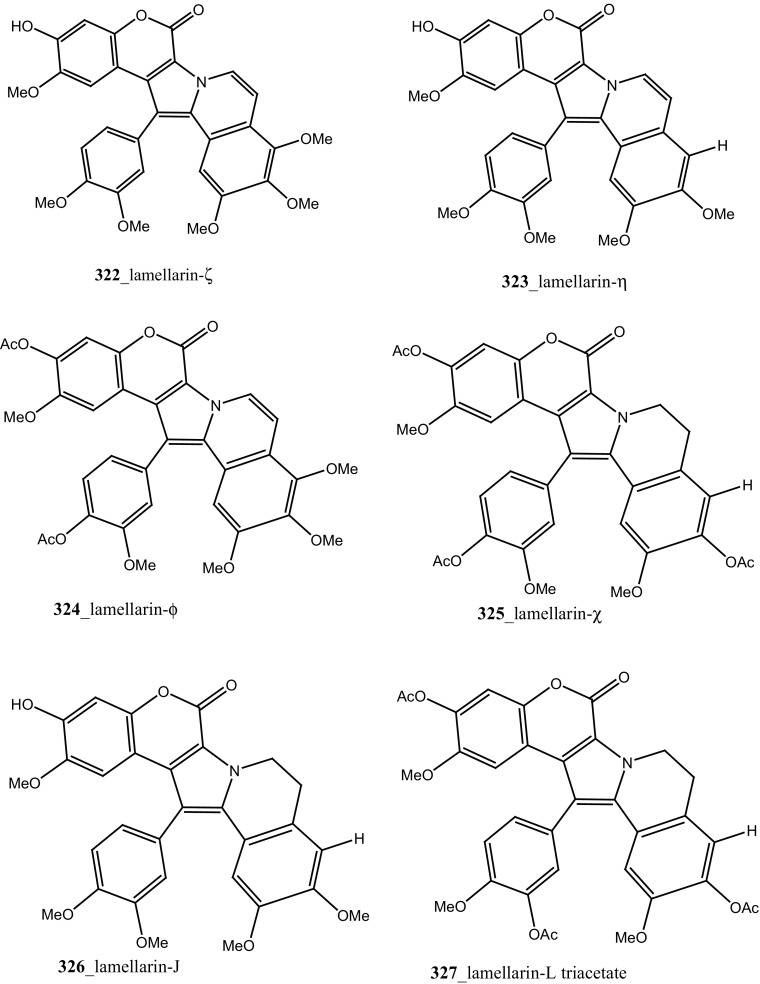

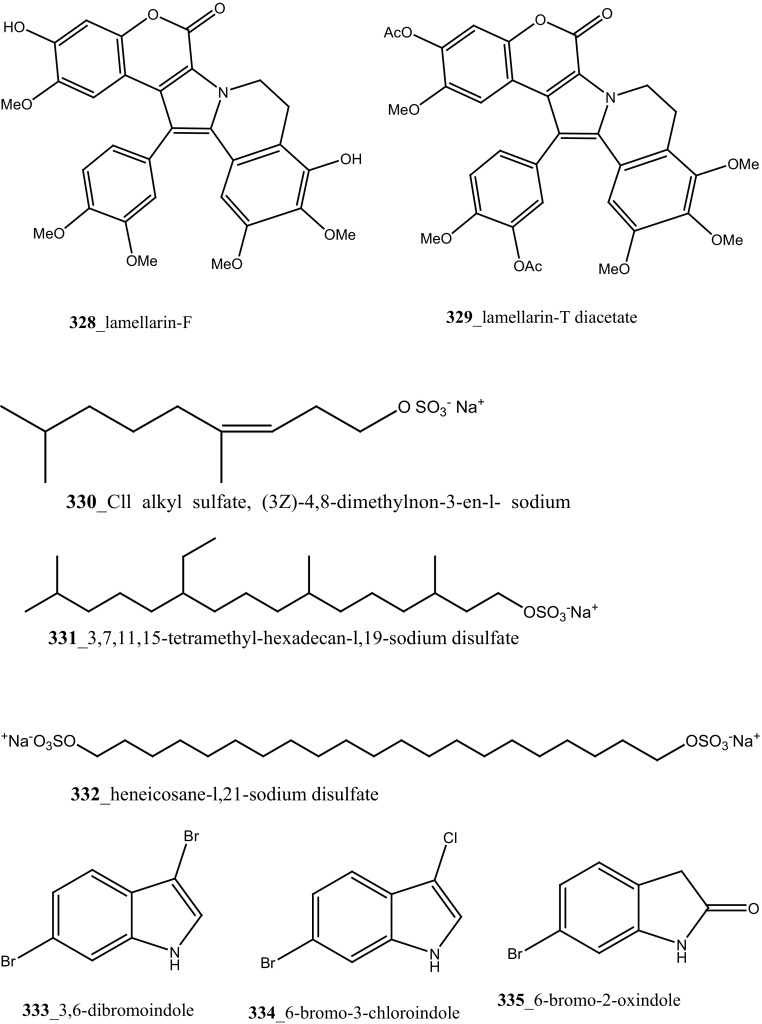

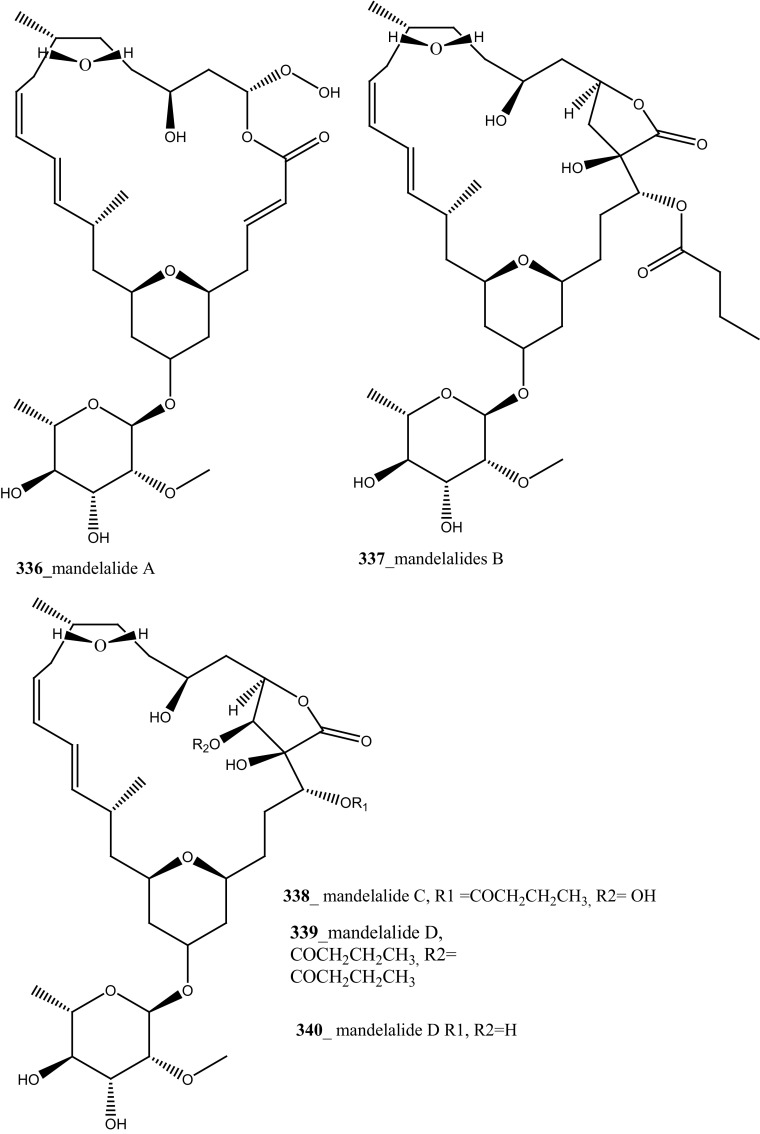

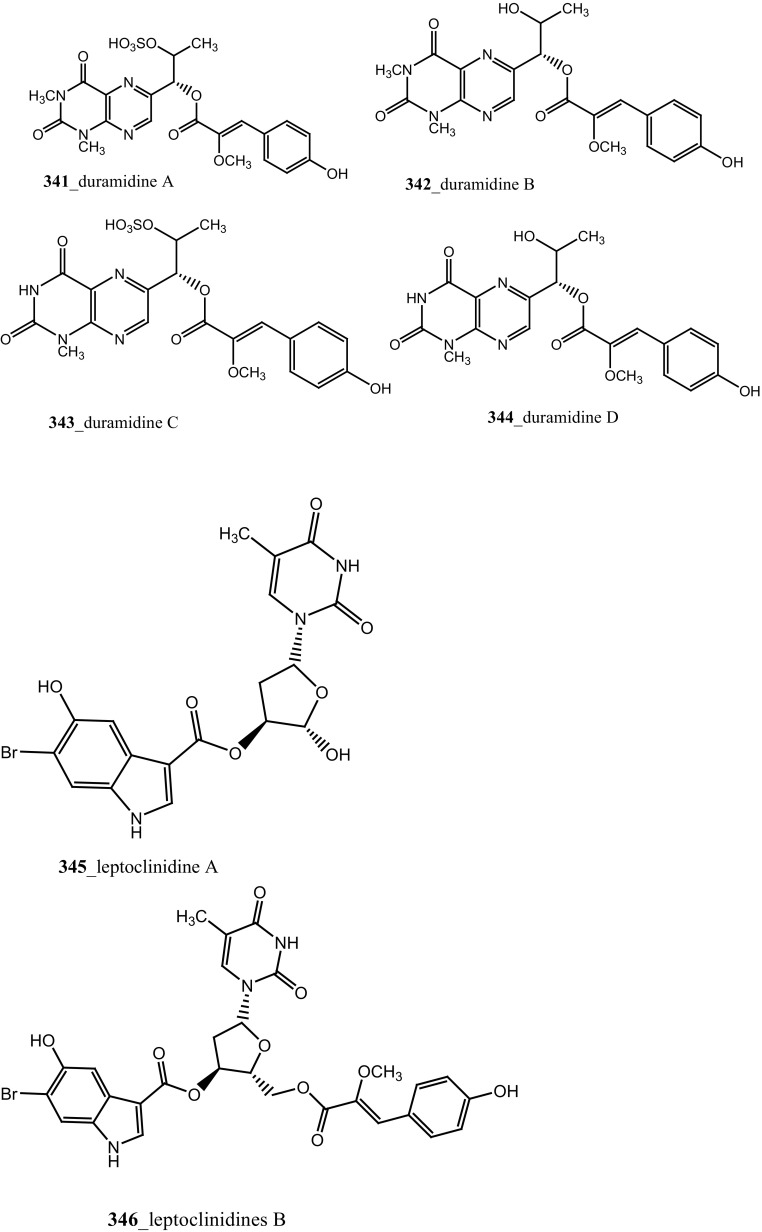

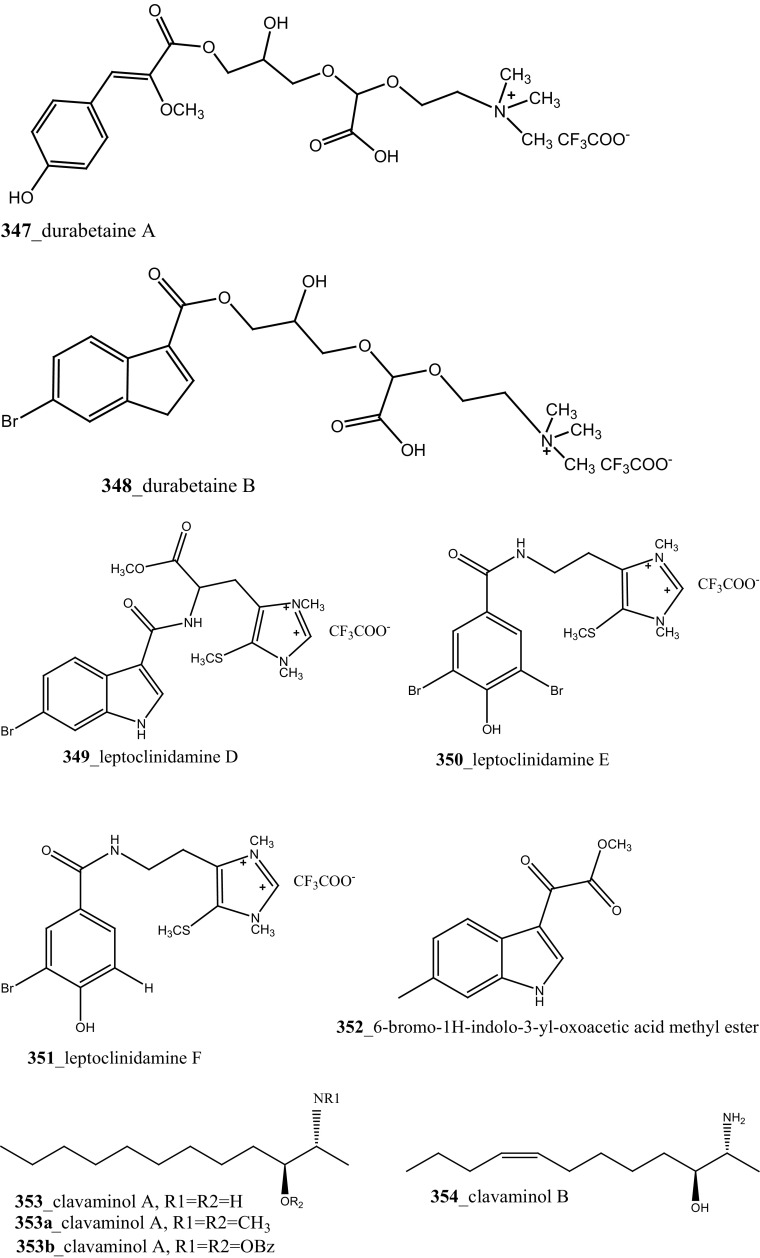

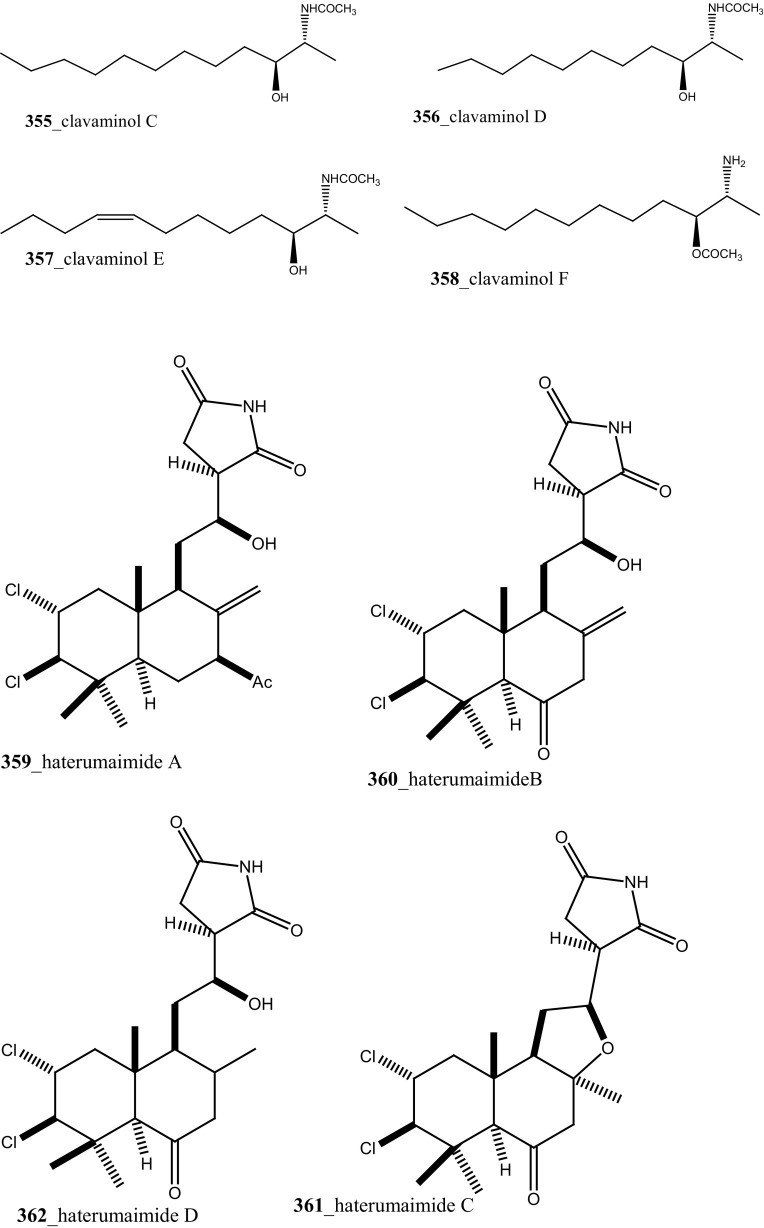

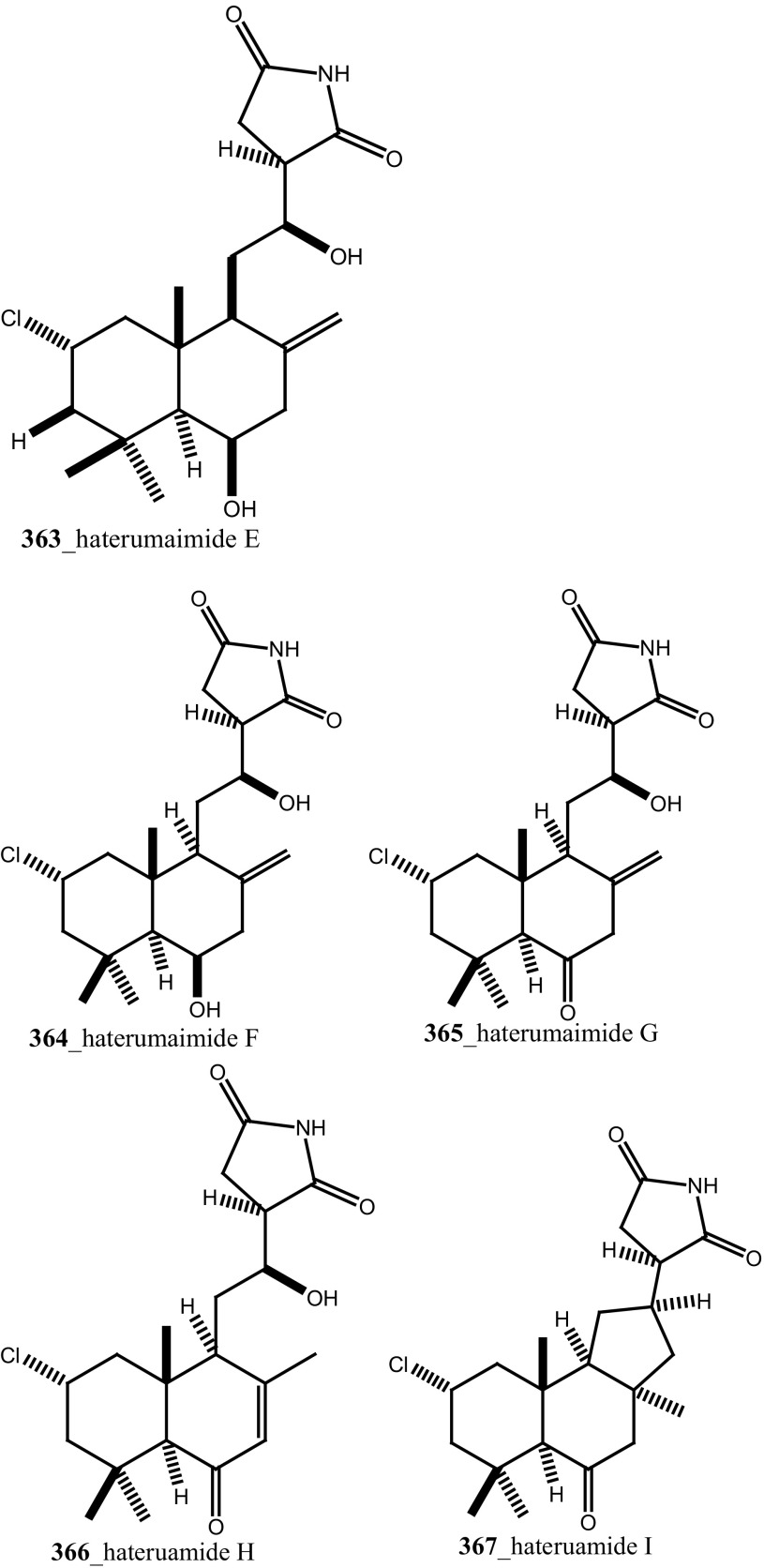

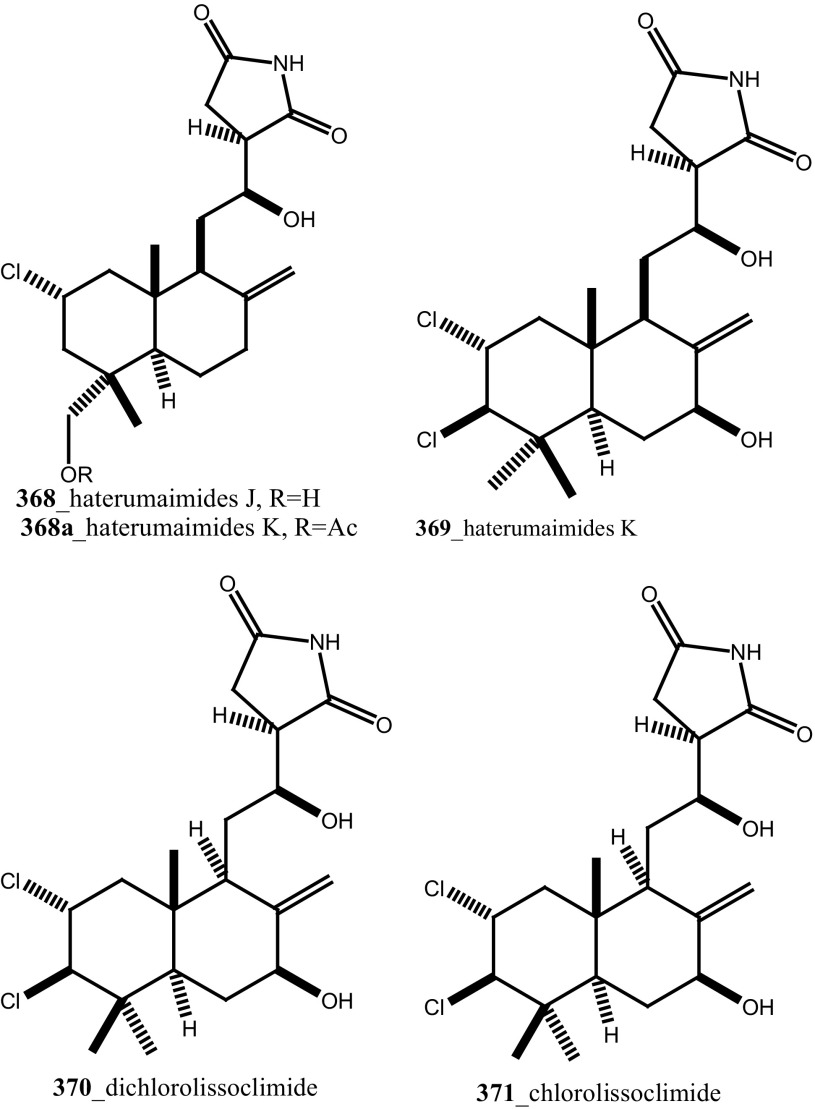

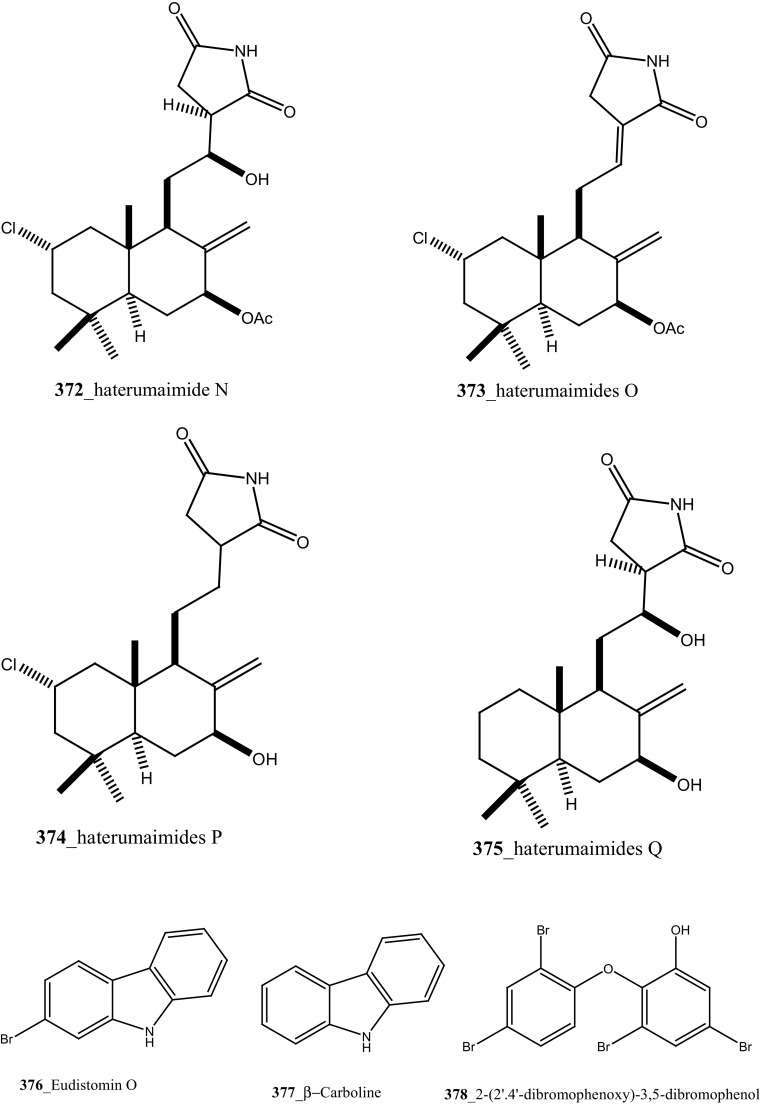

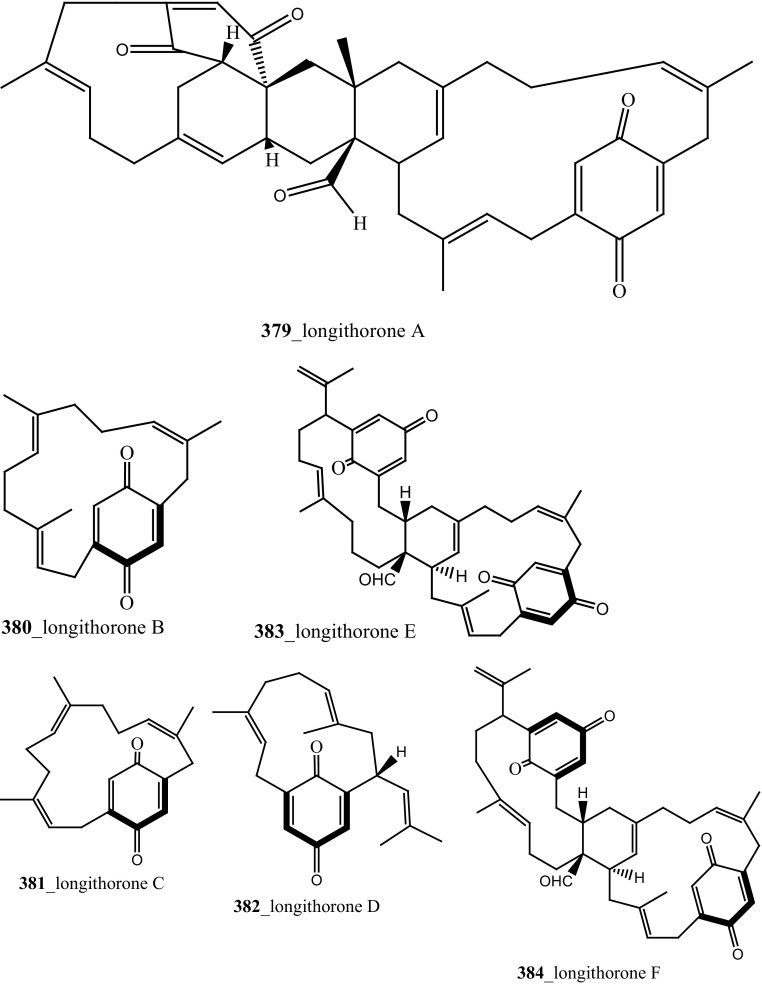

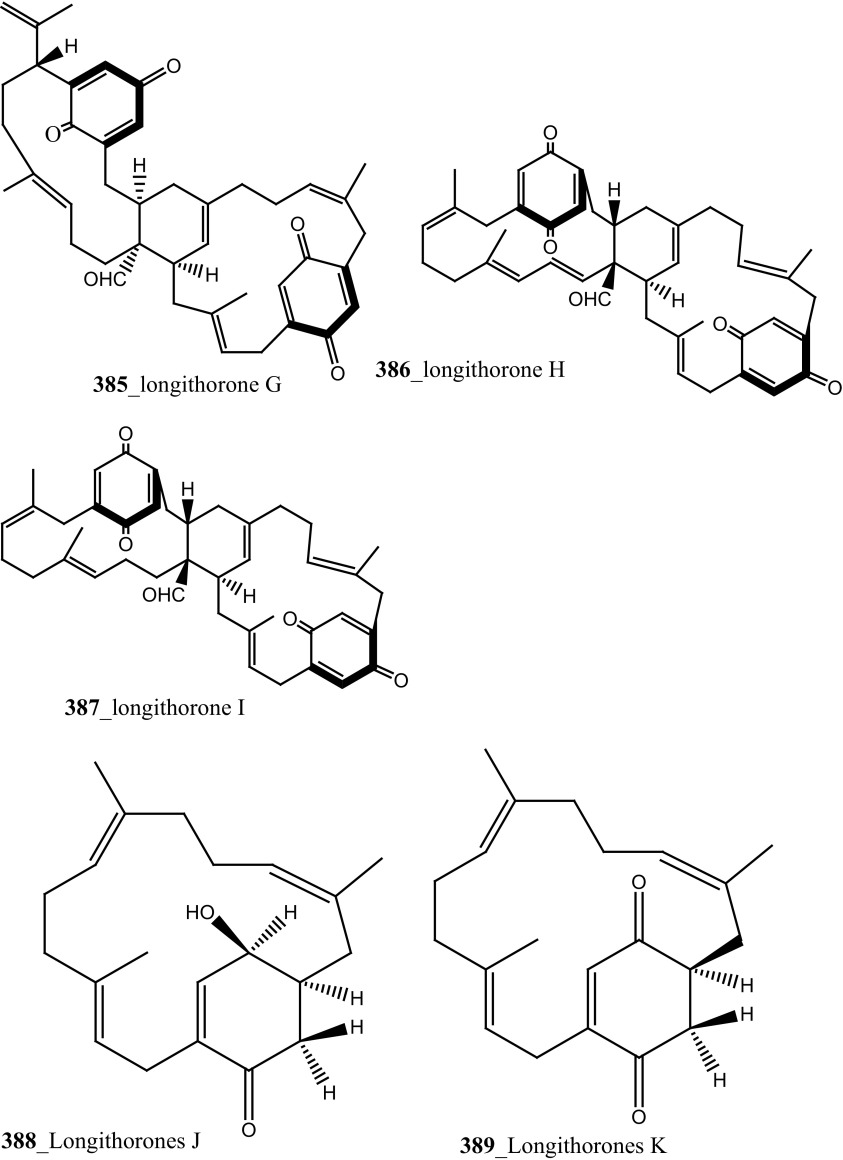

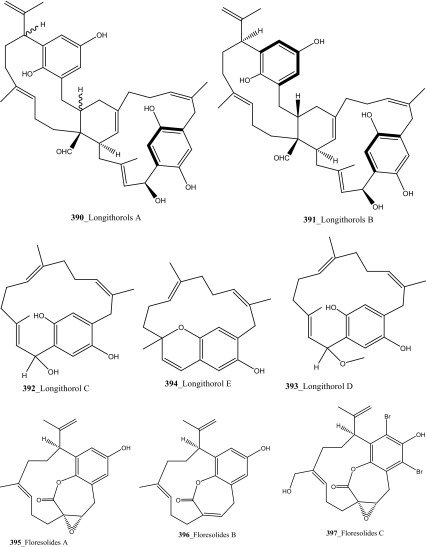

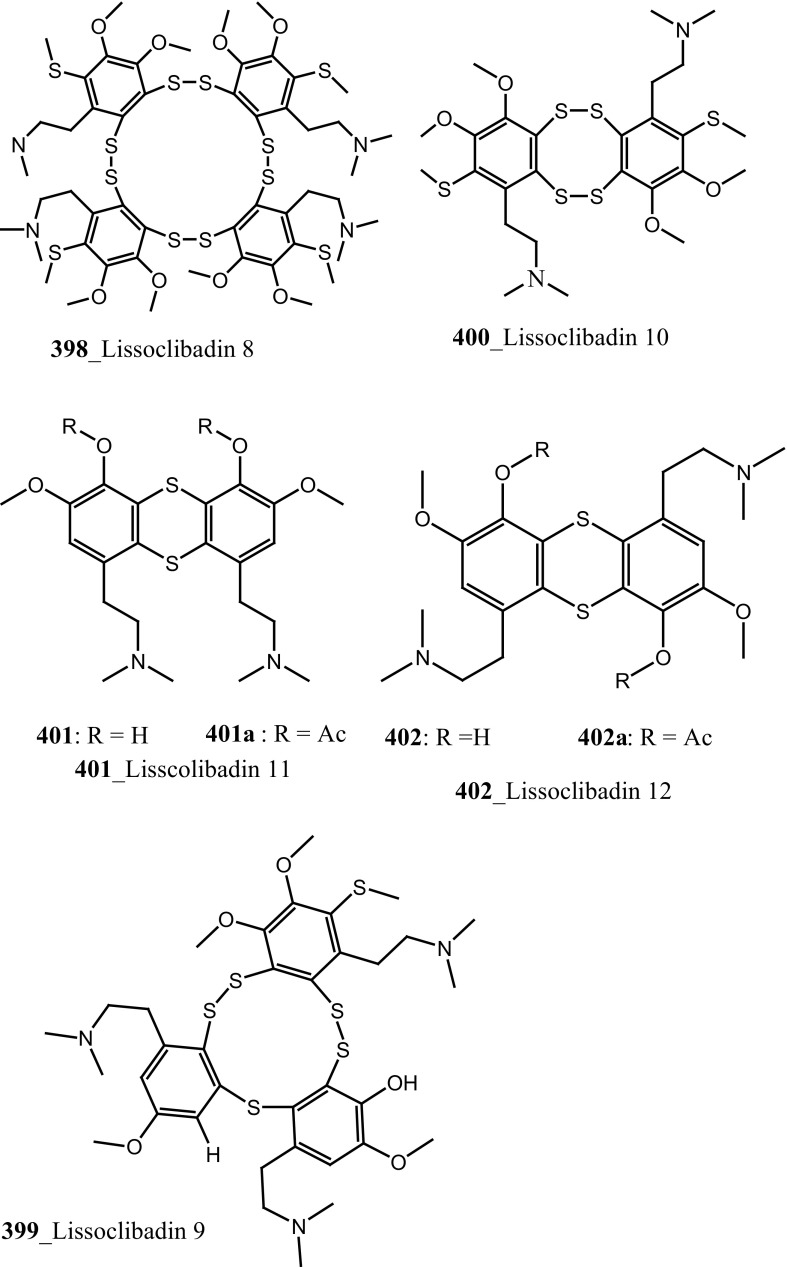

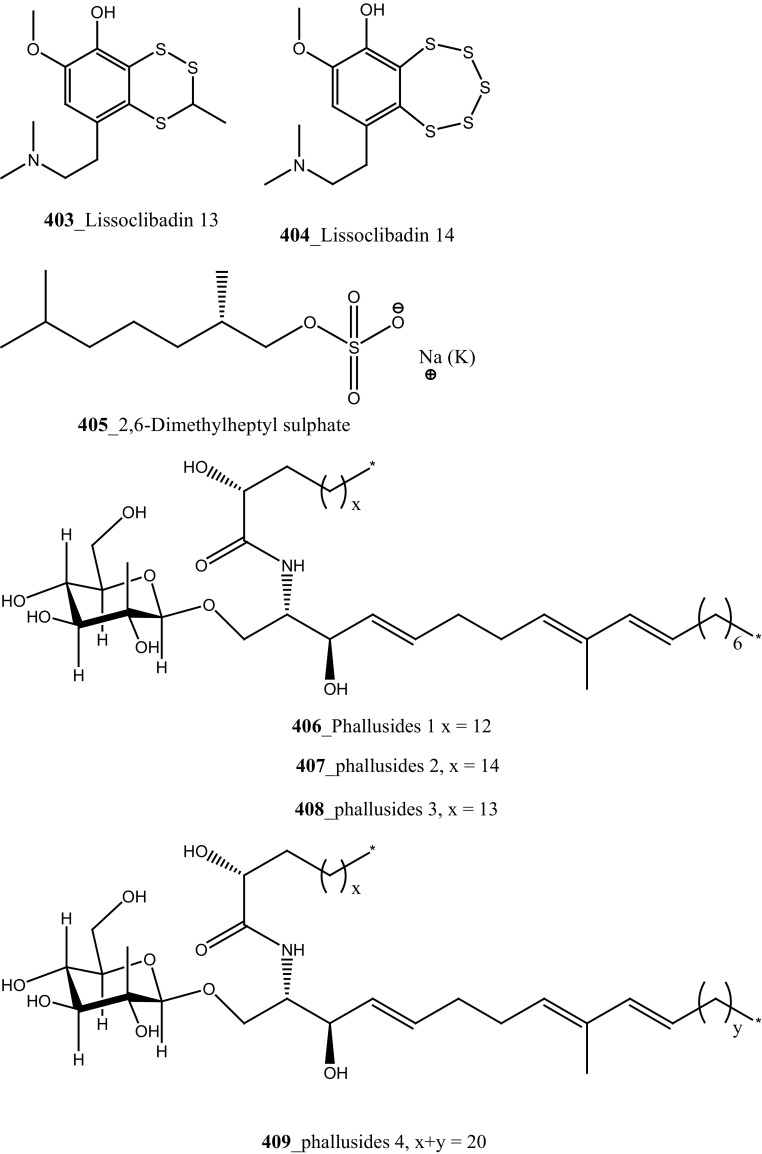

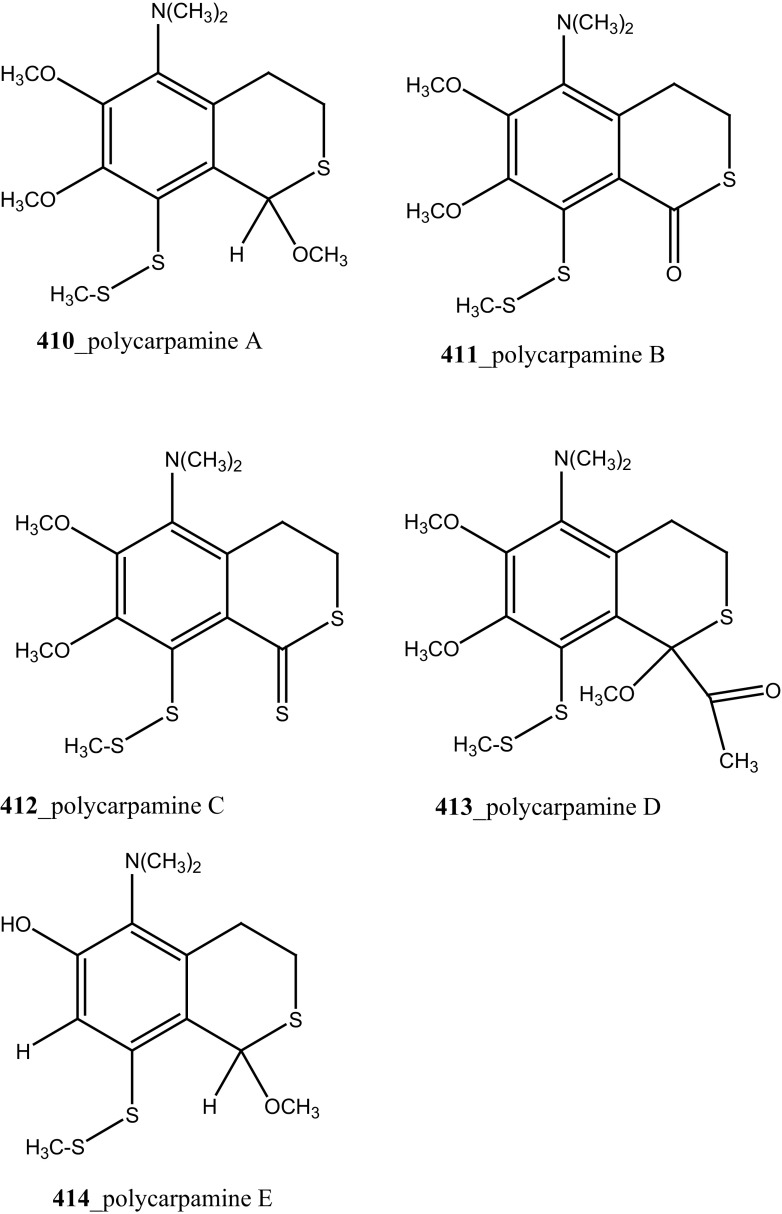

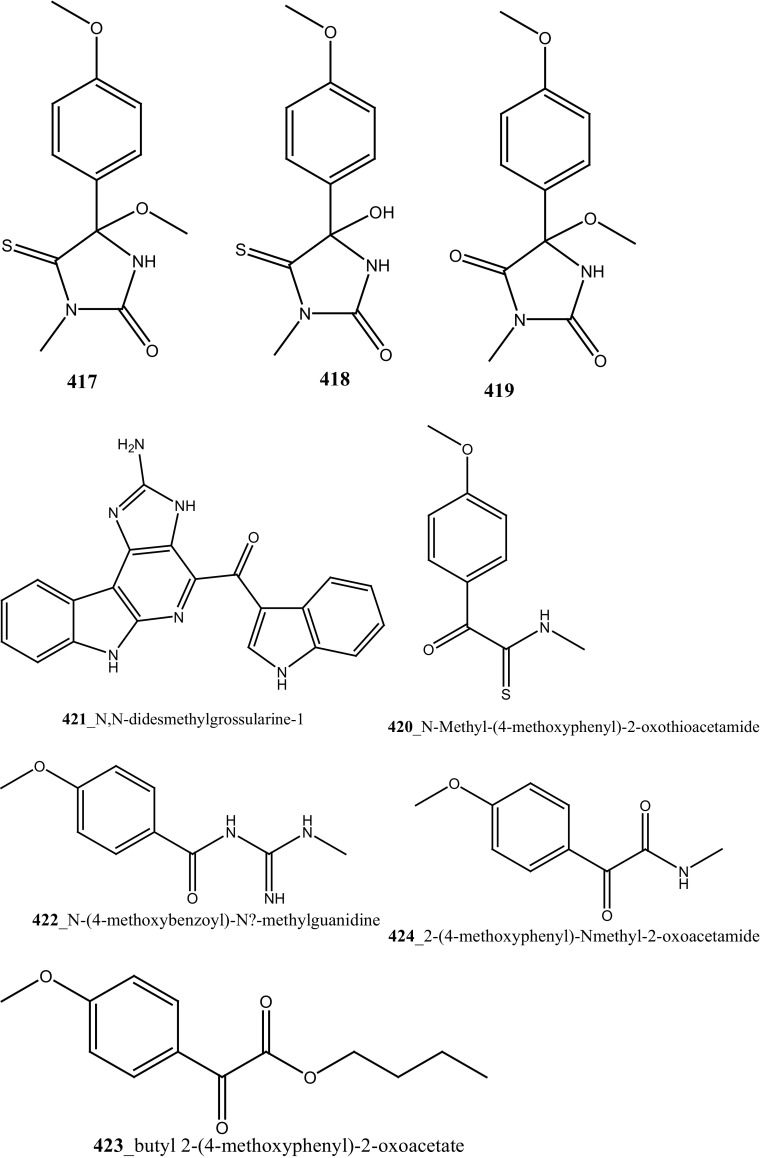

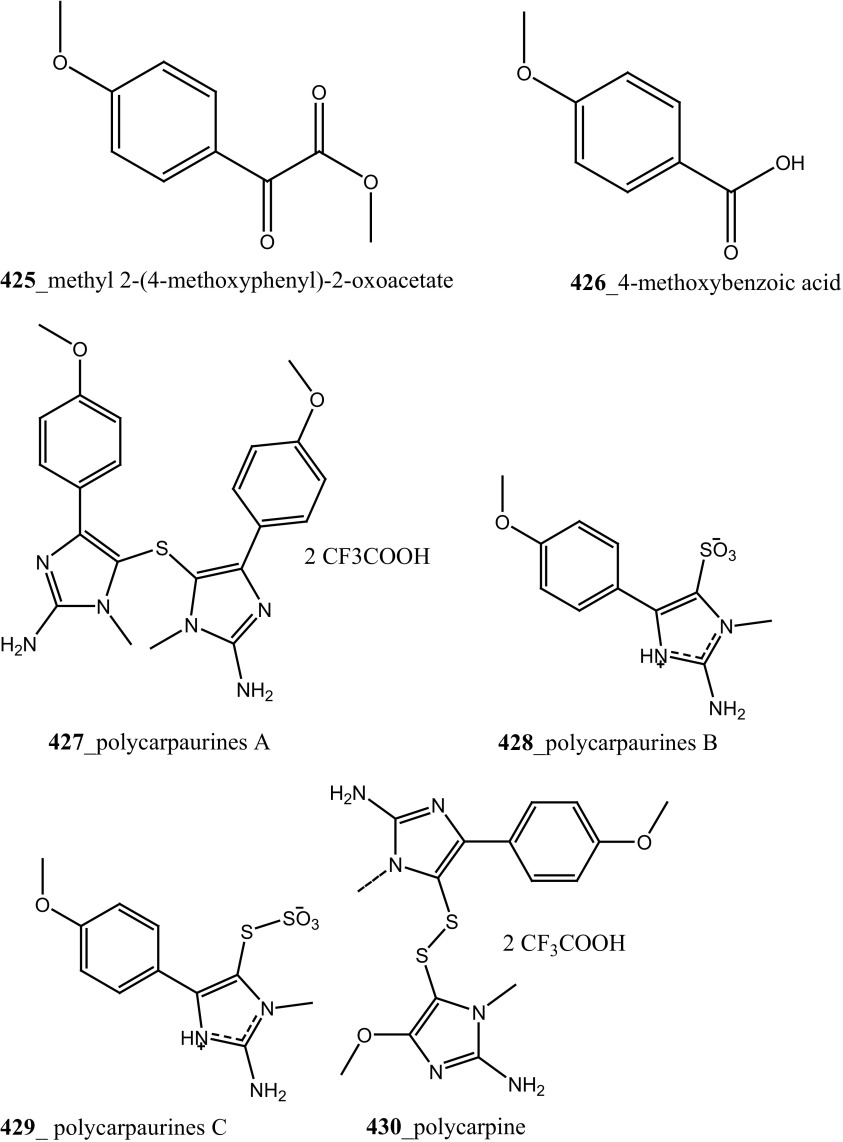

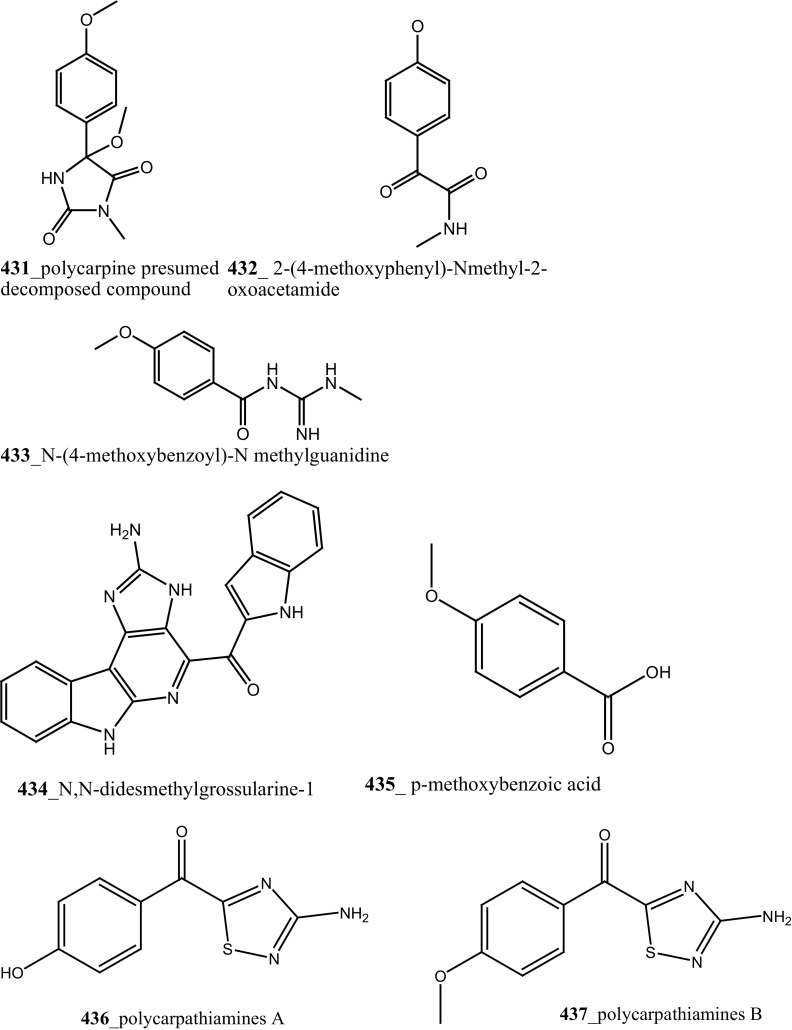

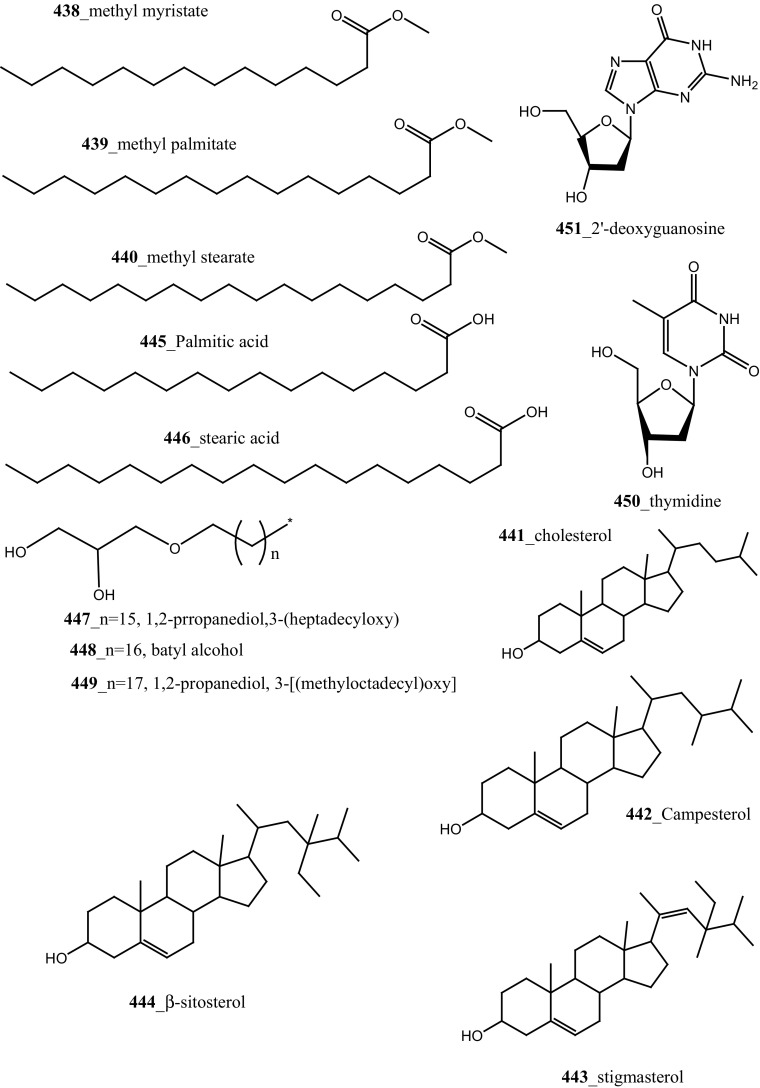

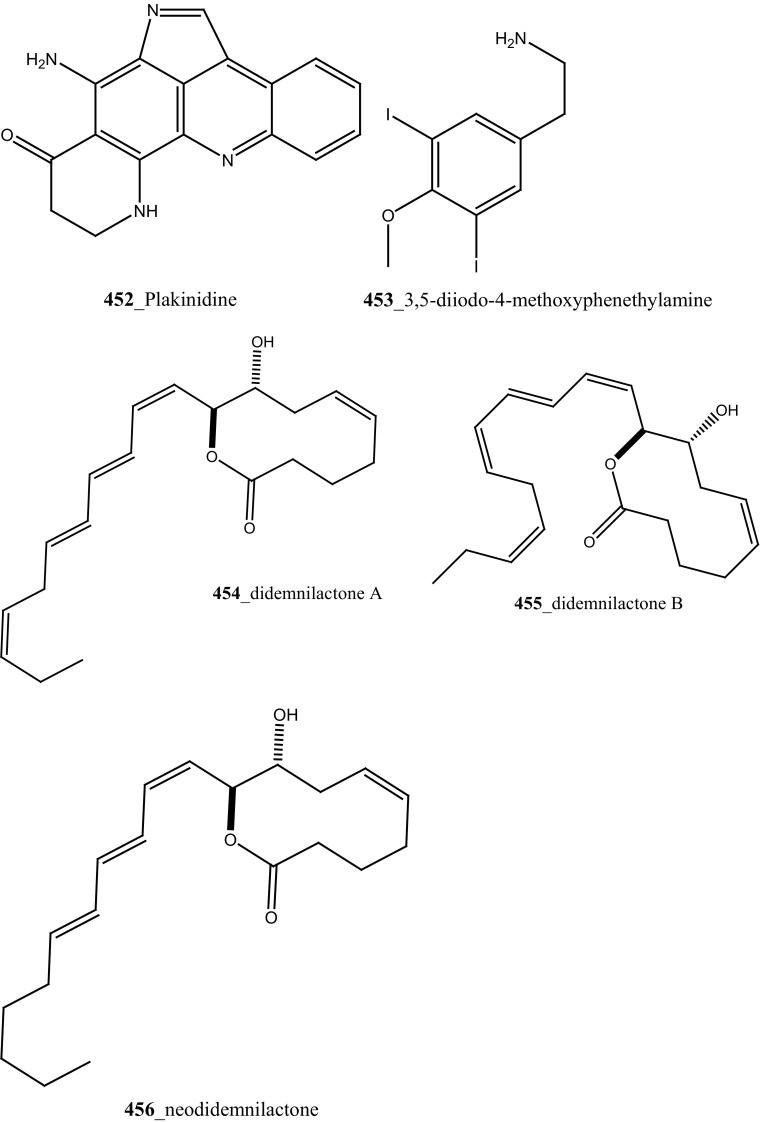

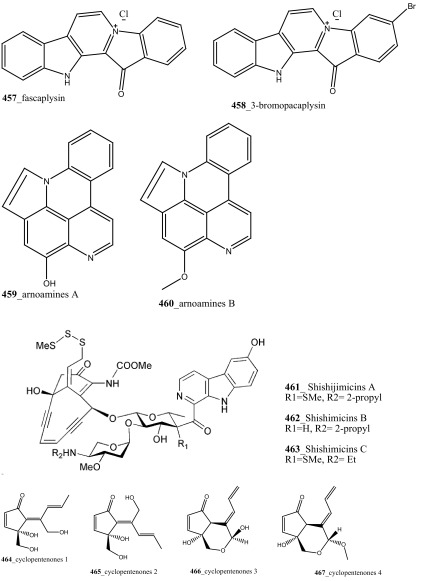

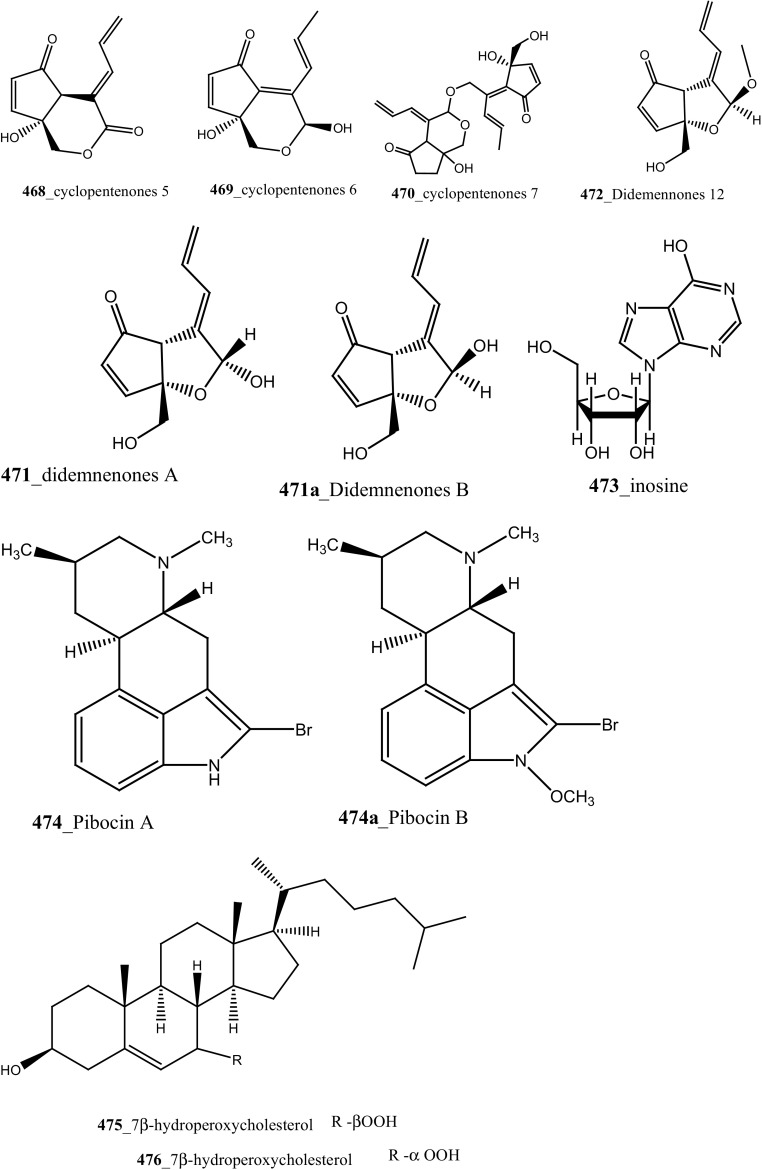

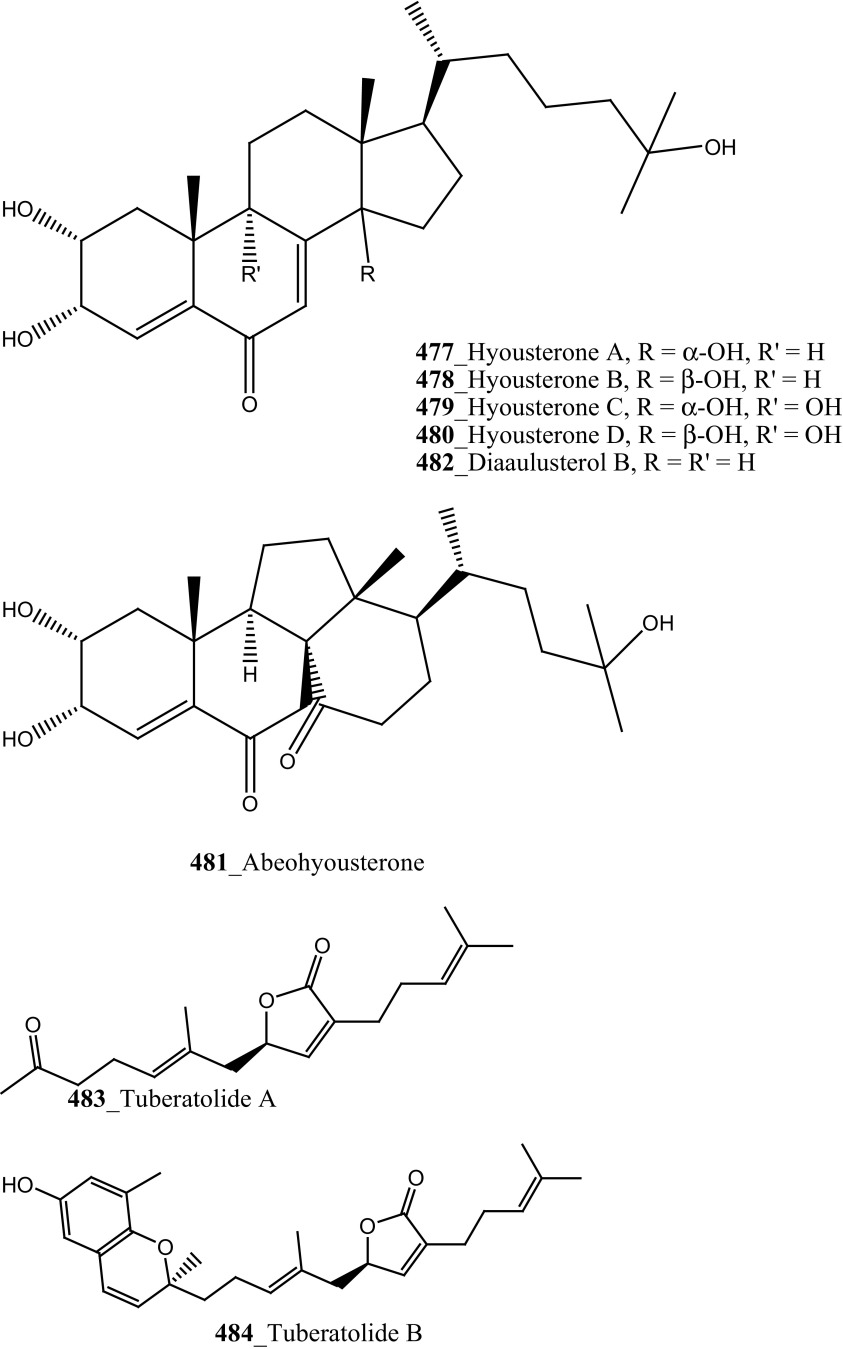

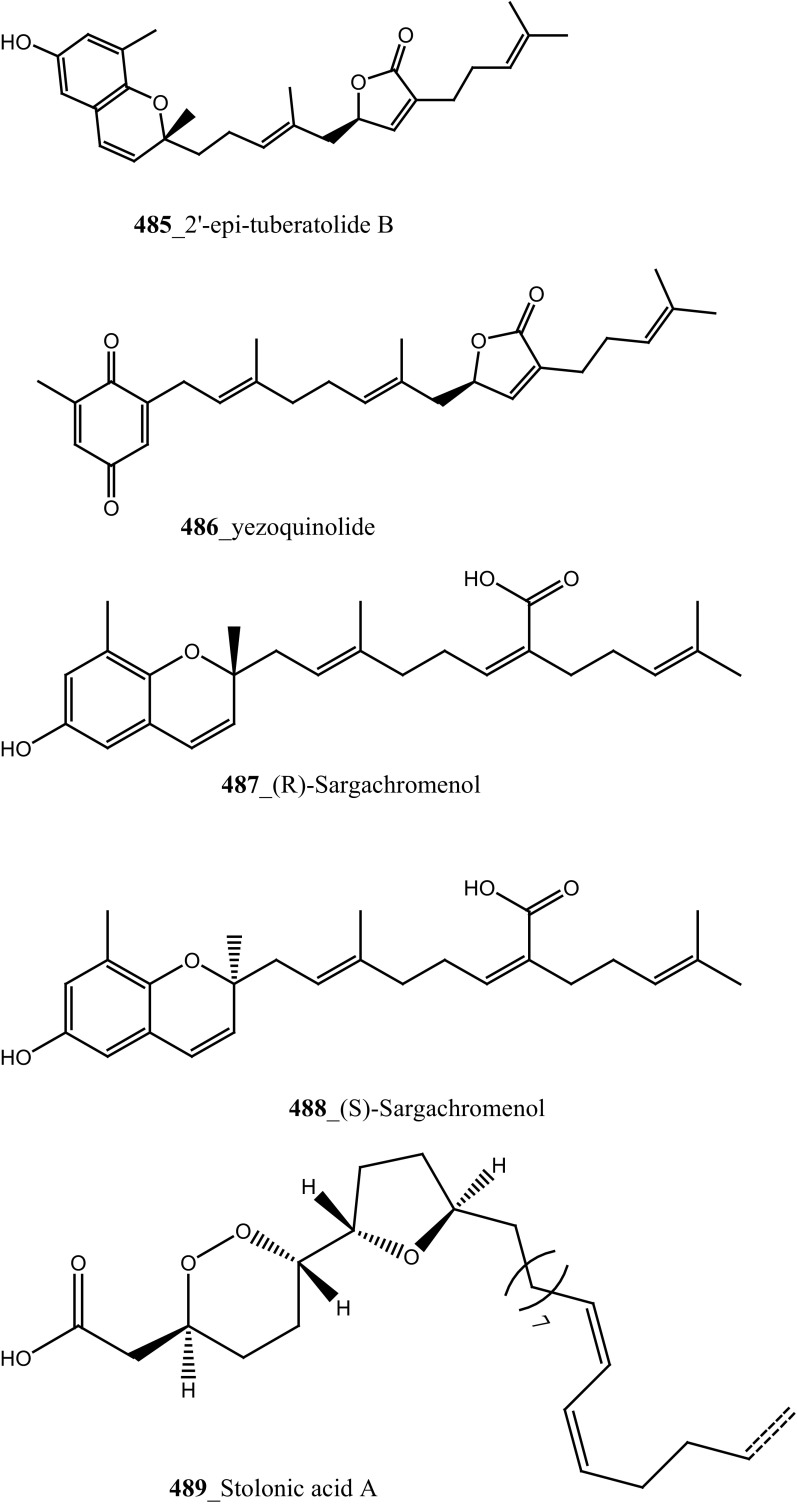

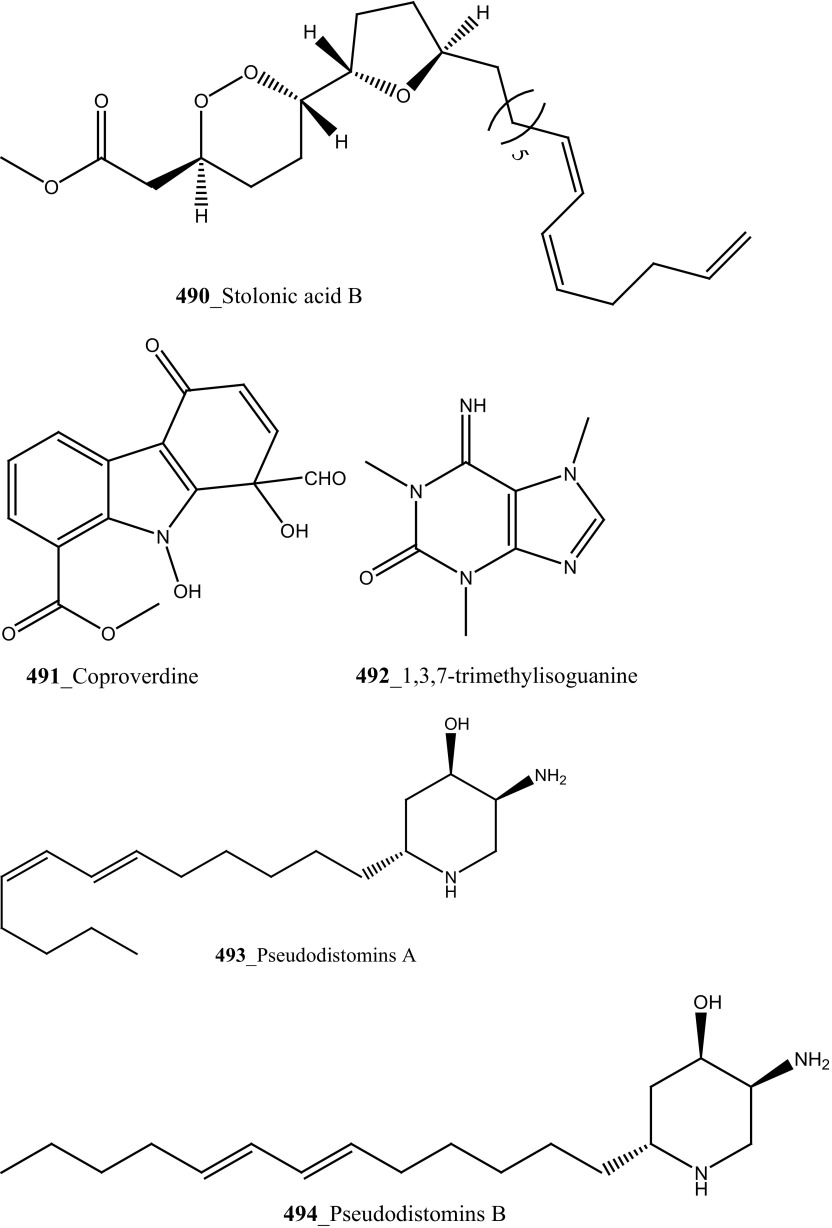

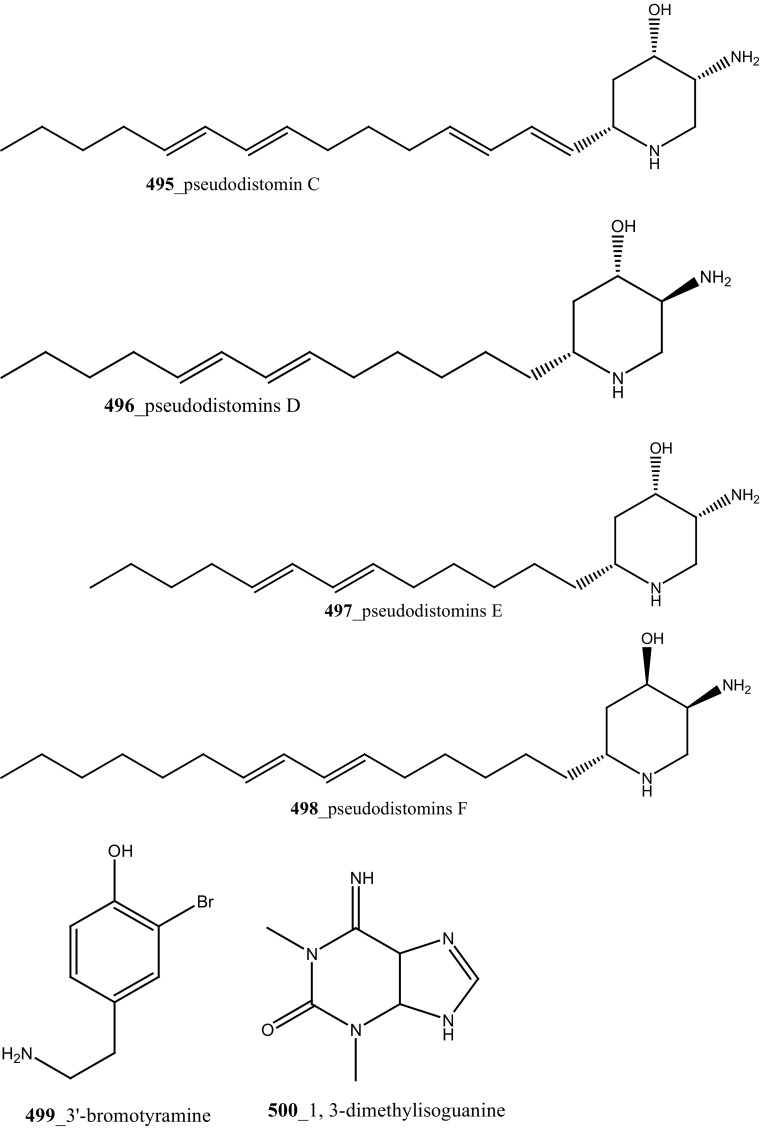

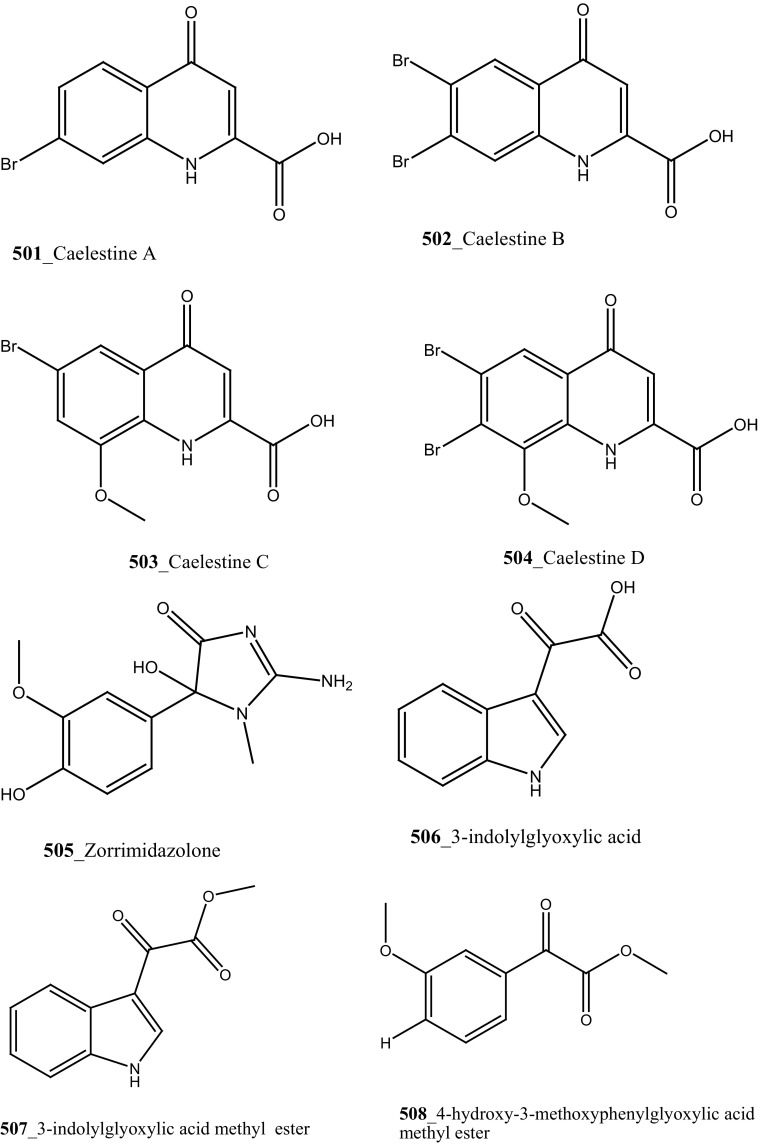

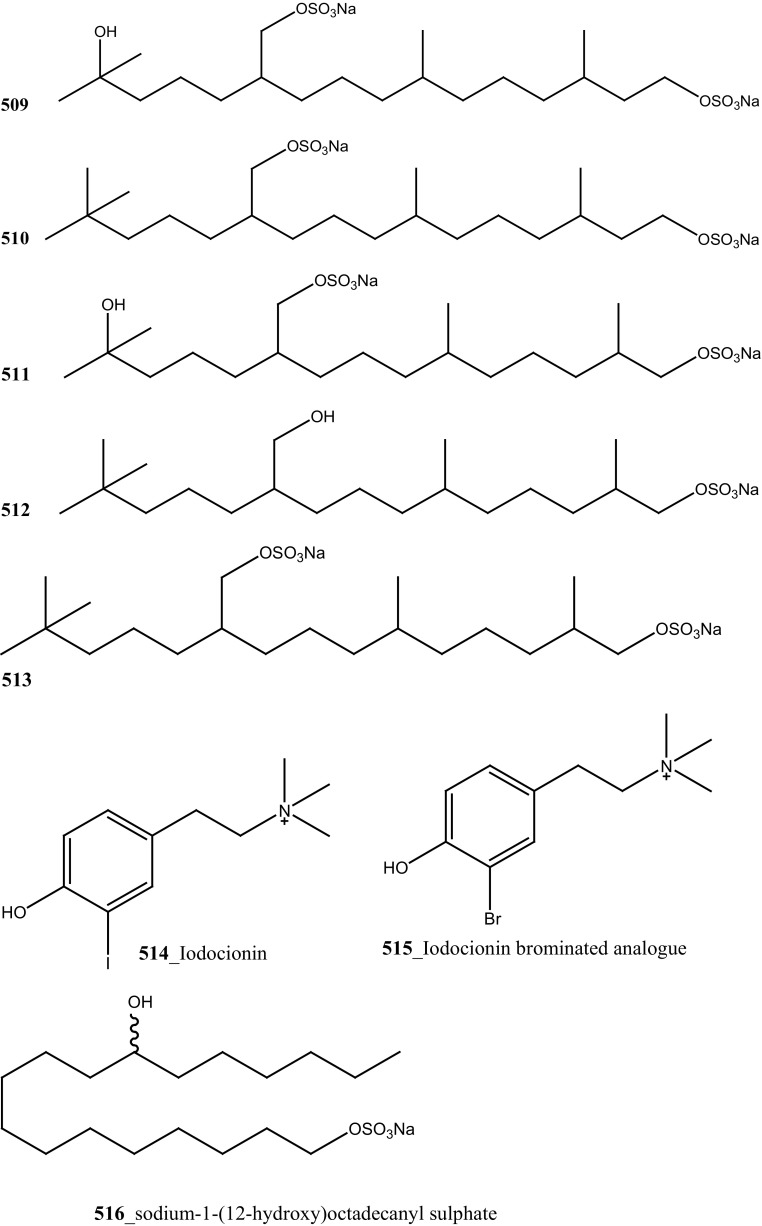

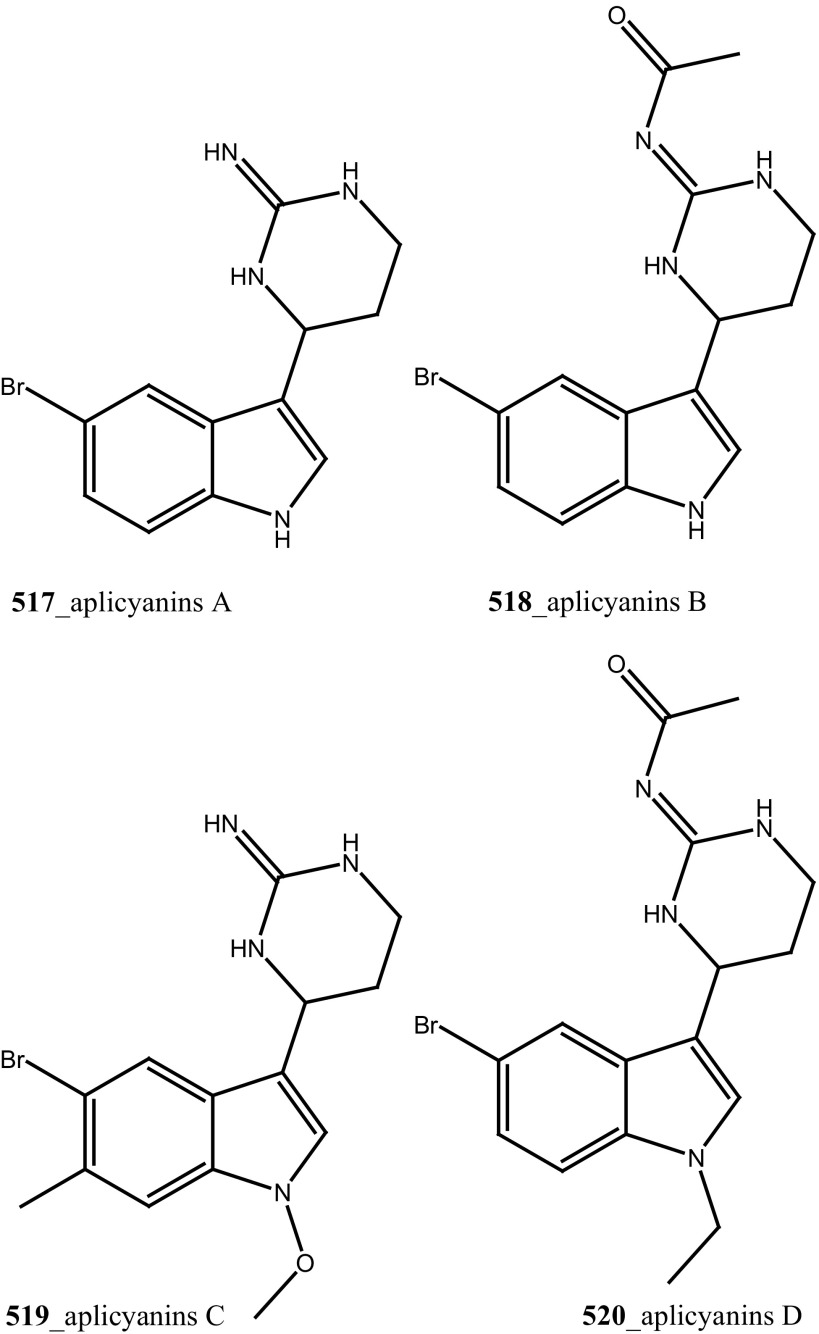

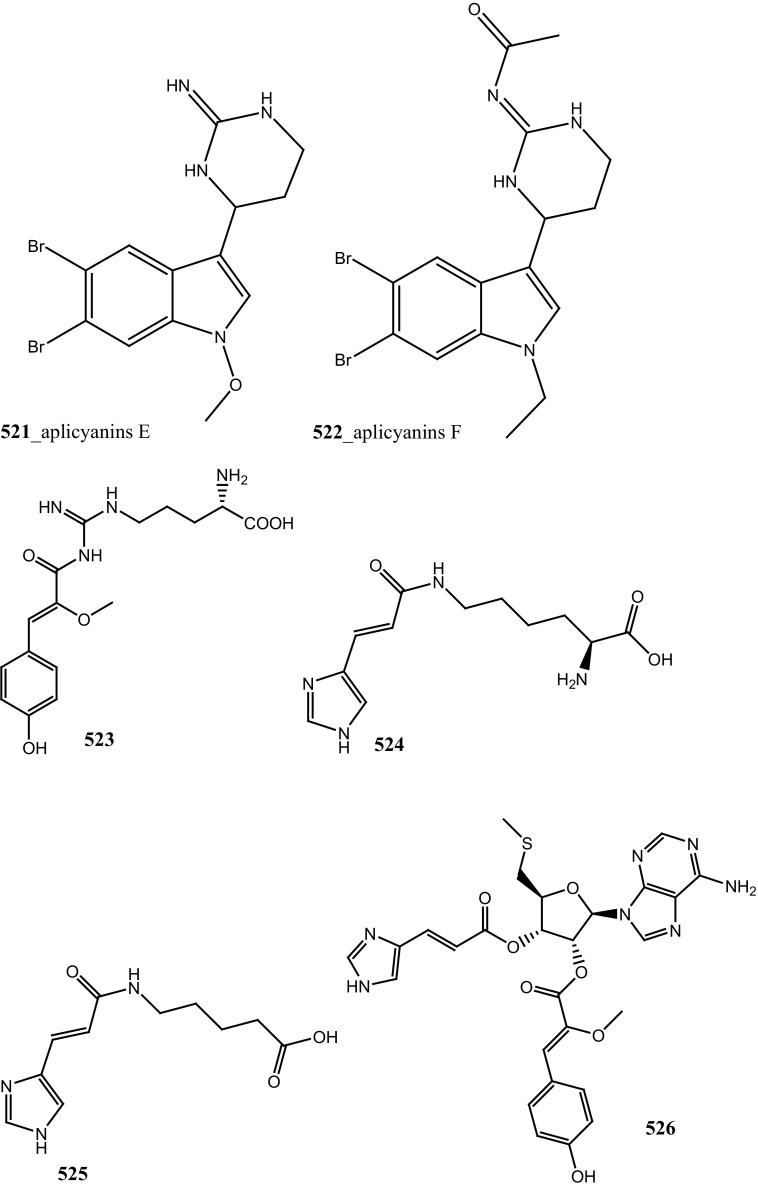

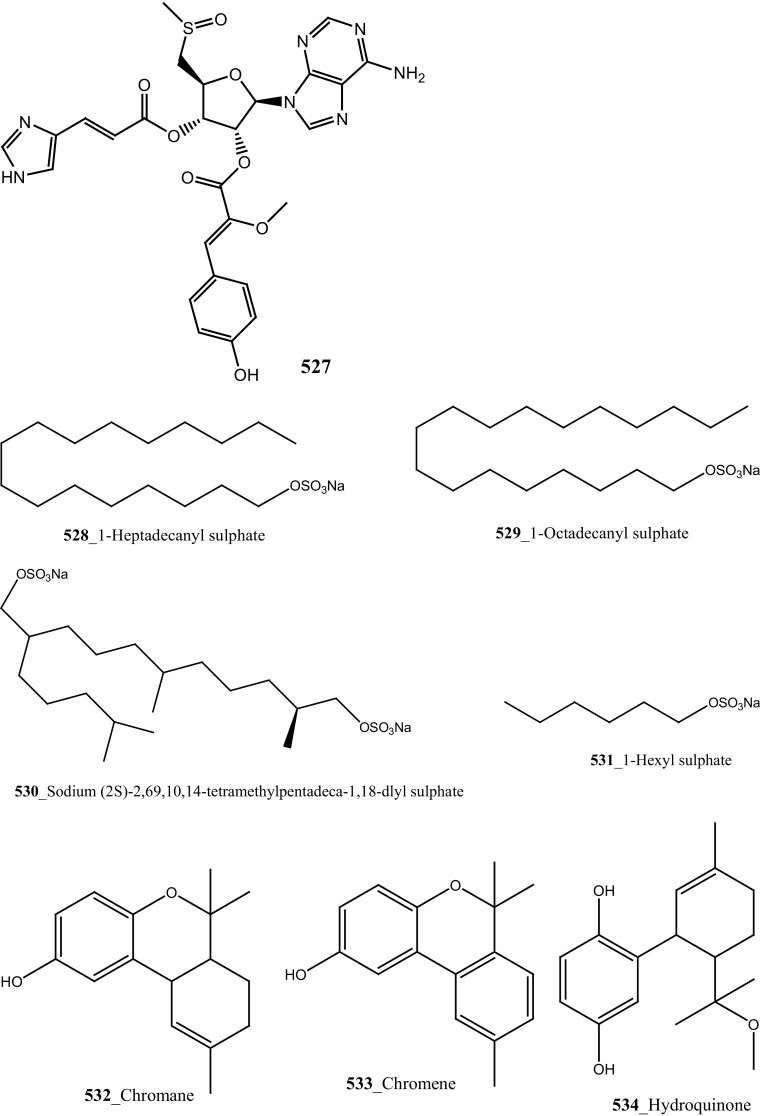

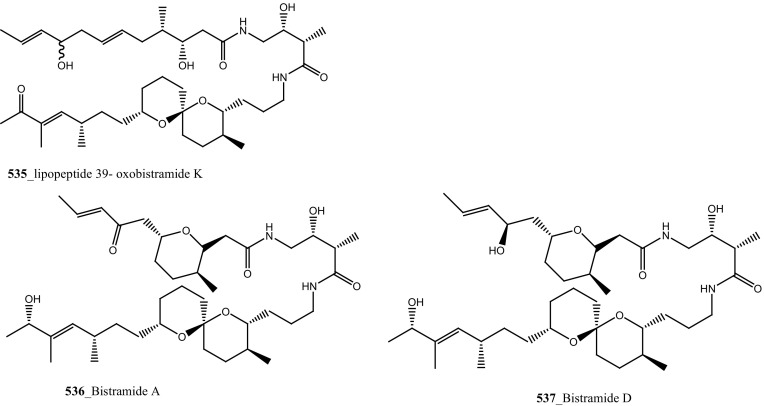

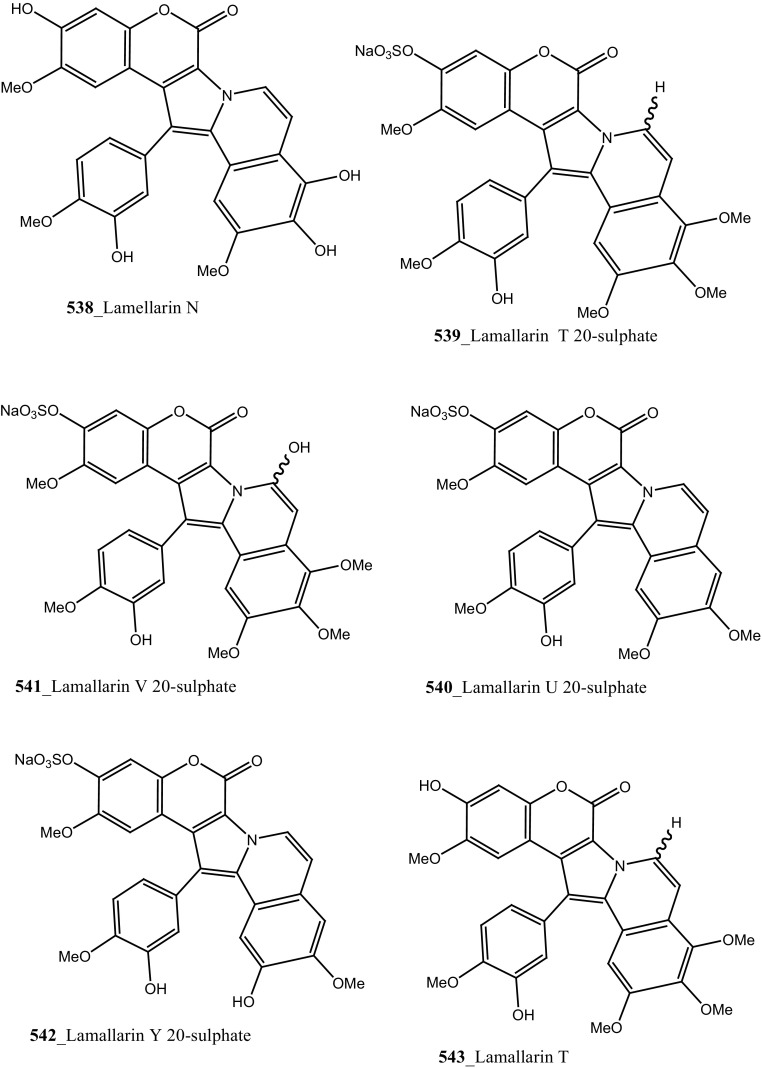

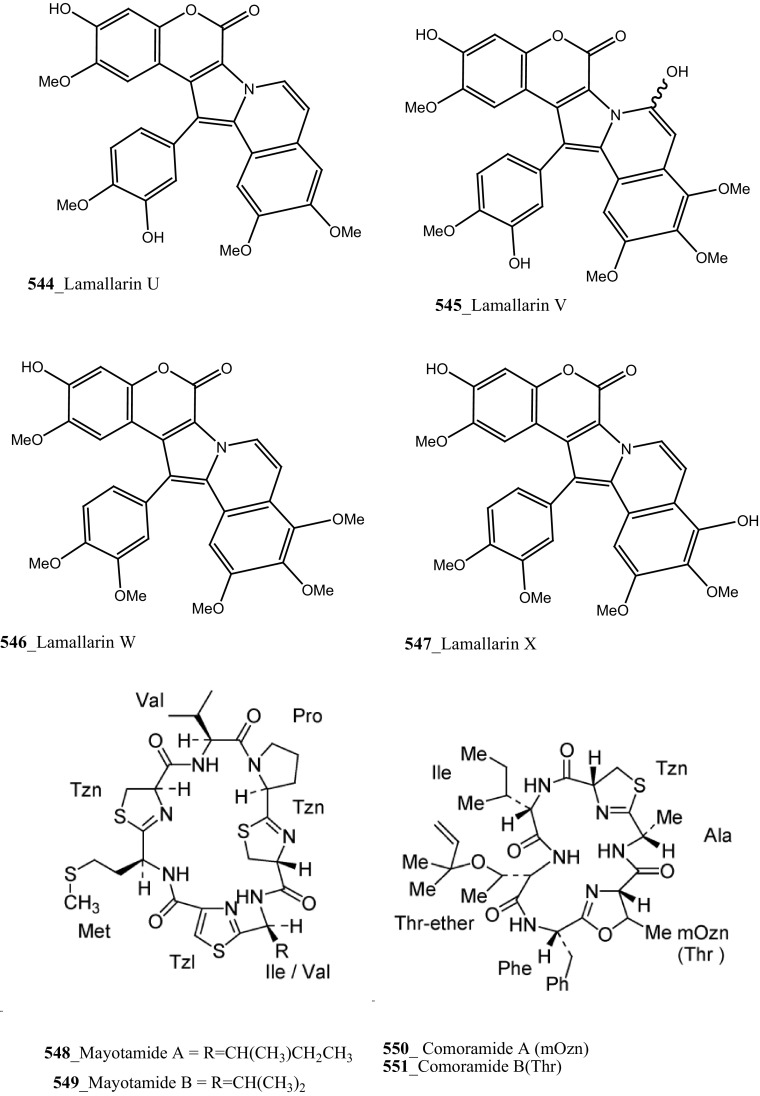

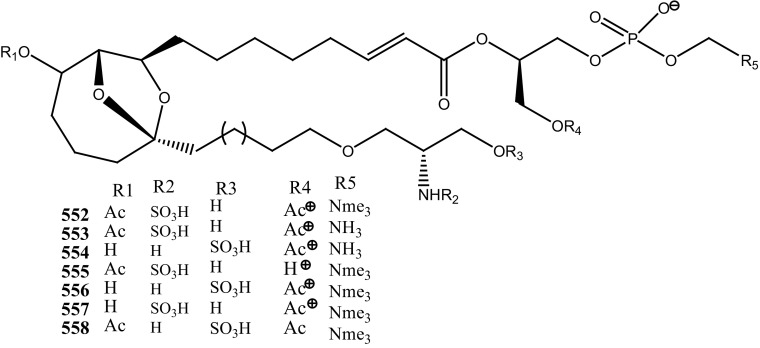
Anti-cancer potential compounds (**237**–**563**)

### Recent Developments and Future Directions

Ascidians have become one of the most vital resources of MNP in the last decades, and more than 800 numbers of compounds with unusual chemical structures have been isolated and their biological activity reported from various ascidian species. Discovery of novel biomolecules are increasing nowadays as we achieved better understanding of marine chemical ecology and their molecular and functional aspects. Progresses in other scientific fields such as chemogenomics, computational biology, genomics, proteomics, metabolomics and fluxomics also have potential applications in ascidian chemical diversity and their biomedical applications.

#### Successful Ascidian Marine Natural Products in Clinical Development

Nature products chemistry research has become much more applied, targeting compounds, which exhibit pharmacologically useful biological activities. Till date there are few metabolites reported from the ascidian were approved by FDA and has reached Phase I, II clinical trials, the list are presented in (Table 1). Didemnin B (**564**) was reported from *Trididemnum solidum* (Family Didemnidae), showed strong anti-viral and invivo cytotoxicity [241, 242]. Total synthesis, complete spectroscopic characterization and single-crystal X-ray structure of metabolite (**564**) was reported [243–245]. Didemnin B was the first MNPs to enter phase I and II clinical trials as an anti-cancer agent. Previous studies demonstrated that compound (**564**) strongly inhibits palmitoyl protein thioesterase in a non-competitive manner [246, 247] while this low affinity target did not completely show inhibition at nano molar concentration. Compound (**564**) had showed potential activity against herpes simplex virus [248] and also against Ehrlich’s carcinoma in mice [249]. During initial cancer trials, didemnin B exhibited modest activity and displayed the constraint for the treatment with anti-emetics [250]. In-vitro test has demonstrated that compound (**564**) was showed potential activity against colorectal cancer cells [251], lymphatic [252] and prostate cancers [253]. In addition, compound (**564**) was submitted to various Phase I trials [250, 254] and Phase II clinical trials against non-small cell lung cancer (NSClC) [255], breast cancer [256] small-cell lung cancer [257], non-Hodgkin’s lymphoma [258], metastatic melanoma [259], glioblastoma multiforme [260], and CNS tumours [261]. All these trials of compound (**564**) caused significant neuromuscular toxicity and no objective tumour responses. However, compound (**564**) was exhibited activity in patients with advanced pretreated non-Hodgkin’s lymphoma, but trials are on hold owing to onset of severe fatigue in patients [262]. Other trials were terminated because of high incidence of anaphylaxis [263], currently all the clinical trials of compound (**564**) are on hold.

**Table 1:** Successful ascidian marine natural product in clinical development

Compound name	Natural product or derivative	Collected source organism	Biosynthetic class of agent	Molecular target	Disease area	Clinical status	Company/institution	References
Ecteinascidin (ET-743)	Natural product	*Ecteinascidia turbinata*	NRPS-derived alkaloid	Minor groove of DNA	Cancer	FDA approved (EU approved only)	Yondelis	279
Plitidepsin (aplidine)	Natural product	*Aplidium albicans*	Cyclic depsipeptide	Rac1 and JNK activation	Cancer	Phase III orphan drug*	PharmaMar	13, 14
Didemnin B	Natural product	*Trididemnum solidum*	Cyclic depsipeptide	Anti-viral agent against DNA and RNA virus	Cancer	Phase III (stopped)	NCI PharmaMar	278
Trabectedin analog (PM01183)	Derivative	Tunicate	NRPS-alkaloid	Minor groove of DNA, nucleotide excision repair	Cancer	Phase I		
Midostaurin	Semisynthetic analogue of 1	Ascidian	Indolocarbazole	Flt-3,PKC, VEGFRs	Cancer	Phase III^a^	NCI	293
Lestaurtinib (CEP-701)	Synthetic analogue of 1	Ascidian	Indolocarbazole	Flt-3, JAK-2, Trk-A, Trk-B, Trk-C	Cancer	Phase III^b^	NCI	295
Edotecarin (J-107088)	Synthetic analogue of 1	Ascidian	Indolocarbazole	Potent stabilizers of DNA topoisomerases	Cancer	Phase III	Pfizer	324
Enzastaurin (LY317615)	Synthetic analogue of 1	Ascidian	Indolocarbazole	PKCβ, GSK-3β	Cancer	Phase III^c^	Eli Lilly	C.T. N NCT00332202
Becatecarin (XL 119)	Synthetic analogue of 1	Ascidian	Indolocarbazole	Potent stabilizers of DNA	Cancer	Phase II/III	NCI	C.T. N NCT00090025
UCN-01	Synthetic analogue of 1	Ascidian	Indolocarbazole	PKC	Cancer	Phase II	NCI	301
CEP-2563 (prodrug of CEP-751)	Synthetic analogue of 1	Ascidian	Indolocarbazole	Trk-A, Trk-B, Trk-C	Cancer	Phase I	Cephalon	308
CEP-1347 (KT7515	synthetic analogue of 1	Ascidian	Indolocarbazole	JNKs	Parkinson	Phase II	Cephalon	C.T. N NCT00040404
Sautosporine (AM-2282)	Synthetic analogue of 1	Ascidian	Indolocarbazole	PKC, JAK2, CamKIII	Cancer	Preclinical	Kyowa Hakko Kirin (originator)	314

*NCI* National Cancer Institute, *CTN* clinical trial number

* Plitidepsin approved orphan drug status by the European Medicines Agency for treating acute lymphoblastic leukemia

^a^Received orphan drug status for treatment of mastocytosis acute myeloid leukemia (FDA, 2009, 2010)NCI, National Cancer Institute; CTN, Clinical Trial number

^b^Received orphan drug status for AML (FDA, 2007)

^c^Orphan drug status for diffuse large B-cell lymphoma (EMEA, 2007)

The toxicities of novel drugs are depending on the concentration of dose including neutropaenia and thrombocytopaenia. Hepatotoxicity was previously observed in preclinical trails but these may be able to control by dose regulation. In recent discovery utilizing HepG2 human hepatocellular liver carcinoma cells have shown a cytochrome P450-mediated metabolism of ET-743 (**565**). The hepatotoxicity was demonstrated in rats that pre-treatment with metabolism modulators such as dexamethasone and β-naphthoflavone abrogates ET-743-arbitrate hepatotoxicity [264], and dexamethasone–ET-473 combination drug treatment was recommended for study in humans [265, 266]. Further, Yondelis^®^ discovered an antitumor agent (Ecteinascidin/trabectedin, ET-743) in a marine colonial tunicate *Ecteinascidia turbinate*, and now produced synthetically, received Orphan Drug designation from the European Commission (EC) and FDA for soft tissue sarcomas and ovarian cancer and its registration in 2007 in the EU for the treatment of soft tissue sarcoma.

However, all the clinical trials with didemnin B are on hold, promising simple analogue of didemnin B, aplidine (dehydrodidemnin B) was first reported from the Mediterranean tunicate *A. albicans* and patent by Rinehart [267]. Aplidine (**566**) are being at variance from didemnin B (**564**) only in replacement of the *N*-lactyl side chain with a pyruvyl group. Compound (**566)** showed identical levels of anti-tumour activity compare to compound (**564**) against several cancer cell lines [263], and also showed improved cell apoptosis by induction of oxidative stress [267], which causes the pro-apoptotic receptor Fas (CD-95) [268] and induces the mitochondrion-mediated apoptosis [269, 270]. Compound (**566)** also activates p38 mitogen-activated protein kinases (MAPKs) and jNK [271, 272], and also inhibits secretion of vascular endothelial growth factor (VEGF) [273, 274]. Non-P-glycoprotein have expressing cell lines that are resistant to compound (**566)**, it demonstrated temporary phosphorylation of jNK and p38 MAPKs upon exposure to aplidine, and the short duration of activation was insufficient to trigger apoptosis [275]. In relapsed–recalcitrant leukaemia cell lines, aplidine arrests the cell cycle at the G1 and G2/M phases, and induces p53-independent apoptosis [276].

Significant potential cytotoxicity of compound (**566)** was noticed against cultured lymphocytes and in transformed cell lines; it is suggesting that during in vivo compounds **(564, 564**) exhibited lowest haemotoxicity [277, 278]. Currently phase III clinical trials of Aplidine with stomach, prostate and bladder cancers. In July, 2003 Pharmamar^®^ was discovered plitidepsin (Aplidine) and received orphan drug status for treatment of lymphoblastic leukemia from European Medicines agency.

Semisynthetic derivative of staurosporine PKC-412 midostaurin (**567**) was from the bacterium *Streptomyces staurosporeus* (isolated from the Ascidian). Compound midostaurin (**567**) is currently in phase III trials under Novartis^®^ for acute myeloid leukemia (AML), and other cancer disease. PKC-412 is a multi-target protein kinase inhibitor being tested for the treatment of myelodysplastic syndrome (MDS), AML and showed strong activity in patients with mutations of CD135 (FMS-like tyrosine kinase 3 receptor) [279]. After successful Phase II clinical trial, a Phase III trial for AML has started in 2008. It is testing midostaurin in combination with daunorubicin and cytarabine (clinical trial number NCT00651261) and in another phase II trial, the substance was proved ineffective in metastactic melanoma [280].

Furthermore, the compound lestaurtinib (CEP-701) (**568**), currently in phase II clinical trials at NCI against relapsed AML and expressing inhibitor of FLT3 among other kinases JAK-2, Trk-A, Trk-B,Trk-C [281]. CEP-701 exhibited most active potential inhibition with JAK-2 kinase inhibitor (IC_50_ 1 nM) [282].

Synthetic derivative of staurosporine, 7-hydroxystaurosporine (UCN-01) (**569**) was isolated from the ascidian *Eudistoma* sp. Compound (**569**) was showed strong anti-tumour activity against several cell lines and potent inhibits kinase activity [283]. UCN-01 shows most potent strong inhibition with many phosphokinases, including the serine/threonine kinase AKT, calcium-dependent protein kinase C, and cyclin-dependent kinases. In phase II study, this metabolite arrests breast cancer cells in the G1/S of the cell cycle and prevents nucleotide excision repair by inhibiting the G2 checkpoint kinase chk1, resulting in apoptosis [284, 285]. Currently compound (**569**) is in phase II trials for develop new therapeutics against several cancers including pancreatic, lymphoma and breast is under process [286–288]. UCN-01 strongly inhibits CHK-1 (*ki* = 5.6 nM), PDK-1 (IC_50_ = 5.0 nM), and PKC*β*37 (IC_50_ = 10 nM) [142–144]. It also inhibits CDKs CDK-1 (*ki* = 95 nM), CDK-2 (*ki* = 30 nM), and CDK-4 (*ki* = 3.6 µM), and isoforms of PKCs, with an IC_50_ of less than 1 µM [289–291].

An Ascdiian derivative, becatecarin (**570**) is a synthetic diethylaminoethyl analogue of the indolocarbazole glycoside antineoplastic antibiotic rebeccamycin. Compound (**570**) stimulated ATPase activity and inhibited ABCG2 arbitrated transport at concentration >10 µM and induced ABCG2 expression in lung cancer cells. In phase I trial were tested in children with solid tumours to establish the dose limiting toxicity and maximum tolerated dose [292]. In phase II trial, cecatecarin (**570**) intercalates into DNA and stabilizes the DNA-topoisomerase I complex, thereby interfering with the topoisomerase I-catalyzed DNA breakage-reunion reaction and initiating DNA cleavage and apoptosis [293]. Compound (**570**) is reached Phase III trials in Helsinn and Excelis but now is quoted as being at phase II level, though a search of the NCI clinical trials databases (clinical trial NCT00072189) shows 17 completed trials but all the trails has been terminated since February 2015.

Ascidian derivatives CEP-2563 dihydrochloride (**571**) is a soluble lysinyl-beta-alanyl ester of CEP 751. Compound (**571**) is belongs to the same class of molecules as CEP-701 and it can be easily converted into the latter by O-demethylation. Compound (**571**) posses’ inhibitory activity against several tumours cell (medullary thyroid carcinoma) and blocks certain proteins involved in the growth of some tumors and kill cancer cells [294]. It is also behave a type of receptor which includes the tyrosine kinase inhibitor Trk-A, Trk-B,Trk-C and platelet-derived growth factor (PDGF) [295]. CEP-2563 was evaluated Phase I clinical trial in patients with advanced stage solid tumours. Undevia and co-authors have been demonstrated Phase I clinical trial to determine the cytotoxicity profile, maximum tolerate dose and pharmacokinetics of CEP 2563 in 18 patients [295]. This investigation demonstrates that single agent CEP-2563 therapy is achievable within recommended toxicity level, the suggested phase II dose concentration is 256 mg/m (2)/d.

CEP-1347 (571) is an indolocarbazole kinase inhibitor originally discovered by Kyowa Hakko Kogyo in the course of a program investigation of neurotrophic properties of derivatives of the natural product K-252a [296]. These are ploycyclic aromatic compounds containing an indole fused to carbazole. CEP-1347 demonstrated that apoptosis with multiple nerve cell types from a variety of agents leading to programmed cell death which significantly increase the dopamine neurons survival prior to and afterward transplantation. CEP-1347 blocks the activation of JNKs through ATP competitive inhibition of the upstream mixed lineage kinase (MLK) family. CEP-1347 had showed prominent neurotrophic and neuroprotective properties in-vitro and in animal models of neurodegeneration [297, 298]. In particular, this inhibitor was able to reduce the loss of tyrosine hydroxylase immunoreactivity and dopamine transporter density in mice and monkeys following administration of the neurotoxin 1-methyl-4-phenyl-tetrahydropyridine (MPTP) [298, 299]. Unfortunately, the direct effect of CEP-1347 administration on inhibition of the MLK/JNK pathway in central nervous system of human subjects could not be determined in PRECEPT trial. Therefore, the failure of the PRECEPT trial has limited utility for assessing the relationship between JNK activity and neurodegeneration [300]. Preclinical trials indicated it was a neuroprotective drug, currently all the clinical trials of CEP-1347 were terminated on 2012 (clinical trial number NCT00040404).

Alkoid compound saturosporine (AM-2282 or STS) (**572**) is an indolocarbazoles belongs to the alkaloid sub-class of bisindoles. AM-2282 is the precursor of the novel protein kinase inhibitor midostaurin (PKC412, JAK2, and CamKIII) [301]. Compound **572** showed most potent inhibition with cell permeable inhibitors of protein kinases (IC_50_ 0.7–20 nM), protein kinase C from rat brain (IC_50_ of 2.7 nM) and strong inhibitory effect against HeLa S3 cells (IC_50_ of 4 nM) [302, 303]. Compound **572** also showed strong inhibition with several other protein kinases such as:PKA, PKG, phosphorylase kinase, S6 kinase, Myosin light chain kinase (MLCK), CAM PKII, cdc2, v-Src, Lyn, c-Fgr, and Syk with IC_50_ values of 15, 18, 3, 5, 21, 20, 9, 6, 20, 2, and 16 nM, respectively [304]. Staurosporine AM-2282 (**572**) induced >90% apoptosis in PC12 cells at concentration 1 µM. Although, AM-2282 treatment induces a rapid and prolonged elevation of intracellular free calcium levels [Ca^2+^]_i_, accumulation of mitochondrial reactive oxygen species (ROS), oxidative stress and subsequent mitochondrial dysfunction [305]. The apoptosis of MCF7 cells induced by staurosporine can be enhanced by the expression of functional caspase-3 via caspase-8 activation and bid cleavage [306]. Staurosporine induces apoptosis of human foreskin fibroblasts AG-1518; depending on the lysosomal cathepsins D mediated cytochrome c release and caspase activation [307]. Additionally, to initiating the classical mitochondrial apoptosis pathway, staurosporine activates a novel intrinsic apoptosis pathway, relying on the activation of caspase-9 in the absence of Apaf-1 [308]. Li et al. [309] were reported staurosporine induces apoptosis with unexpected cholinergic effects in SH-SY5Y cell line at 100 nM concentration. Also staurosporine decreased acetylcholinesterase enzymatic activity (AChE) and decreased protein levels of the AChE splice variant tailed (AChE-T) (Structure 11).

**Structure 11:**
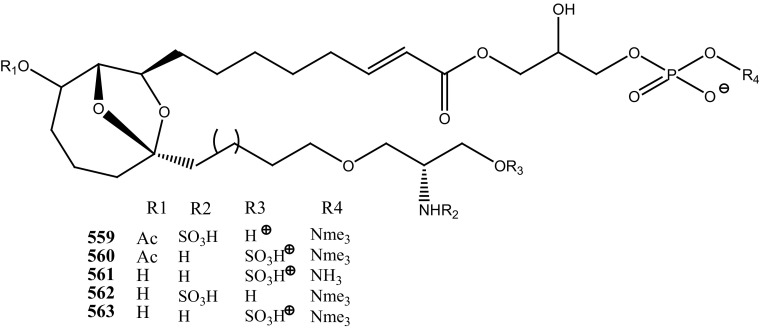

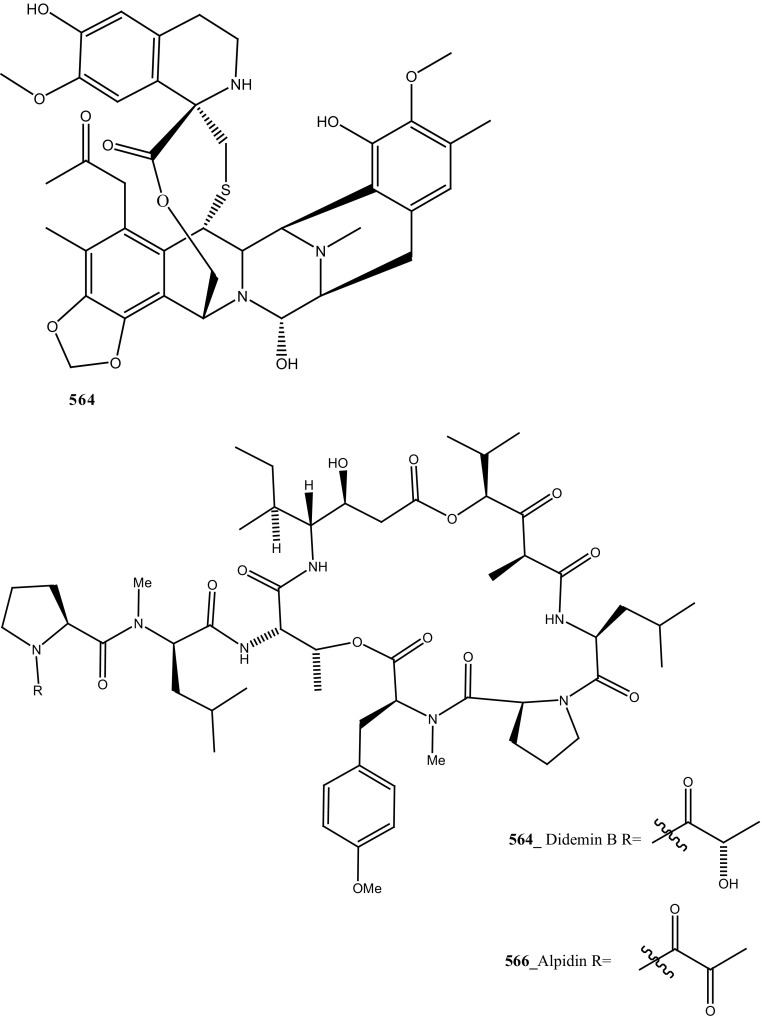

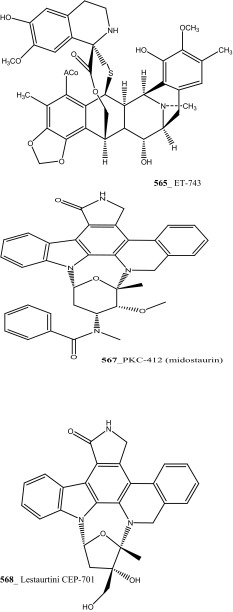

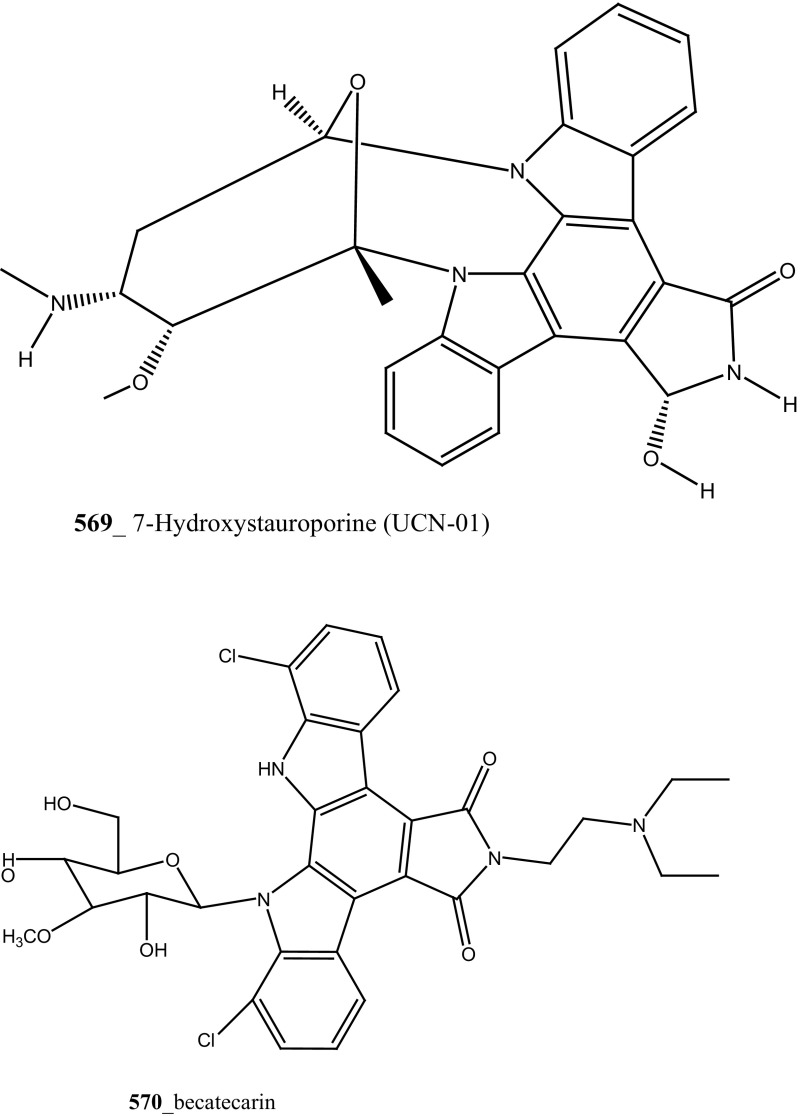

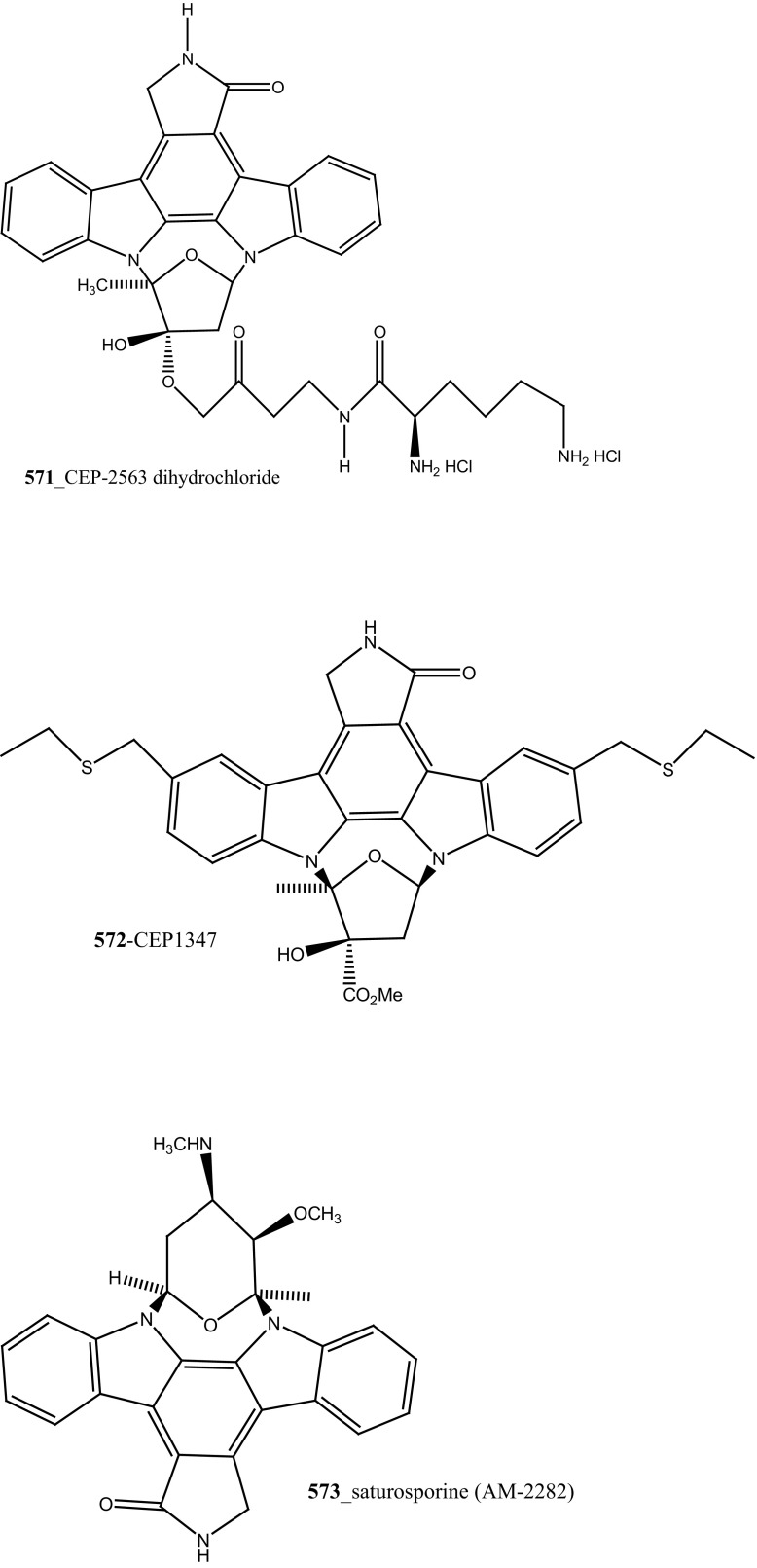
Successful ascidian marine natural products in clinical development (**564**–**573**)

#### Modern Instrumentation and Computational Biology

Marine ecosystem forms an important source of unique compounds with high structural uniqueness and incomparable chemical properties. At the core of MNPs discovery is the identification procedure and NMR stays on the most useful tool [310]. At this time, natural products chemistry research is progressing a dynamic comeback in the modern drug discovery. Relatively more advances have taken place concerning the inherent capabilities of NMR apparatuses, able to reduce experiment times and increase sensitivity toward more efficient analyses of novel compounds present in µM level [311]. The advance of high resolution magic angle spin NMR (HR-MAS NMR) probes is most useful to analyse intact tissues. Nevertheless, while (HR-MAS NMR) is incorporated in food chemistry where both primary and secondary metabolites are of importance, it has not been yet widely introduced in natural products chemistry [312].

Queiroz Junior et al. have impressively demonstrated the significance of the synergy between NMR hardware and innovative pulse sequences. It is the first report that an ultrafast COSY pulse sequence is applied to a hyphenated LC–NMR separation of crude extracts (Ex. three natural flavonoids; naringin, epicatechin and naringenin). The detection volume was only 60 mL, while two scans have proven sufficient to get spectra with optimized resolution and sensitivity. This application portrays the generality of ultrafast methodologies in natural product chemistry, placing LC–NMR as an effective analytical tool [313, 314]. For example, NMR experiments such as DOSY and JRES were also very useful routine methodology for unraveling new chemical structures. NMR spectroscopy tirelessly continues leading this procedure. Furthermore, decisive chemical structural information could be derived from statistical interpretation methods applied in metabolomics such as statistical heterospectroscopy (SHY) [315], statistical total correlation spectroscopy (STOCSY) [316], heteronuclear single quantum correlation spectroscopy (HSQC), heteronuclear multible-bond correlation Spectroscopy (HMBC), Subset optimization by reference matching (STORM) [317], cluster analysis statistical spectroscopy (CLASSY) [318], multivariate statistical analysis of natural products fragments [319].

Nearly, dereplication analysis is necessary for computational support of data handling, processing and for structure elucidation purpose. Whereas user-friendly and sophisticated software packages are easily reached for effective data mining, they are not widely used for dereplication purposes in marine natural product chemistry [320]. However, the structure elucidation is most challenging task mainly due to uniqueness of natural products and unexpected spectral patterns and the residual complexity frequently noticed. For instance, prediction and simulation software such as PERCH, in combination with ^1^H iterative full spin analysis (HiFSA approach), given an accurate distinction of natural products with nearly identical NMR spectra. As, proposed by the authors as a tool for puzzling qNMR analyses, it could be an alternative source of dereplication data [321]. Moreover, computer-assisted structural elucidation (CASE) is the techniques of using software that allows users to input their NMR data, and through matching algorithms to generate all possible molecular structures. For this purpose, software used are mainly the Structure Elucidator by ACD Labs, StrucEluc and CCASA [322–324]. Nevertheless, the success of these approaches is dependent on the quality of the spectra to be processed and the efficacy of the algorithms used. Furthermore, the software used present an inherent dependence on the databases from which data are extracted. Unfortunately, NMR databases dedicated to NPs appear as in-house, fragmented attempts, or are chemical group/organism/NMR experiment/solvent, among others, specific. For instance, MarinLit, and AntiBase, specialize in marine, fungal and microorganism NPs, NAPROC-13 is based on 13C resonances [325], while recently compiled TOCCATA uses 13C-labeled NPs [326]. Commercial NMR databases are limited to few vendors, like the SpecInfo database of Wiley and Bruker’s NMR database [327, 328].

#### LC–MS and ^1^H-NMR Metabolomics

Recent developments in analytical methods have resulted in many different platforms for metabolomic investigation. From these, liquid chromatography–mass spectrometry (LC–MS) [329], and nuclear magnetic resonance spectroscopy (NMR) based approach are generally preferred analytic methods because they are based on the physical properties of MNPs, which are not influenced by other external factors and easily reproducible [330]. In the recent years, NMR combined with metabolomics tool is increasingly utilized for its systematic manner of profiling chemical finger prints of individual samples, either plant or animals [329, 330]. NMR-metabolomics snap shots the organism’s metabolites or biomolecules that are present in a given quantity at the given time point [331, 332]. Metabolomics can be used in functional genomics and to differentiate marine organism from external variation. The metabolomics of biota is compilation of all its primary and secondary metabolites using ^1^H-NMR and 2D-COSY spectroscopy methods. Kim and co-authors reported the protocol NMR based metabolomics of plant species [332]. Tikunov et al. [333] carried out study of taxonomy based metabolite profiling of an oysters using NMR metabolomics along with Multivariate Statistical Analysis Approach (MSAA). Additionally, in manila clam [334], corals [335], and LC–MS based metabolomic approach in marine bacteria [336], studies utilized the same methodology for classifying biomolecules based on their taxonomy. In earlier study, Halouska and co-workers [337] predicted the in-vivo mechanism of action for drug leads against anti-tubercular activity using NMR metabolomics and orthogonal partial least square-discriminant analysis (OPLS-DA).

Mass spectrometry based metabolomics approach can provide significant information about the discrimination between the species using multivariate statistical analysis, classifying chemical groups, discriminate the metabolites with unknown biological potencies [338]. A typical metabolomics profiling requires enormous number of samples to generate the results that are statistically rigorous. Besides, highly sensitive and accurate instrumentation, powerful software tools (e.g. XCMS-METLIN) are essential to address the vast amount of data generated by these experiments [339]. The recent development in the field of natural products chemistry and LC–MS/NMR based metabolomics research on marine origin secondary metabolites exhibits diverse range of biological properties for developing new therapies to improve the health of individuals across the universe suffering from various deadly diseases such as infectious disease malaria, HIV, neurological and immunological diseases and cancer [329, 330, 339]. The application of LC–MS based metabolic profiling of biological systems has gained more extensive use in identifying drug metabolite, developing metabolite maps and lending clues mechanism of bioactivation [338]. However, the knowledge of the metabolite accumulates in different ascidians chemical diversity are meagre. Recently, Palanisamy et al. [340] reported the metabolic profiling of invasive ascidian *S. plicata* and Mediterranenan ascidian *A. mentula* collected in Messina coast using LC–MS and multivariate statistical analysis. The results of this study confirmed that LC–MS based metabolomics method could be used as reliable tool for taxonomic classification of marine ascidian species and species discrimination in future studies. Ascidian, *S. plicata* showed significant anti-microbial activity against *Burkholderia mallei* (10 mg/mL) [341], and *S. plicata* fraction SP50 exhibited strong inhibition and induced apoptosis against cervical carcinoma (HeLa) and colon carcinoma (HT29) with IC50 (33.27 and 31.66 μmol/L).

#### Recent Biotechnology Advances

In a new marine drug discovery approach, structurally more complex MNPs was moved the next step from discovery to clinical trials based upon the strength of the industrial reproducibility. The discovery of novel marine drugs will continue to diversify. Research laboratories, academic entrepreneurs, and innovative biotechnology industries will play major role in the discovery of novel marine drugs. The industrial collaboration program between natural products researchers and biotechnology industries will be instrumental to the primary clinical trials and mechanism of action studies crucial to provide the compelling preclinical data to create ample interest from larger pharmaceutical companies to lead and support for drug discovery program of MNPs. Also, it is essential to identify molecular targets for strongly active biomolecules and the ability to synthetically produce novel biomolecules to progress and discover new drugs.

A recent development in biotechnological approach is revolutionizing the field of natural products chemistry. It is worth to mention here, during the isolation process was able collect only tiny amount strongly active biomolecules. It is very hard to collect in required quantity, the advanced techniques and the availability of new methods in chemical and biological synthesis have provided access to even the most complex of drug lead structures. High advanced developments in analytical tools and molecular biological science facilitate to identify the primary producers of secondary metabolites from symbiotic assemblage, and enable researchers to further explore the marine microbial chemical diversity for drug like biomolecules. Furthermore, those advances aide the characterization of several biosynthetic gene clusters and pathways and ultimately allow for their manipulation. Marine microbial chemical diversities are now easily explored drug like compounds using effective biosynthetic genetic engineering and in vitro multi enzyme synthesis methods [342]. Remarkably, David Hopwood’s group [343] has biosynthesized anti-biotic compound actinorhodin from *Streptomyces coelicolor* by cloning and heterologous expression of an entire biosynthetic pathway. Using genetic engineering techniques, Donia et al. [344] were prenylated anti-tumor compound trunkamide which is previously isolated in ascidian and different genera of cyanobacteria in *E. coli* culture. Didmnid ascidian species specificity of symbiosis and secondary metabolism in ascidian species were reported [344], collected in Florida coast. In this study, species specific and location-specific components were observed in Dideminid ascidian microbiomes and metabolomes. It is concluded that the biotechnological approaches in the field of natural products chemistry is more useful for sustainable supply of high quality marine drugs.

## Conclusions

This review study represents trends in chemical diversity of marine ascidians and potential biomolecules, covering the various tunicates family, recent developments and future direction and modern biotechnology advances are highlighted. Remarkably, Genus *Didmnium* sp. is most studied species in this group followed by *Aplidium* sp., *Synoicum* sp., and *Eudistoma* sp. collected from coral reefs, intertidal regions, shallow water and mangrove ecosystem which facilitates potential bioprospecting. Several MNP isolated from ascidian that are in various phase of pre-clinical and clinical studies from that Ecteinascidia and aplidine have great potential to reach market.

Anti-cancer drugs are the main area of interest in the screening of MNPs from ascidians (64%), followed by anti-malarial (6%) and remaining others. It is worth to note here, as the major financial support for the screening of new MNPs is made in cancer research [344]. The data discovered here undoubtedly confirmed that promising value of MNPs and their derived analogs are most important candidates for further pharmaceutical studies for discover new therapeutic treatment the anti-tumor/anti-cancer Anti-HIV and various diseases drug pipeline.

The unique chemical structures and novel chemical class of ascidians and promising biological activity which make them excellent candidates for development of many first class marine drugs in the near future with current advanced sampling methods, highly advanced analytical tools, new methods for genetic, chemical dereplication, molecular biology tools, LC–MS, NMR metabolomics approach, nature bank databases, computational biology, directed biosynthesis and biosynthetic pathway and high throughput screening the efficiency of exploring MNPs to discover novel therapeutics has increased significantly. It is concluded from this study, Ascidian resources contains vast pool of novel metabolites, exploring drug-like biomolecules will provide promising biomolecules with potential therapeutic use which may serve as lead candidates for drug discovery program.
